# Analysis of RNA Transcribed by RNA Polymerase III from B2 SINEs in Mouse Cells

**DOI:** 10.3390/ncrna11030039

**Published:** 2025-05-14

**Authors:** Olga R. Borodulina, Sergey A. Kosushkin, Ilia G. Ustyantsev, Nikita S. Vassetzky, Dmitri A. Kramerov

**Affiliations:** 1 Laboratory of Eukaryotic Genome Evolution, Engelhardt Institute of Molecular Biology, Russian Academy of Sciences, Moscow 119991, Russia; olgarb@mail.ru (O.R.B.); ustian@mail.ru (I.G.U.); nvas@eimb.ru (N.S.V.); 2 Independent Researcher, Moscow 119991, Russia; toki@mail.ru

**Keywords:** SINE, retrotransposon, RNA polymerase III, transcription terminator, polyadenylation, non-coding RNA, mouse genome

## Abstract

**Background/Objectives:** SINEs (short interspersed elements) are eukaryotic non-autonomous retrotransposons. They are transcribed by RNA polymerase III (pol III) and generate non-coding RNAs. The 3′ end of many mammalian SINEs contains a polyadenylation signal (AATAAA), a pol III transcription terminator, and an A-rich tail. Studies have shown that, in human HeLa cells that have been transiently transfected with such SINEs, short pol III-generated SINE transcripts undergo polyadenylation, resulting in the addition of a long poly(A)-tail. Notably, this AAUAAA-dependent polyadenylation is not characteristic of any other transcripts synthesized by pol III. B2 SINEs, found in the genomes of mouse-like rodents, exemplify all these features. **Methods:** In this study, we implemented a novel approach to sequencing pol III-generated B2 transcripts from mouse cell cultures (L929 and 4T1) and organs (brain and testis). **Results:** Transcription occurred in 16,000–20,000 B2 copies in each cell type, 51–62% of which were transcribed in all four cell types. Effective transcription terminators (e.g., TCT_>3_ and T_≥4_) were found in approximately 40% of the transcribed B2 copies. The transcripts of these B2 copies contained a truncated terminator sequence, as pol III transcriptional arrest is known to occur within the terminator, with a poly(A)-tail immediately downstream. Such a tail could only have formed through RNA polyadenylation. **Conclusions:** These results demonstrate that B2 transcripts synthesized by pol III are capable of polyadenylation in mouse cells. We discuss the transcription of B2 copies with and without moderately efficient pol III terminators (TCTTT) and provide examples of the polyadenylation of such transcripts.

## 1. Introduction

Short interspersed elements (SINEs) are non-autonomous mobile genetic elements that are 100–600 bp in length and are transcribed by RNA polymerase III (pol III) (reviewed in [1,2,3]). SINEs are characteristic of most multicellular organisms, and the number of their copies in a genome can reach a million. The emergence of new copies of SINEs in genomes is facilitated by a process known as the reverse transcription of SINE RNA (retrotransposition), which is catalyzed by an enzyme encoded within long interspersed elements (LINEs) that are present in the same genomes. Notably, several families of SINEs, particularly those belonging to primate Alu and rodent B1, have their origins in 7SL RNA, a component of signal recognition particles. Furthermore, a number of SINE families from across diverse animal groups have emerged from 5S rRNA sequences. However, the majority of SINE families are derived from tRNAs, resulting in the 5′-terminal (head) parts of SINEs exhibiting notable similarity to the nucleotide sequences of tRNA species. It has been observed that both tRNA genes and SINEs that originate from these RNAs are transcribed by pol III due to the presence of an internal promoter of this RNA polymerase. This promoter comprises 11-nucleotide boxes, A and B, which are separated by a 30–40 bp region. The 3′-terminal portion of SINEs plays a crucial role in the recognition of their transcripts by the reverse transcriptase, which is encoded by LINEs. In the context of placental mammals, the majority of SINEs utilize LINE-1 (L1) reverse transcriptase for their retrotransposition, a process that necessitates the presence of a poly(A)-tail at the SINE extremity.

SINEs have been demonstrated to play an important role in the evolution and function of genomes. When SINEs are integrated into genes, including their introns and regulatory regions, gene function can be disrupted, i.e., mutations can occur [2,4]. Conversely, numerous other insertions of SINEs into introns or regulatory regions have been observed to exert no deleterious effects on gene function. Rather, these insertions have been shown to enhance it by modulating gene transcription [5,6,7] or mRNA splicing [8,9] and polyadenylation [10,11]. SINE transcripts that are synthesized by pol III are non-protein-coding RNAs. They have been observed to interact with specific chromosome regions [12]. These transcripts have also been shown to interact with RNA polymerase II (pol II) [7,13] and glucocorticoid receptor [14]. This interaction suggests a potential regulatory role of SINE transcripts in the transcription of protein-coding genes.

B2 was the first cloned and sequenced short repetitive element [15,16], and was later recognized as tRNA-derived SINE [3,17,18]. The B2 family has been identified in three related rodent families, Muridae (mice, rats, and gerbils), Cricetidae (hamsters, voles, and lemmings), and Spalacidae (blind mole-rats and bamboo rats) [19]. B2 can be divided into five subfamilies (a, b, c, d, and e). Copies of the classical mouse B2 belong to the B2a subfamily [19] (previously recognized as the four subfamilies Mm1a, Mm1t, Mm1o, and Mm2 [20,21]). The B2a subfamily is found exclusively in murids, where it is the youngest and most abundant. The genomes of *Mus musculus* and *Rattus norvegicus* contain at least 59,000 and 85,000 copies of B2a, respectively [19]. Henceforth, B2a copies will be designated as B2 (with exceptions made for B2 subfamilies in relevant discussions).

The seminal studies on B2 demonstrated that the 3′-terminal segment of this SINE generally comprises several AATAAA hexamers, TCT_3─5_ (a pol III transcription termination signal), and an A-rich tail at its terminus [15,16]. B2 transcription was shown to be catalyzed by pol III in a cell-free system, and its transcription was halted at the terminator signal [15,22]. Mouse tumor cells were found to contain significant amounts of B2-specific RNAs of variable length (from 200 to 400 nt) [23]. These B2 RNAs were found to bind to poly(U) or oligo(dT), suggesting the presence of poly(A) in B2 RNAs [23,24]. Consequently, it was hypothesized that B2 is actively transcribed by pol III in mouse tumor cells and that the resulting transcripts are polyadenylated, as suggested by the polyadenylation signal (PAS) near their 3′-end, AAUAAA [23]. It should be noted that this hypothesis was highly speculative due to the fact that AAUAAA-dependent polyadenylation had only been previously described for transcripts synthesized by pol II, which significantly differs from pol III in terms of its structure and properties. Subsequent studies further validated the synthesis of cellular B2 RNA by pol III and demonstrated that this RNA contains poly(A) segments of varying lengths, thereby providing a plausible explanation for the observed length heterogeneity of B2 RNA molecules [24,25].

A number of SINE families have been described in the genomes of mammals whose 3′-terminal regions have been shown to resemble those of B2; namely, they contain an AATAAA sequence followed by a potent pol III transcription terminator (T_≥4_ or TCT_≥3_). Such elements have been termed T^+^ SINEs [26]. The proposed ability of pol III-derived transcripts to be AAUAAA-dependently polyadenylated was extended to all T^+^ SINEs. Further insight into this hypothesis was obtained by the transfection of human HeLa cells with copies of B2 and other T^+^ SINEs [19,27]. These studies demonstrated that the pol III transcription of these SINEs terminates at the terminator, resulting in a shortened RNA terminator sequence. Poly(A) is then synthesized at the 3′-end of the resulting transcripts. Nucleotide substitutions in AATAAA completely abolished all polyadenylation, thereby indicating that this sequence serves as a PAS, as in mRNA. Four SINE families, including B2, were utilized to identify two additional sequences (β and τ signals) that are critical to the polyadenylation of their transcripts. The former is located downstream of the B box of the pol III promoter, and the latter precedes the PAS [27,28]. T^+^ SINE transcripts involve the mPSF protein complex in their polyadenylation, in which this complex, as in the case of pre-mRNA molecules, recognizes the PAS and triggers poly(A) synthesis [29,30]. Additionally, the τ signal in the B2 transcript has been identified as being essential to its interaction with the auxiliary factor CFIm, which is a polyadenylation factor of many human cellular mRNAs [29].

The role of poly(A) at the 3′-ends of T^+^ SINE transcripts remains to be fully elucidated. Poly(A)-containing B2 RNA molecules exhibit a significantly longer lifespan in mouse cells compared to primary B2 transcripts [24]. Transfection experiments in which HeLa cells were transfected with various T^+^ SINEs, including B2, have demonstrated that their polyadenylated transcripts exhibit significantly higher stability than their poly(A)-lacking counterparts [31]. In SINE transcripts, long poly(A) tail (A_>20_) is strictly required for retrotransposition mediated by L1 reverse transcriptase to occur [32]. It has been observed that numerous SINEs, including human Alu, employ a poly(A)-tail that is encoded in genomic DNA for this purpose [2,33,34]. The mechanism of A-tail elongation and/or length maintenance is based on the phenomenon of DNA template slippage that occurs in reverse transcription [35]. We hypothesize that T^+^ SINEs may also employ an alternative strategy, namely the polyadenylation of SINE transcripts [19].

The preceding data, derived from the introduction of individual copies of T^+^ SINEs into heterologous (human) cells, unequivocally demonstrate the polyadenylation competence of pol III transcripts of these SINEs. Nevertheless, no equally compelling evidence for the polyadenylation of pol III transcripts derived from T^+^ SINEs in their native cells has been obtained thus far. Numerous SINE copies are transcribed in homologous cells. These copies differ from each other, for example, in the presence or absence of terminators or transcribed A-rich tails, making the analysis of their transcripts technically challenging.

Their analysis is further complicated by the fact that transcripts synthesized by pol II (pre-mRNAs and long non-coding RNAs) are also rich in B2 sequences that are found in the introns of protein-coding genes as well as in their 3′-terminal untranslated regions. In this work, we constructed cDNA libraries through the reverse transcription of B2 RNA derived from different mouse cell cultures and tissues. These libraries were sequenced and the resulting cDNA reads were assigned to particular B2 copies in the mouse genome. The sets of copies transcribed in different cell types were then analyzed. A comparison of the 3′-terminal sequences of genomic copies of B2 with their corresponding cDNA reads was performed to analyze the termination of pol III transcription and to directly confirm the polyadenylation of the resulting transcripts in mouse cells.

## 2. Results

### 2.1. Detection of B2 RNA in Mouse Cell Cultures and Tissues

**Figure 1:**
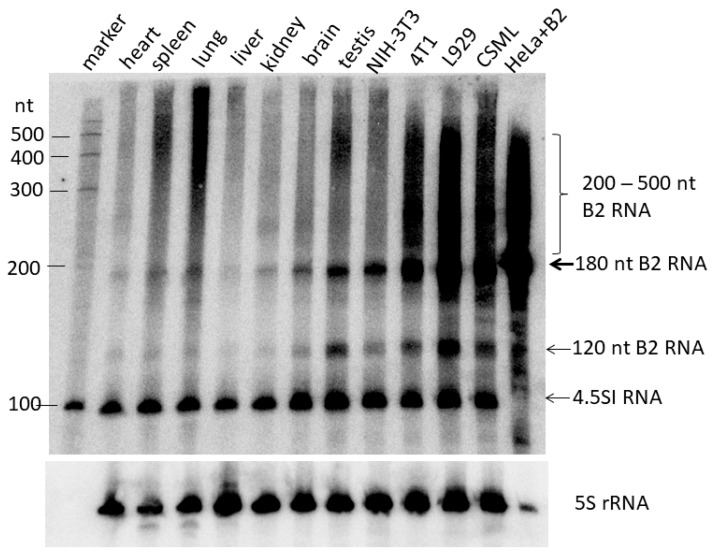
Northern hybridization of RNA isolated from mouse tissues and murine cell cultures. Arrows indicate primary B2 transcript (180 nt), processed B2 transcript (120 nt), and 4.5SI RNA (transcript of a B2-related gene). The presumable polyadenylated B2 transcripts are marked with a curly bracket. The last lane shows RNA from human HeLa cells transfected with a cloned copy of SINE B2. 5S rRNA hybridization was used to ensure equal quantities of mouse cellular RNA were loaded (loading control).

The presence and relative content of B2 RNA (i.e., pol III transcripts of B2) in mouse cell cultures and tissues were analyzed by the northern hybridization of total RNA (Figure 1). Autoradiography of B2 RNA revealed both a band corresponding to the primary 180-nt B2 transcript and a heterogeneous smear of 200–500-nt RNAs, which presumably represent polyadenylated B2 transcripts. The B2 RNA with a length of 120 nt has been previously documented [24,25]; it is plausible that this is the result of the processing of the primary transcript. The autoradiography further revealed a prominent 98-nt band, which is indicative of 4.5SI RNA, a B2-related gene [36].

The intensity of the 180-nt RNA band provides the most reliable estimate of the number of B2 pol III-transcripts. Trace amounts of B2 pol III-transcripts were found in the murine heart, spleen, lung, liver, and kidney; however, the brain and especially the testis contain large amounts of these transcripts. The number of B2 pol III transcripts observed in an NIH-3T3 cell culture (spontaneously immortalized fibroblasts) was approximately equivalent to that observed in the testis. However, the levels observed in three tumor cell cultures (4T1, L929, and CSML-100) were 2.0-, 6.5-, and 3.5-fold higher, respectively. B2 transcripts ranging from 200 to 500 nt are distinctly evident in these three cell lines, and are presumably attributable to variations in the length of the poly(A)-tails of the transcripts. The rightmost lane in Figure 1 corresponds to human HeLa cells that have been transfected with a copy of mouse B2, which contains an efficient pol III transcription terminator. In this case, it can be concluded that all B2 RNAs longer than 180 nt are the product of the polyadenylation of pol III-transcribed B2 transcripts, since transcription slippage over the effective terminator can be ruled out. It is noteworthy that the formation of long RNAs can exclusively be attributed to polyadenylation [19,27].

### 2.2. NGS of B2 Transcripts (Method 1)

**Figure 2:**
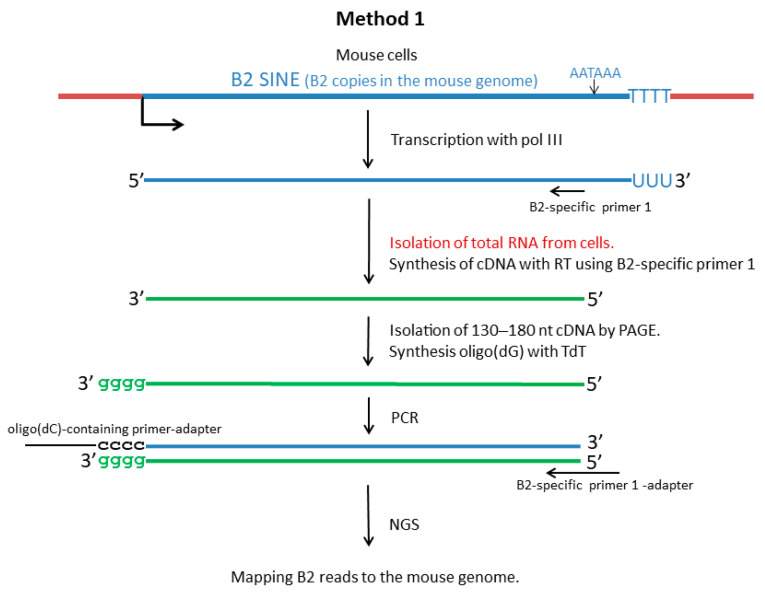
Schematic representation of the preparation of a DNA library complementary to pol III transcripts of B2 in mouse cells (Method 1). The B2 transcript and the complementary DNA are represented by blue and green lines, respectively. The following abbreviations are used: PAGE, polyacrylamide gel electrophoresis; RT, reverse transcriptase; TdT, terminal deoxynucleotidyl transferase. For other explanations, see text.

Karijolich et al. [37] proposed a method to produce cDNA libraries for NGS so as to analyze the diversity of transcripts read by pol III from B2 copies in a given cell line. For cDNA synthesis, they used the total cellular RNA and a primer that was complementary to a site near the 3′-end of B2 (positions 128–156) (Figure 2).

The cDNA synthesized on B2 transcripts read by pol III was approximately 160 nt in length. In contrast, the synthesis of cDNA containing B2 by pol II (primarily pre-mRNA) proceeded beyond the B2 sequence, resulting in cDNA molecules that were much longer than 160 nt. To exclude cDNA that was synthesized from mRNA and pre-mRNA molecules, a cDNA fraction of 130─180 nt was isolated, as this range approximately corresponds to the expected length of cDNA pol III-transcripts of B2 (Figure 2). Oligo(dG)-tails were synthesized at the 3′-ends of such cDNA, which allowed later PCR amplification of the library. The subsequent sequencing of the library provided a comprehensive set of B2 transcripts that were synthesized by pol III in a specific cell type. Given the presence of specific nucleotide substitutions and indels in the B2 copies, the majority of NGS-derived reads could be effectively mapped and assigned to a particular B2 copy in the mouse genome. Karijolich et al. [37] applied this method to characterize the B2 transcriptome of NIH-3T3 cells infected with MHV68 virus, which strongly induced B2 transcription.

**Figure 3:**
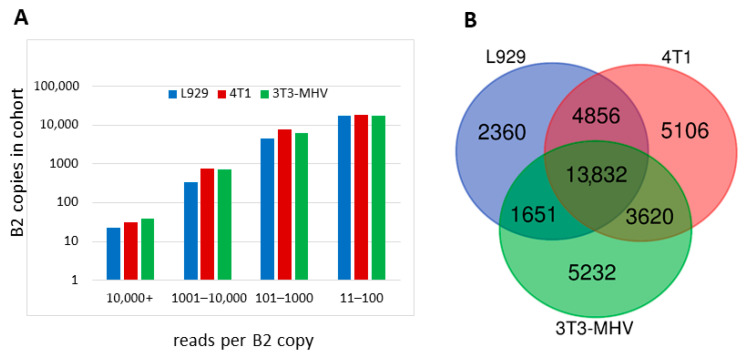
Analysis of B2 copies identified by Method 1 as pol III transcribed in L929, 4T1, and 3T3-MHV cells. (**A**) Distribution of B2 copies into four cohorts by the number of their reads per copy. (**B**) Venn diagram showing the number of B2 copies that are transcribed in one, two, or all three cell lines.

We used the same approach (Method 1 hereafter) to identify pol III-transcribed B2 copies and to evaluate their transcription in L929 and 4T1 cells. The selection of these cell lines was predicated on their elevated B2 RNA content, as illustrated in Figure 1. The nucleotide sequences obtained from these two cell lines were assigned to particular B2 copies in the mouse genome. Table S1 (worksheet 1) provides a comprehensive summary of the coordinates of all identified B2 copies and the number of their reads, along with analogous data from Karijolich et al. [37], who performed NGS on NIH-3T3 cells infected with the MHV68 virus (hereafter referred to as 3T3-MHV). To ensure the reliability of the results, B2 copies with fewer than 11 reads in a cell library were considered non-transcribed. In our quantitative analyses, the number of reads in the libraries was normalized to the lowest expression library (Table S2). The results obtained from the three cell lines demonstrated a consistent pattern: very few B2 copies were actively transcribed, while the majority of copies showed low transcription levels. For instance, approximately 30 B2 copies had more than 10,000 reads, whereas the number of B2 copies with 11–100 reads totaled around 18,000 (Figure 3A). Conversely, the transcriptional activity (number of reads) of the B2 copies could be similar in three or two cell lines or could vary by one, two, or even three orders of magnitude.

Finally, the number of B2 copies transcribed in three, two, or one cell lines was analyzed. The resulting data are summarized as a Venn diagram in Figure 3B. In the L929, 4T1, and 3T3-MHV cells, 22,699, 27,414, and 24,335 B2 copies were identified as having been transcribed by pol III, respectively. The data demonstrate that all three cell lines transcribed 13,832 B2 copies, constituting 50–61% of all copies that were identified as having been transcribed in a given cell line in these experiments. Transcription that was specific to a single cell line was observed for 10, 19, and 22% of the copies in L929, 4T1, and 3T3-MHV, respectively. The proportion of B2 copies that were transcribed in two of the three cell lines ranged from 7 to 21%. Therefore, although the majority of pol III-transcribed B2 copies were expressed in all three cell types, a significant proportion of them were limited to one or two cell types.

### 2.3. NGS of B2 Transcripts (Method 2)

#### 2.3.1. The Method Concept and Initial Analysis of NGS Data

**Figure 4:**
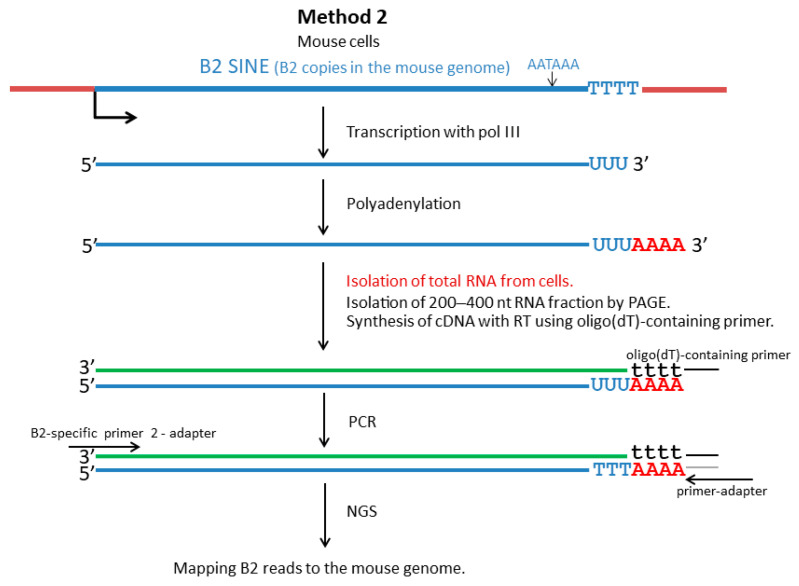
Schematic preparation of a DNA library complementary to low-molecular-weight RNA transcribed from B2 copies and polyadenylated in mouse cells (Method 2). This library encompasses the 3′-terminal sequences of B2 transcripts. AATAAA, polyadenylation signal; TTTT, pol III transcription terminator; AAAA, poly(A)-tail; PAGE, polyacrylamide gel electrophoresis; RT, reverse transcriptase. The B2 transcript and its complementary DNA are represented as blue and green lines, respectively. For other explanations, see text.

The approach described above does not provide information about the structure of the 3′-terminal region of B2 transcripts. Our research focused on the tail sequences of B2 transcripts, taking into account transcription termination and polyadenylation. We used an original approach to obtaining cDNA libraries that allows for the sequencing of almost full-length B2 transcripts, including their tail. Figure 4 illustrates this method, Method 2. To eliminate or at least minimize the contamination of pol III-transcribed B2 RNA by pol II-generated B2-containing transcripts (mainly pre-mRNA), a 200–400 nt fraction of cellular RNA was isolated by polyacrylamide gel electrophoresis (PAGE) (Figure 1).

**Figure 5:**
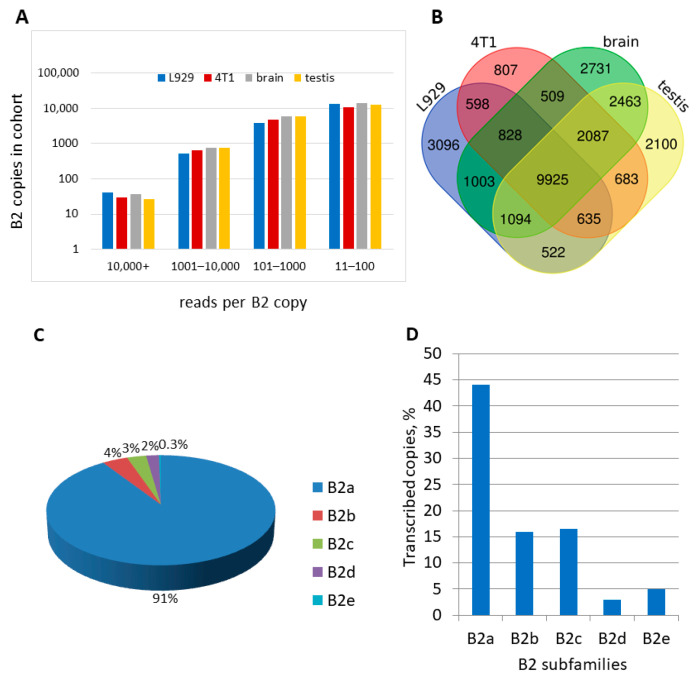
Analysis of B2 copies identified by Method 2 as transcribed in L929 and 4T1 cell lines and mouse tissues (brain and testes). (**A**) Distribution of B2 copies into four cohorts by the number of reads per copy. (**B**) Venn diagram indicating the number of B2 copies that were transcribed in one, two, three, or four cell types. (**C**) Proportion of copies belonging to each of the five subfamilies (a, b, c, d, and e) among all B2 copies transcribed. (**D**) Proportion of transcribed copies in B2 subfamilies. The number of transcribed copies was summarized across all four cell types; the following estimates of the number of B2a, B2b, B2c, B2d, and B2e copies in the mouse genome were used in the calculations: 59.0, 7.2, 5.0, 18.7, and 1.5 thousand, respectively [19].

This RNA fraction should be free of much longer mRNA and pre-mRNA molecules. The length range (200–400 nt) was chosen considering that the length of B2 is ~180 nt and that polyadenylation should increase the length of B2 RNA to 200–500 nt (predominant B2 RNA length in HeLa cells transfected with a B2-containing plasmid; see right lane in Figure 1 and also [27]). An oligo(dT)-containing primer was used to synthesize the first strand of the cDNA. The double-stranded cDNA was amplified using, first, an adapter primer that shared a common sequence with the oligo(dT)-containing primer (Figure 4) and, second, a primer corresponding to the first 25 nucleotides of the B2 sequence. Method 2 was used to obtain libraries for the L929 and 4T1 cell lines as well as for mouse testis and brain. The NGS reads of the libraries were mapped to the mouse genome. Table S1 (worksheet 2) summarizes the coordinates of all identified B2 copies and the number of reads of each. For further quantitative analyses, only B2 copies corresponding to at least 11 reads in a given library were considered as transcribed. The number of reads in the libraries was normalized to the lowest expression library (Table S2). Although the cellular level of the B2 RNA transcribed by pol III was significantly lower in tissues than in cell lines (Figure 1), the number of transcribed genomic copies of B2 was slightly higher in the brain (20,639) and testis (19,508) than in the L929 (17,700) and 4T1 (16,053) cells. As in Method 1, analysis of these four libraries showed that only a few B2 copies were actively transcribed (Figure 5A). For example, only 29–40 B2 copies provided more than 10,000 reads per copy, whereas 10,657–13,980 copies provided 11–100 reads per copy, depending on the cell line.

Next, we analyzed the overlap of the sets of transcribed B2 copies in the cell lines that were studied (Figure 5B). The transcribed copies in all four cell lines accounted for 56, 62, 48, and 51% of the B2 copies that were expressed in L929 cells, 4T1 cells, the brain, and the testis, respectively. The proportion of B2 copies transcribed in a single cell line varied considerably: 5% (4T1), 11% (testis), 13% (brain), and 17% (L929). The copies that were simultaneously transcribed in brain and testis but not in other cells were markedly more abundant (12%) than the copies that were transcribed simultaneously in other cell pairs (2.5–3.7%). Thus, about half of the B2 copies transcribed by pol III are expressed in all four cell types, but copies that are transcribed specifically in one or two cell types are also abundant.

Method 1 detects transcripts of the B2a subfamily only, since the primer used for cDNA synthesis is specific to the 3′-end of B2a and not for other subfamilies (Figure S1). In contrast, for the libraries generated by Method 2, we used a primer that was specific to the 5′-terminal region of B2, which is common to all B2 subfamilies (Figure S1). Thus, the libraries obtained by Method 2 contained B2 copies from different subfamilies: 91, 4, 3, 2, and 0.3% for B2a, B2b, B2c, B2d, and B2e, respectively (Figure 5C). The significant prevalence of B2a over other subfamilies is related not only to the large number of B2a copies in the genome but also to the relatively recent emergence of this subfamily (hence, promoters of B2a copies are less prone to deleterious mutations). Evaluation of the proportion of transcribed copies within each subfamily (Figure 5D) shows that B2a is much more frequently transcribed (44%) than B2b and B2c (16%), and even more so than B2d and B2e (3% and 5%). Mutations accumulated over time are probably responsible for the low transcriptional competence of these oldest subfamilies.

#### 2.3.2. Evidence for the Polyadenylation Capacity of Pol III Transcripts of B2 Copies in Mouse Cells

**Figure 6:**
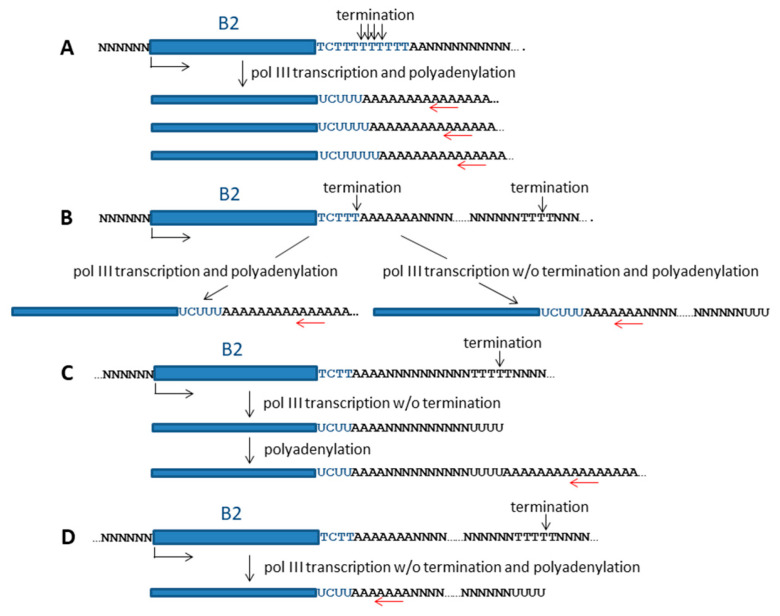
Four categories of B2 copies with or without functional pol III transcription terminators. In addition to genomic copies of B2, transcripts with the poly(A)-tails acquired after polyadenylation are shown. The main part of the B2 sequence is shown as a blue rectangle, and the nucleotide sequence of the transcription terminator is shown in blue font. Vertical arrows indicate the putative transcription termination points, while red horizontal arrows denote the oligo(dT)-containing primer used for cDNA synthesis. (**A**). B2 with an efficient long terminator. (**B**) B2 with a minimal TCTTT terminator and another terminator in the far downstream sequence. (**C**) B2 with a rudimentary terminator (TCTT) and a nearby full-length terminator. (**D**). B2 with a rudimentary terminator and a distant (>60 bp) functional terminator.

The objective of this study was to obtain direct, unambiguous evidence for the polyadenylation of pol III transcripts of B2 in mouse cells. To this end, the 3′-terminal regions of the transcripts of a variety of B2 copies that were sequenced by Method 2 were analyzed. It was determined that B2 copies with a long transcription terminator are best suited to demonstrate the polyadenylation of pol III-generated transcripts. The transcription of these copies by pol III terminates at distinct Ts in the terminator, and only polyadenylated B2 transcripts can serve as templates for cDNA synthesis and subsequent NGS (Figure 6A).

The evaluation of sequenced cDNA libraries from L929 cells (Table 1) and testis (Table 2) identified 32 B2 copies with terminators of varying lengths and structures that merit further investigation. Subsequent examination of the reads corresponding to these B2 copies enabled the determination of the frequency of transcription termination at each of their terminator positions.

**Figure 7:**
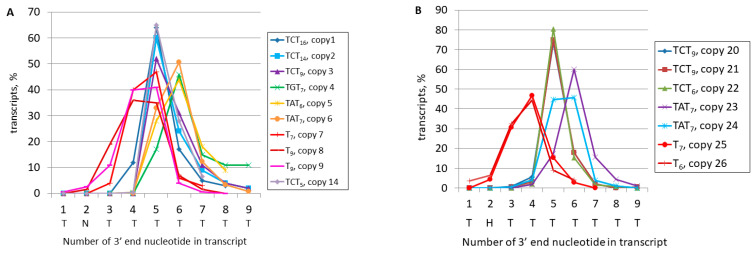
Transcriptional arrest at long terminators of individual copies of B2. The terminator nucleotide positions are delineated on the X-axis, and the nucleotides are specified below (H = T, C, or A). The graphs depict the percentage of transcripts that terminate at each of the terminator nucleotides in individual B2 copies, whose numbers and terminators are indicated on the right. The data for L929 cells (**A**) and testis (**B**) are presented.

As illustrated in Figure 7, these data pertain to transcripts of genomic B2 copies with long (6–18 nt) terminators (copies 1–9, 14, and 20–26). The transcription consistently halted within these terminators in both the L929 cells (Figure 7A) and testis (Figure 7B). In terminators that comprised exclusively T residues, the transcription predominantly terminated at positions 4 and 5 in L929 and positions 3 and 4 in the testis. The termination peaked at the fifth position for TCT_6–18_ and at the sixth position for TAT_6–7_ and TGT_7_. The termination pattern of the B2 copies observed in this study indicates their transcription by pol III rather than pol II. The detection of a significant number of the obtained cDNA library reads with the terminators that are shorter than those in their genomic copies unambiguously confirms the polyadenylation of pol III transcripts of B2 in mouse cells.

**Figure 8:**
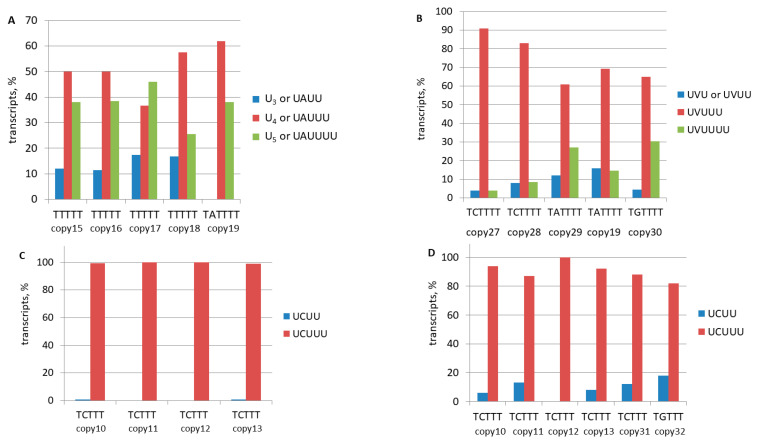
Transcription termination at medium (**A**,**B**) and short (**C**,**D**) terminators of individual copies of B2. The diagrams provide a quantitative representation of the proportion of transcripts that terminate at a specific terminator nucleotide. The remainders of the terminators are depicted on the right (V = C, A, or G). The B2 copy numbers and their respective terminator sequences are specified below. The data presented here correspond to L929 cells (**A**,**C**) and testis (**B**,**D**).

In a similar manner, the polyadenylation of pol III transcripts of B2 copies with medium-length terminators can be demonstrated for T_5_ (copies 15–18), TAT_4_ (copies 19, 29), TCT_4_ (copies 27, 28), and TGT_4_ (copy 30). Significantly more than half of the transcripts had a truncated terminator, relative to their genomic copies, followed by a poly(A)-tail, thereby suggesting that pol III transcription termination was followed by polyadenylation (Figure 8A,B).

However, confirming the polyadenylation of transcripts of B2 copies with short terminators, such as TCTTT or TGTTT, is more challenging. In L929 cells, the transcription of such terminator sequences did not show significant shortening (Figure 8C). However, a single-nucleotide truncation was observed in 6–18% of the transcripts in five out of six B2 copies that were tested in the testis (Figure 8D). Such shortened terminator sequences favor pol III transcription as well as the further polyadenylation of transcripts. Typically, genomic copies of B2 contain at least a small (5–7 nt) A-tail, which is located immediately downstream of the terminator. The detection of a significant proportion of transcripts with untruncated TSTTT terminator sequences in the cDNA libraries can be interpreted in two ways (Figure 6B). First, it can be interpreted that pol III transcription ceased at the last nucleotide of the terminator, after which the resulting RNA was polyadenylated. Alternatively, pol III may have failed to stop at the terminator, resulting in the transcription of the A-tail and the downstream genomic DNA until it was halted at a random terminator. In this scenario, polyadenylation did not occur and cDNA synthesis could be primed from the A-tail encoded in the genome.

The mouse genome contains rare B2 copies with a TCTTT terminator that lacks an A-tail. Four of these copies were included in the analysis (copies 11, 12, 31, and 32; the latter has a TGTTT terminator). In the majority of these cases, pol III terminated the transcription at the last terminator nucleotide, after which polyadenylation of the resulting B2 RNA occurred, as indicated by the nucleotide sequences of the reads corresponding to copies 31 and 32 (Figures S2 and S3). In addition, these figures demonstrate that pol III can skip the B2 terminator and stop at a random downstream terminator. These transcripts constituted 11% and 15% of the total transcripts for these two B2 copies, respectively. The terminators in the 3′-flanking sequences were 55 bp (copy 31) and 24 bp (copy 32) downstream of the inherent terminator. Intriguingly, this increased distance from the PAS did not hinder polyadenylation, although it could diminish its efficiency.

Consequently, this section demonstrates that pol III transcripts of B2 containing both long and short terminators undergo polyadenylation in L929 cells and the testis.

#### 2.3.3. Comparison of B2 Transcription Termination Profiles in Different Murine Cells

The data presented in Figure 7A,B and Figure 8C,D allowed us to propose that transcription termination can be more efficient in the testis than in L929 cells (where transcription almost never stopped at the fourth nucleotide of the TCTTT terminator). To validate this observation, eight B2 copies with long terminators (copies 2, 7, 9, 19, 20, 21, 22, and 24) and with a reasonable number of reads in both the L929 and testis libraries were selected for testing. The relative numbers of transcripts (reads) were plotted against the nucleotide position in the terminator where RNA synthesis was stopped for each copy (Figure S4). The analysis of these plots revealed a greater number of termination events at proximal terminator nucleotides in the testis than in the L929 cells, where termination occurred at a wider nucleotide range. Thus, the same B2 terminators halt pol III transcription slightly earlier and, therefore, more efficiently in the testis than in L929 cells.

A selection of six additional B2 copies from the mouse genome that featured moderately long terminators and active transcription, as indicated by the number of reads in the four cell types (L929, 4T1, brain, and testis)—was analyzed. The frequency of termination at a specific nucleotide of the terminator is illustrated in Figure S5. Four copies (A, B, C, and F) exhibited a more extensive distribution of transcripts in L929 compared to the other three cell types. Overall, the termination efficiency of pol III transcription exhibited an increasing trend from L929 to 4T1 to the brain to the testis. The observed variations in termination could be ascribed to pol III (e.g., subunit modifications) and/or to the chromatin context of B2 copies across the diverse cell types.

#### 2.3.4. Analysis of the Number and Types of B2 Copies Whose Pol III Transcripts Can Be Polyadenylated in Mouse Cells

As demonstrated above, pol III transcripts from B2 copies that contain active transcription terminators are polyadenylated in mouse cells. However, it remains to be elucidated whether all B2 copies that were identified by the cDNA library sequencing produce transcripts that undergo polyadenylation. Transcripts of a significant number of non-polyadenylated B2 copies could potentially serve as templates for cDNA synthesis (Method 2) after priming on poly(A)-tails encoded in the genome. Such B2 copies are predicted to lack a functional terminator but possess a poly(A)-tail and a pol III transcription terminator within 220 bp of the 3′-flanking sequence, given the use of RNAs shorter than 400 nt for cDNA synthesis and the 180 nt length of B2s. Subsequent analysis of the NGS data (see below) revealed that nearly all B2 copies that were devoid of a functional transcription terminator possessed an A-rich tail and at least one effective terminator within 220 bp of their 3′-flanking sequence.

Consequently, three samples of B2 copies were obtained. The list of B2 copies identified in the four cDNA libraries (Method 2) was sorted by number of total reads in descending order (Table S1, worksheet 2) and copies 1–100 (Table S3), 501–600 (Table S4), and 1051–1150 (Table S5) were analyzed manually with particular attention paid to the transcription terminators and the localization of the copies within (in introns or exons) protein-coding genes or outside them. The pol III transcripts of B2 copies with fairly long terminators (TCT_≥4_, TAT_≥4_, T_≥4_) were found to undergo polyadenylation (Figure 6A). Such copies, designated as category A (indicated by a green “PA” in Tables S3–S5), account for 36% of all B2 copies. The reads were manually analyzed for many of the category A copies, and their pol III transcription was confirmed by a significant shortening of the terminator sequence relative to their genomic copies.

The copies in category B have a short and weak TCTTT terminator that halts the synthesis of only 50% of transcripts, as indicated elsewhere [19,38]. It is challenging to confirm the polyadenylation of most B2 copies with this terminator using cDNA sequence analysis (however, there are rare exceptions, as evidenced by copies 11, 12, 31, and 32 in Table 1 and Table 2, which lack a poly(A)-tail encoded in the genome). It appears that half of the B2 transcripts with the TCTTT terminator terminate with this sequence, subsequently undergoing polyadenylation. Conversely, the transcription of the other half of the transcripts terminates at random downstream terminators. The cDNA of these transcripts is indistinguishable from that of polyadenylated transcripts of the same B2 copies, as both are primed from the poly(A) that is located immediately downstream of the terminator (Figure 6B). Copies of this category are marked with a yellow “PA” in Tables S3–S5, representing approximately 18% of the analyzed samples.

Category C includes B2 copies whose pol III-generated transcripts are also likely polyadenylated. In the event that a B2 copy possesses no functional terminator but an effective terminator is located in the nearest 3′-flanking sequence, the resulting transcript is competent for polyadenylation (Figure 6C), as substantiated by the analysis of reads from a number of such copies. The distance between the rudimentary terminator and a random downstream terminator ranged from 28 to 97 bp (50 bp on average) among the nine B2 examples shown in Figure S6. This distance between the polyadenylation signal and the 3′-end of the transcript did not prevent poly(A) synthesis. However, the efficiency of polyadenylation decreased with the distance between the B2 and the external terminator. It is noteworthy that only those copies that fell within a 60-base pair distance from the external terminator were included in this category. The category C copies, which accounted for an average of 15% in the samples that were analyzed, are marked with a brown “PA” in Tables S3–S5.

Finally, category D includes B2 copies whose pol III transcripts do not undergo polyadenylation. These B2 copies have a rudimentary transcription terminator (e.g., TCT_<3_ or T_<4_) and an A-rich tail, and their 3′-flanking sequence contains a full terminator located within 60–220 bp of the B2 end. The transcription from these B2 copies (Figure 6D) terminates at such a downstream terminator, and their cDNA synthesis is primed at the genomic A-rich tail sequence of B2. Our observations indicate that six adenosines in a row are sufficient to initiate cDNA synthesis. As the category D transcripts are not polyadenylated, they have no “PA” label in Tables S3–S5. On average, these copies constituted 31% of the samples that were examined.

Based on the cumulative data presented in Tables S3–S5, we estimated the proportion of transcripts (reads) from B2 copies that belong to each category (Figure S7). It was found that the B2 reads of category A constitute about 43% of the transcripts in the brain and testis, whereas, in cell cultures (L929 and 4T1), their proportions are significantly lower—22% and 24%, respectively. In contrast, the B2 reads of category B are more abundant in cell cultures (35–38%) than in tissues (about 24%). The reads from category C account for a roughly similar proportion (12–15%) in all four cellular samples. The same applies to the reads of category D (20–25%). The results of this analysis, based on a limited dataset, allow us to preliminarily conclude that the relative levels of transcripts from B2 copies of categories A and B may differ significantly between cell cultures and mouse tissues.

The analysis revealed that approximately 44% of the B2 copies in the samples are located outside pol II-transcribed genes, which provides strong evidence suggesting that these copies are not transcribed by pol II (Tables S3–S5). Furthermore, there is no evidence to support the hypothesis that pol II transcribes B2 copies which are located in introns and oriented in the opposite direction of the gene transcription (these copies constituted 32% of all copies in the samples). The intronic copies that are oriented in the same direction as the gene accounted for approximately 18% of the samples, suggesting that these copies are transcribed by pol II as part of pre-mRNA molecules. However, it is plausible that their cDNAs are absent from our cDNA libraries due to the RNA template size limit (200–400 nt). A thorough analysis of numerous B2 copies with sufficiently long terminators consistently revealed the presence of the pol III transcriptional signature, which is characterized by shortened terminator sequences. Consequently, it can be concluded that the intronic B2 copies present in the cDNA libraries are transcribed by pol III. In contrast, approximately 6% of the copies were found within exons, specifically in the untranslated regions of the last exon. In instances that involved a unidirectional B2 copy and the gene, the B2 terminator in the reads often remained untruncated, indicating pol II transcription. The presence of such cDNAs in our libraries can be attributed to slight contamination with mRNA degradation products. Given the rarity of such exonic B2 copies in our cDNA libraries, they do not significantly distort our conclusions.

## 3. Discussion

### 3.1. Comparison of Approaches to Sequencing B2 Transcripts

As is the case with other abundant SINEs, a significant proportion of B2 copies are found within genes, predominantly in their introns. Consequently, numerous B2 copies are transcribed by pol II as part of pre-mRNA molecules and, likely, long non-coding RNAs. Northern hybridization has revealed numerous B2 sequences among long (≥1000 nt) nuclear RNAs [23]. The abundance of B2-containing RNAs that are synthesized by pol II in murine cells substantially complicates the NGS-based identification of pol III transcripts. In addressing this challenge, two approaches were employed in this study. The first approach was proposed by Karijolich et al. [37]. This method, termed SINE-seq, utilizes a primer that is specific to a B2 sequence located in the 3′ half of SINE. The cDNA synthesis initiated from this primer halts at the 5′-end of B2 transcripts that have been synthesized by pol III or extends further on pol II B2-containing transcripts. The latter appear to be longer and were eliminated during the preparation of libraries for NGS (Figure 2). The second method commences with the processing of a 200─400 nt fraction of cellular RNA, thereby ensuring the removal of longer pre-mRNA and mRNA molecules. The first cDNA strand is initiated from an oligo(dT)-containing primer which is designed to bind to the poly(A)-tails of B2 transcripts synthesized by pol III. Subsequent cDNA libraries undergo amplification through second-strand synthesis from a primer that matches the 5′-end sequence of B2 (Figure 4). Method 2 has been developed for the purpose of obtaining the nucleotide sequence information of B2 transcripts, including their 3′-end sites. This is critical for studying the transcription termination of SINE copies and the polyadenylation of the resulting transcripts.

The NGS of the libraries obtained by each method yielded 7.7–15 million reads of B2 sequences (Table S2), of which 35–40% (method 1) and 60–72% (method 2) were mapped unambiguously, i.e., to a single B2 copy in the mouse genome. The higher rate observed in Method 2 can be attributed to the longer reads and the higher variability of the B2 3′-terminal sequences, which are provided specifically by this method. Method 1 demonstrated 23,000–27,000 distinct copies of B2 that were definitively transcribed in each cell line (L929 and 4T1), with approximately three-fourths of these copies being transcribed in both lines. Method 2 also revealed the transcription of 16,000–17,700 different copies of B2 in L929 and 4T1, and 67–75% of these copies were transcribed in both lines. Method 2 revealed a slightly wider range of transcribed B2 copies in the brain and testis, with 20.6 and 19.5 thousand copies, respectively. Furthermore, from 75% to 80% of these copies were transcribed in both organs, indicating a significant degree of transcriptional activity of the same B2 copies in two cell lines or in two organs.

The sets of transcribed B2 copies detected by Methods 1 and 2 in the same cell type differed significantly. In the case of the L929 cells, only 43–55% of the copies detected by one method were also identified as transcribed by the other method; similar values were obtained for the 4T1 cells. In addition, the number of reads detected by the two methods that corresponded to a single copy of B2 could vary widely. The analysis of these copies revealed that the observed low number of reads was attributed to sequence mismatches between the primer and the B2 copies. Our observations suggest that even three nucleotide mismatches in the 5′ sequence of the primer had little effect on the efficiency of cDNA synthesis; however, a single substitution within three nucleotides from the primer 3′-end sufficed to prevent cDNA synthesis (or to highly reduce its rate). Method 2 has been found to be capable of detecting transcripts that belong to subfamilies B2a, B2b, B2c, B2d, and B2e, while Method 1 has been found to be limited to B2a, as the primer used in Method 1 is specific to the B2a consensus but not to the sequences of the other four subfamilies (Figure S1). Furthermore, there are copies of B2a that possess long nucleotide substitutions or deletions within the region that should be paired with primer 1, which hinders their detection by Method 1 (though not by Method 2). Such cases are not uncommon, as even substantial modifications within the primer 1 matching sequence do not impede the transcription of such copies. Conversely, there may exist B2 copies whose transcripts are not detected by Method 2, due to the absence of a poly(A)-tail being encoded in the genome or acquired by polyadenylation.

Given that approximately half of the B2 copies that were identified as transcribed by one method were not detected by the other method, the estimated number of transcribed B2 copies should be increased by about 1.5-fold. It is plausible that neither of the two B2-specific primers utilized in these methods will correspond to the transcripts of certain B2 copies that are critical for priming such copies, due to mismatches, resulting in their absence from the obtained libraries. Furthermore, instances of the mapping of the same reads to two or more B2 copies were excluded, leading to further underestimation of the number of transcribed B2 copies. Consequently, it can be deduced that the transcription of B2 copies by pol III is considerably more frequent than anticipated. Based on the observations made herein, it is estimated that more than half of all B2a copies are capable of pol III transcription in the studied cells.

There have been NGS reports of SINE B2 transcripts that were generated by pol III, which was ascertained using methods different from those in this work. Linker and colleagues [39] developed a strategy that they termed BonaFide-TEseq. This strategy involves the addition of a primer (template-switch oligo) when the reverse transcriptase reaches the end of the template RNA molecule, which thereby tags the 5′-end of a transcript. However, the reads obtained by this method are too short to assemble complete transcript sequences and map them to individual B2 copies. Conversely, this approach enables the assessment of alterations in the transcription levels of B2 or other transposable elements from their innate promoters. A similar approach has been employed recently by Oomen and colleagues [40]. Ichiyanagi and colleagues [21] proposed a technique they that they designated Medium Length RNA-Seq. The authors began by removing long cellular RNAs from the testis, then performed the nonspecific synthesis of full-length cDNAs, the isolation of 115–255 bp double-stranded cDNAs, and NGS of the resulting library. As all RNA species falling within this length range were sequenced, these authors obtained only 250,000 reads corresponding to B2 transcripts (30–70 times less than the number of reads obtained in our study). Mapping these reads to the mouse genome enabled the identification of 8088 copies of pol III-transcribed B2. Furthermore, this work established the distribution of reads, and thus transcripts, across B2 subfamilies. However, the transcription termination of B2 copies and the polyadenylation of the resulting RNAs were not addressed in these studies.

### 3.2. Termination of Transcription and Polyadenylation of B2 Transcripts

The most significant results of this work were obtained by comparing the nucleotide sequences of genomic copies of B2 with their corresponding reads from the libraries prepared by Method 2. The focus was placed on the 3′-terminal regions of B2, which contain an effective terminator (usually T_≥4_ or TCT_≥4_, less frequently TAT_≥4_ or TGT_≥4_) in approximately 40% of the examined copies. The reads corresponding to these copies, referred to as category A, exhibited a truncation of the terminator sequence, indicating their transcription by pol III. The termination of transcription occurred at various frequencies at the terminator nucleotide positions. In terminators consisting of T residues only, the maximum efficiency was observed at nucleotide positions 3–4: TTTTT; in the case of TCT_≥4_, the transcripts most often ended at the fifth nucleotide: TCTTTT (Figure 7). The analysis of the reads revealed that the shortened terminator sequence was invariably succeeded by a poly(A)-tail, thereby providing unequivocal evidence for the polyadenylation of pol III-transcripts of B2 in mouse cells (it is noteworthy that B2 transcripts devoid of poly(A)-tails could not be represented in the Method 2 libraries).

In contrast to the B2 copies categorized as category A, category B copies have moderately to marginally effective TCT_3_, TAT_3_, or TGT_3_ terminators that stop transcription in only 50, 40, and 20% of cases, respectively [19,38]. These terminators are rarely truncated during transcription (Figure 8C,D), which complicates the confirmation of their pol III transcription. However, the analysis of reads from the rare category B copies that lack a genomic A-rich sequence at the SINE 3′-end clearly demonstrates their pol III transcription followed by the polyadenylation of the resulting RNA (Figures S2 and S3). Category C includes B2 copies that carry only a rudimentary terminator (T_<4_ or TCT_<3_) but contain a full terminator in their relatively near (≤60 bp) 3′-flanking sequence. It appeared that transcripts terminating at such downstream terminators were also capable of polyadenylation, although with conceivably lower efficiency (Figure S6). Finally, the copies in category D, which accounts for approximately 30% of the observed copies, are arranged similarly to those in category C but lack terminators in the near downstream sequences. Their transcripts are not polyadenylated but are still represented in the Method 2 libraries since cDNA synthesis is primed at the A-rich tails in the genomic copies (Figure 6D).

A series of experiments were conducted previously in which HeLa cells were transiently transfected with category A copies of B2 that had been modified through the deletion or substitution of specific nucleotides. These experiments yielded the conclusion that the process of the polyadenylation of pol III transcripts necessitates the presence of the PAS (AAUAAA), a β-signal located downstream from the B box of the pol III promoter, and a τ-signal located upstream from the PAS [27,29]. In the same system, the knockdown of proteins facilitated the identification of protein factors that are required for the polyadenylation of B2 transcripts. These factors include the CFIm, which interacts with τ-signaling in RNA; the mammalian polyadenylation specificity factor (mPSF), which consists of four subunits and recognizes PAS; and the canonical poly(A)-polymerase (PAP) [29,30]. It has been established that these factors are involved in the 3′-processing of pre-mRNAs that encompass RNA cleavage at the polyadenylation site and subsequent poly(A) synthesis [41,42]. The CFIm has been demonstrated to promote the binding of mPSF to PAS, and mPSF has been shown to recruit PAP, thereby triggering polyadenylation in many pre-mRNAs [43]. It has been observed that analogous interactions between the protein factors as well as the B2 transcript result in its polyadenylation (it should be noted that a protein that recognizes the β-signal and likely contributes to the binding of mPSF to PAS in B2 RNA has yet to be identified). Conversely, the mammalian cleavage factor (mCF), a critical component of the 3′-processing of pre-mRNAs that cleaves these RNAs at the polyadenylation site [42], was found to be non-involved in the polyadenylation of B2 transcripts [30]. This outcome is consistent with the notion that the transcription termination of pol III depends on a distinct mechanism, in contrast to pol II, which does not necessitate RNA cleavage.

The polyadenylation scheme under consideration was developed on a category A copy of B2 and its applicability to category B copies is a matter of plausibility. Within the framework of this polyadenylation scheme, consideration should be given to category C copies. The transcription of a B2 copy lacking a functional terminator, which stops at a random downstream terminator, can proceed to polyadenylation. This phenomenon has been observed to occur consistently for downstream terminator distances of up to 60 bp from the B2 rudimentary terminator; however, greater distances of 75, 82, and 97 bp have been observed to potentially result in polyadenylation (as depicted in Figure S6B,D,H). In mRNA, the typical distance between the PAS and the polyadenylation site is 15–25 nt, with extremes of 10 and 40 nt being observed [44]. However, the distance between the PAS and the downstream site where polyadenylation starts in category C transcripts of B2 can considerably exceed the limit of 40 nt. The protein complex responsible for the polyadenylation of B2 transcripts appears to be more compact and less complex compared to the complex involved in the 3′ processing of pre-mRNA. The latter complex, in addition to mPSF, comprises mCF and other factors, all of which are associated with the largest pol II subunit [41,42]. Conversely, the complex involved in the polyadenylation of pol III transcripts of B2 (CFm/mPSF/PAP) does not appear to be associated with pol III, as this polymerase lacks subunits that are capable of binding these factors [29,30]. The formation of such a complex bound to the PAS in the B2 sequence could potentially recruit a 3′-end of the transcript that is quite distant from the PAS and initiate polyadenylation. However, there is evidence to suggest that this distance significantly impacts the polyadenylation efficiency. Previous studies on the transfection of HeLa cells with B2 copy-based constructs have shown that an increase in the distance between the PAS and the terminator by 25 bp resulted in a 2.5- to 3-fold reduction in the proportion of polyadenylated B2 transcripts [45]. The polyadenylation of B2 transcripts exhibits a degree of sensitivity to the distance between the PAS and the downstream terminator, although this effect is less pronounced in murine cells than in foreign HeLa cells. This discrepancy can be attributed to the polyadenylation process being facilitated by heterologous protein factors in HeLa cells. Furthermore, polyadenylation is likely to occur at B2 transcription sites, indicating that it takes place in distinct environments: on chromosomes in mouse cells and in the nucleoplasm in transfected HeLa cells.

This study demonstrates that murine cells (L929, 4T1, testis, and brain) generate B2 transcripts that exceed the SINE template in length. The prevalence of these 200–400 nt transcripts has been observed to be from two to three times higher than that of the primary 180 nt B2 transcript (Figure 1). Two distinct mechanisms underlie the elongation of B2 transcripts. The first is polyadenylation, which is defined as the post-transcriptional formation of heterogeneous lengths of poly(A) at the 3′-ends of transcripts synthesized by pol III. The second mechanism is the transcription of B2 copies that lack effective pol III terminators, which continues at the 3′-flanking sequence and stops upon reaching a random terminator in genomic DNA. As previously mentioned in the Introduction, the polyadenylation of B2 transcripts as well as that of other T^+^ SINEs has been shown to significantly increase the lifetime of these RNAs in cells [24,31,45]. Moreover, poly(A)-tails arising from T^+^ SINE transcripts as a result of polyadenylation were proposed to be critical for the retrotransposition of such SINEs [19]. A substantial body of research has demonstrated that B2 transcripts synthesized by pol III have the capacity to regulate the transcription of protein-coding genes by interacting with specific chromosomal regions [12], RNA polymerase II [7,13], or glucocorticoid receptor [14]. These researchers hypothesize that B2 transcripts precisely match the SINE sequence, disregarding numerous polyadenylated B2 RNAs and transcripts that include 3′-flanking sequences. It is plausible that poly(A)-tails in B2 RNAs and 3′-flanking sequences in B2 transcripts may influence or even participate in the aforementioned regulation. Further research is necessary to expand our knowledge of the significance of non-coding RNAs, as represented by the diverse B2 transcripts.

## 4. Materials and Methods

### 4.1. Cell Cultures and Mouse Organs

The following cell lines were used in this study. L929 (ATCC CCL-1) is a fibroblast-like cell line derived from the subcutaneous connective tissue of a male C3H/An mouse. 4T1 (ATCC CRL-2539) is a breast cancer cell line derived from the mammary gland tissue of a mouse BALB/c strain [46]. NIH-3T3 (ATCC CRL-1658) is a fibroblast cell line that was isolated from a mouse NIH/Swiss embryo. CSML-100 is a cell line derived from mammary adenocarcinoma in a mouse of A/Sn strain [47,48]. The cells were grown to a monolayer in 75 cm^2^ flasks using DMEM with 10% fetal bovine serum.

Tissue samples collected from C57Bl/6 male mice stored in liquid nitrogen were provided by the Animal Facility of the Center for Precision Genome Editing and Genetic Technologies for Biomedicine, Engelhardt Institute of Molecular Biology, Russian Academy of Sciences (Moscow, Russia).

### 4.2. RNA Isolation and Northern-Blot Analysis

Cells were lysed in culture flasks with 6 mL of solution D (4 M guanidinium thiocyanate, 25 mM sodium citrate, pH 7.0, 0.5% Sarkosyl) and total cellular RNA was isolated according to the Chomczynski-Sacchi’s method [49]. Mouse organs were ground in a mortar with liquid nitrogen. The resulting powder was then mixed with solution D at a ratio of 1:10 (*w*:*v*). RNA was subsequently isolated by the same method [49]. A portion of purified RNA (200 μg) was incubated with 80 u of RNase-free DNase and 20 u of RiboLock RNase Inhibitor (both from ThermoFisher Scientific, Waltham, MA, USA) to remove any potential DNA contamination. The obtained RNA samples were subsequently utilized for northern blot analysis and the preparation of cDNA libraries. For northern blot analysis, RNA samples (10 μg) isolated from various cell cultures and mouse organs were resolved by electrophoresis in a 6% polyacrylamide gel with 8M urea (150 × 150 × 1 mm). Subsequently, RNA was transferred from the gel onto a nylon membrane (GVS, Bologna, Italy) by semidry electroblotting at 3 V for 2.5 h. A hybridization probe corresponding to the 5′-half of the SINE B2 was prepared and labeled with α[^32^P] dATP by PCR as described previously [27]. For RNA hybridization on the membrane, the membrane was incubated overnight in a mixture containing 50% formamide, 5 × Denhardt solution, 4 × SSC, 1% SDS, 0.1 mg/mL salmon sperm DNA, and the labeled B2 probe at 42 °C. Subsequent washes were performed in 0.1% SSC and 0.1% SDS at 42 °C for 1 h. The hybridization image was obtained by scanning the membrane with a Phosphorimager (Image Analyzer Typhoon FLA 9000; GE Healthcare Bio-sciences, Uppsala, Sweden). The OptiQuant 3.0 program was used for the quantitative analysis of the image.

### 4.3. Preparation of cDNA Libraries and NGS

Method 1 (Figure 2): This approach, termed SINE-seq, was developed by Karijolich and colleagues [37]. To prepare B2 cDNA libraries, we followed the published protocol with a modification: B2-specific primer used for cDNA synthesis was extended by six nucleotides (Table S6). The B2 cDNA library and a PhiX phage library were then mixed at a 5–1 ratio and subjected to sequencing on an Illumina MiSeq instrument at Evrogen (Moscow, Russia). The resulting single-end MiSeq reads were 175 bp in length.

Method 2 (Figure 4): This approach was developed by us and is described here in detail. Cellular RNA (100–200 μg) was separated by electrophoresis in a 6% polyacrylamide gel containing 8M urea. To isolate low-molecular-weight RNA, the gel region corresponding to 200–400 nt was excised and placed in a 50 mL tube containing 6.5 mL of buffer (20 mM Tris–HCl, 300 mM NaCl, 2 mM EDTA, 0.2% SDS). The tube was flash frozen in liquid nitrogen and then incubated overnight at room temperature. The buffer containing the RNA was extracted with phenol-chloroform and the RNA was precipitated with ethanol in the presence of linear acrylamide (20 μg). A quarter of the isolated RNA was mixed with 12 pmol of oligo(dT)-containing primer (Table S7), incubated at 80 °C for 3 min then at 42 °C for 10 min, and finally cooled on ice. The synthesis of the first cDNA strand was then carried out in a mixture containing RNA, the primer, First Strand buffer, dNTPs (0.5 mM of each), and 100 u of MMLV reverse transcriptase (Evrogen, Moscow, Russia). The reaction mixture was incubated at 37 °C for 40 min and then at 42 °C for 20 min. The resulting cDNA was then added to 475 μL of a mixture containing 20 μL of high-fidelity Tersus DNA polymerase (Evrogen Moscow, Russia), Tersus reaction buffer, dNTPs (0.2 mM of each), 12 pmol of the primer-adapter (R), and 12 pmol of B2-specific primer 2-adapter (F) (Table S7). The mixture was then distributed into 20 tubes and cDNA was amplified by PCR using the following program: 95 °C for 1 min, 25 cycles of 95 °C for 30 s, 65 °C for 30 s, 72 °C for 1 min, followed by a final incubation at 72 °C for 7 min. Subsequent to the PCR, the reaction mixtures from PCR tubes were consolidated into a single Eppendorf tube (total volume 500 μL) and an equal volume of magnetic beads suspension (Agencourt AMPure XP; Beckman Coulter, Brea, CA, USA) was added. Double-stranded cDNA molecules longer than 100 bp were bound to the beads. Subsequent to this step, the beads were washed with 70% ethanol. Thereafter, the cDNA was eluted with 80 μL of TE buffer (Tris-HCl, pH 8.0, 0.1 mM EDTA). This procedure yielded approximately 5 µg of the cDNA library. The libraries were sequenced as described in Method 1, except the read length was 300 bp.

### 4.4. Bioinformatics Analysis

The identification of genomic B2 copies in the mouse genome was conducted using the SSEARCH36 program from the FASTA package [50], with the B2a subfamily consensus sequence as the query and a similarity threshold of 65% over 90% of the sequence length. This search was executed in multiple iterative rounds, with identified copies being systematically removed from the original FASTA genome sequence following each round, until no further copies were detected. The genomic copies of B2 were then assigned to specific subfamilies based on the highest pairwise bitscore values, which were obtained by comparing the copies to a subfamily consensus, as calculated using the SSEARCH36 program.

Subsequently, raw Illumina reads were mapped to the GRCm38.p6 mouse genome assembly, excluding alternate scaffolds. This was done using BWA-MEM with default parameters [51]. Only reads that overlapped with the coordinates of identified B2 SINE copies were analyzed. The mapping results were then filtered to retain alignments with a minimum match length of 100 bp and a quality score of at least 25, which thereby removed partial and multi-mapping matches (Table S2). The number of reads per genomic B2 SINE copy was subsequently calculated using the bedtools merge tool with the parameter -d -20, which merges intervals that overlap by at least 20 bp [52].

Sequence alignments were performed using the MAFFT program [53] and subsequently visualized in GeneDoc (http://www.nrbsc.org/gfx/genedoc/index.html) (accessed on 1 December 2024). Venn diagrams were generated using the web application available at https://bioinformatics.psb.ugent.be/webtools/Venn/ (accessed on 1 February 2025).

## Figures and Tables

**Table 1:** B2 copies selected for analysis of 3′-ends of their transcripts in L929 cells.

Copy Number	Coordinates of B2 Copy in the Mouse Genome	Terminator (Uppercase) and Its Flanking Sequences (Lowercase)	Reads in the Library (Number of Reads Analyzed)
copy 1	chr1:33718543–33718720	taaaTCTTTTTTTTTTTTTTTTaagttctctgact	8733 (300)
copy 2	chr7:43662585–43662767	taaaTCTTTTTTTTTTTTTTaaatctgtgt	1880 (300)
copy 3	chr1:39288250–39288427	taaaTCTTTTTTTTTaagagtctggg	648 (300)
copy 4	chr7:79747308–79747485	taaaTGTTTTTTTaaaagtatgtatgtc	17,195 (300)
copy 5	chr13:24776694–24776875	taaaTATTTTTTaaaagaccatgccata	6303 (300)
copy 6	chr11:120514490–120514667	taaaTATTTTTTTaaaaaatgactgaag	7358 (300)
copy 7	chr11:87432668–87432843	caaaTTTTTTTcttttttttaaattaggtatttt	1996 (300)
copy 8 *	chr8:111186123–111186298	aaaaTTTTTTTTTgtacagtctataacaattt	791 (131)
copy 9	chr15:102348646–102348820	taaaTTTTTTTTTaaaaaagatatcagtaagg	4797 (300)
copy 10	chr6:73015125–73015298	taaaTCTTTaaaaaaatttcatagtctg	12,839 (284)
copy 11	chr5:121594718–121594889	taaaTCTTTagactgaagccagcgggg	254 (126)
copy 12	chr3:107861564–107861740	taaaTCTTTgggccggagccagcag	330 (45)
copy 13 *	chr8:111186123–111186298	taaaTCTTTaaaaaatttttttttgtac	791 (169)
copy 14	chr12:40255314–40255487	taaaTCTTTTTaaaaaatatgtgcacca	393 (336)
copy 15	chr2:25257465–25257635	taaaTTTTTaaaaattagggtgttatgca	351 (300)
copy 16	chr10:22009277–22009448	aaaaTTTTTagtgaatatcttgtttgtatt	35 (26)
copy 17	chr9:60734676–60734853	taaaTTTTTaaaaatcccctcccatgatg	1704 (300)
copy 18	chr9:49043798–49043971	ttaaTTTTTaaaaaagcctgtgagaaaa	232 (184)
copy 19	chr2:32853715–32853892	taaaTATTTTaaaaaaaccatccagag	4115 (300)

* The names “copy 13” and “copy 8” refer to the same B2 copy, but each refers to one of its two terminators (TCTTT and T_9_), which are separated by six A residues.

**Table 2:** B2 copies selected for analysis of 3′-ends of their transcripts in testis.

Copy Number	Coordinates of B2 Copy in the Mouse Genome	Terminator (Uppercase) and Its Flanking Sequences (Lowercase)	Reads in the Library (Number of Reads Analyzed)
copy 20	chr4:134947766–134947944	taaaTCTTTTTTTTTaagaaagaaaagg	7024 (1000)
copy 21	chr17:32112666–32112839	taaaTCTTTTTTTTTaaaagaaatgtttagc	4393 (188)
copy 22	chr11:87503376–87503546	taaaTCTTTTTTaaaaaggaggtaatgaactg	680 (420)
copy 23	chr1:58466632–58466809	taaaTATTTTTTTaagaaaaataagtgctag	923 (600)
copy 24	chr11:97993976–97994153	taaaTATTTTTTTaaaaatgtcttaccagatg	67,195 (190)
copy 25	chr9:65300382–65300568	aaataaTTTTTTTaaagtacaatgagtatat	183 (183)
copy 26	chr11:89376866–89377041	taaataaTTTTTTaaaaaaatagataatc	735 (200)
copy 27	chr13:99830672–99830886	taaaTCTTTTaaaaaaagtactaaa	730 (188)
copy 28	chr4:129762801–129762978	taaaTCTTTTaaaaaaaattttttaaaaagaaaga	268 (200)
copy 29	chr1:133036101–133036282	ataaTATTTTaaaaaaacaaacaaacaa	20,212 (193)
copy 30	chr15:36647144–36647318	taaaTGTTTTaaagactgaatagttagcaaatg	1579 (200)
copy 31	chr15:82218406–82218583	taaaTCTTTgtgtgccctctgtgga	739 (156)
copy 32	chr4:44554747–44554923	taaaTGTTTgcttcaagcagctgttggaatcac	2087 (147)

## Data Availability

The data generated in this study have been deposited in the NCBI BioProject database under ac-cession number PRJNA1241838.

## Supplementary Materials

The following supporting information can be downloaded at: https://www.mdpi.com/article/10.3390/ncrna11030039/s1, Figure S1: The alignment of consensus nucleotide sequences of five B2 SINE subfamilies from the mouse genome; Figure S2: The nucleotide sequences of 200 reads were obtained from the sequencing of the seminal cDNA library and mapped to the genomic B2 copy (Chr15:82218345–82218558) in the mouse genome; Figure S3: The nucleotide sequences of 172 reads were obtained from the sequencing of the seminal cDNA library and mapped to the genomic B2 copy (chr4:44554747–44554923 in the mouse genome; Figure S4: Comparison of transcriptional arrest at terminators of individual B2 copies in L929 cells and testis; Figure S5: Comparison of transcription arrest at terminators of individual B2 copies in L929, 4T1 cells, brain, and testis; Figure S6: B2 copies without their own functional terminators that are transcribed by pol III to downstream terminators and the resulting transcripts can be polyadenylated; Figure S7: Distribution of reads by B2 copy categories; Table S1: B2 copies identified as transcribed by Method 1 (worksheet 1) and Method 2 (worksheet 2); Table S2: Analysis of the number of reads obtained by NGS of B2 cDNA libraries; Table S3: A sample 1 of B2 copies identified by analysis of cDNA libraries obtained by method 2 for L929, 4T1, brain, and testes cells in mouse; Table S4: A sample 2 of B2 copies identified by analysis of cDNA libraries obtained by method 2 for L929, 4T1, brain, and testes cells in mouse; Table S5: A sample 3 of B2 copies identified by analysis of cDNA libraries obtained by method 2 for L929, 4T1, brain, and testes cells in mouse; Table S6: Oligonucleotides utilized in the preparation of B2 cDNA libraries by Method 1; Table S7: Oligonucleotides utilized in the preparation of B2 cDNA libraries by Method 2.

## Abbreviations

The following abbreviations are used in this manuscript:

NGS	Next-Generation Sequencing
SINE	Short Interspersed Element
LINE	Long Interspersed Element
pol III	RNA polymerase III
pol II	RNA polymerase II
PA	Polyadenylation
PAS	Polyadenylation signal
mPSF	mammalian Polyadenylation Specificity Factor
CFIm	Cleavage Factor I mammalian

## References

[1] Kramerov D.A., Vassetzky N.S. (2011). SINEs. Wiley Interdiscip. Rev. RNA.

[2] Deininger P. (2011). Alu elements: Know the SINEs. Genome Biol..

[3] Vassetzky N.S., Kramerov D.A. (2013). SINEBase: A database and tool for SINE analysis. Nucleic Acids Res..

[4] Chen J.M., Ferec C., Cooper D.N. (2006). LINE-1 endonuclease-dependent retrotranspositional events causing human genetic disease: Mutation detection bias and multiple mechanisms of target gene disruption. J. Biomed. Biotechnol..

[5] Ferrigno O., Virolle T., Djabari Z., Ortonne J.P., White R.J., Aberdam D. (2001). Transposable B2 SINE elements can provide mobile RNA polymerase II promoters. Nat. Genet..

[6] Su M., Han D., Boyd-Kirkup J., Yu X., Han J.J. (2014). Evolution of Alu elements toward enhancers. Cell Rep..

[7] Policarpi C., Crepaldi L., Brookes E., Nitarska J., French S.M., Coatti A., Riccio A. (2017). Enhancer SINEs Link Pol III to Pol II Transcription in Neurons. Cell Rep..

[8] Krull M., Brosius J., Schmitz J. (2005). Alu-SINE exonization: En route to protein-coding function. Mol. Biol. Evol..

[9] Wang W., Kirkness E.F. (2005). Short interspersed elements (SINEs) are a major source of canine genomic diversity. Genome Res..

[10] Chen C., Ara T., Gautheret D. (2009). Using Alu elements as polyadenylation sites: A case of retroposon exaptation. Mol. Biol. Evol..

[11] Choi J.D., Del Pinto L.A., Sutter N.B. (2025). SINE retrotransposons import polyadenylation signals to 3′UTRs in dog (*Canis familiaris*). Mob. DNA.

[12] Zovoilis A., Cifuentes-Rojas C., Chu H.P., Hernandez A.J., Lee J.T. (2016). Destabilization of B2 RNA by EZH2 Activates the Stress Response. Cell.

[13] Ponicsan S.L., Kugel J.F., Goodrich J.A. (2010). Genomic gems: SINE RNAs regulate mRNA production. Curr. Opin. Genet. Dev..

[14] Bartlett A.A., Guffanti G., Hunter R.G. (2023). B2 SINE RNA as a novel regulator of glucocorticoid receptor transcriptional activity. Neurobiol. Stress.

[15] Haynes S.R., Jelinek W.R. (1981). Low molecular weight RNAs transcribed in vitro by RNA polymerase III from Alu-type dispersed repeats in Chinese hamster DNA are also found in vivo. Proc. Natl. Acad. Sci. USA.

[16] Krayev A.S., Markusheva T.V., Kramerov D.A., Ryskov A.P., Skryabin K.G., Bayev A.A., Georgiev G.P. (1982). Ubiquitous transposon-like repeats B1 and B2 of the mouse genome: B2 sequencing. Nucleic Acids Res..

[17] Daniels G.R., Deininger P.L. (1985). Repeat sequence families derived from mammalian tRNA genes. Nature.

[18] Sakamoto K., Okada N. (1985). Rodent type 2 Alu family, rat identifier sequence, rabbit C family, and bovine or goat 73-bp repeat may have evolved from tRNA genes. J. Mol. Evol..

[19] Vassetzky N.S., Borodulina O.R., Ustyantsev I.G., Kosushkin S.A., Kramerov D.A. (2021). Analysis of SINE Families B2, Dip, and Ves with Special Reference to Polyadenylation Signals and Transcription Terminators. Int. J. Mol. Sci..

[20] Jurka J., Kapitonov V.V., Pavlicek A., Klonowski P., Kohany O., Walichiewicz J. (2005). Repbase Update, a database of eukaryotic repetitive elements. Cytogenet. Genome Res..

[21] Ichiyanagi T., Katoh H., Mori Y., Hirafuku K., Boyboy B.A., Kawase M., Ichiyanagi K. (2021). B2 SINE Copies Serve as a Transposable Boundary of DNA Methylation and Histone Modifications in the Mouse. Mol. Biol. Evol..

[22] Sakamoto K., Kominami R., Mishima Y., Okada N. (1984). The 6S RNA transcribed from rodent total DNA in vitro is the transcript of the type 2 Alu family. Mol. Gen. Genet..

[23] Kramerov D.A., Lekakh I.V., Samarina O.P., Ryskov A.P. (1982). The sequences homologous to major interspersed repeats B1 and B2 of mouse genome are present in mRNA and small cytoplasmic poly(A) + RNA. Nucleic Acids Res..

[24] Bladon T.S., Fregeau C.J., McBurney M.W. (1990). Synthesis and processing of small B2 transcripts in mouse embryonal carcinoma cells. Mol. Cell. Biol..

[25] Kramerov D.A., Tillib S.V., Shumyatsky G.P., Georgiev G.P. (1990). The most abundant nascent poly(A) + RNAs are transcribed by RNA polymerase III in murine tumor cells. Nucleic Acids Res..

[26] Borodulina O.R., Kramerov D.A. (2001). Short interspersed elements (SINEs) from insectivores. Two classes of mammalian SINEs distinguished by A-rich tail structure. Mamm. Genome.

[27] Borodulina O.R., Golubchikova J.S., Ustyantsev I.G., Kramerov D.A. (2016). Polyadenylation of RNA transcribed from mammalian SINEs by RNA polymerase III: Complex requirements for nucleotide sequences. Biochim. Biophys. Acta.

[28] Ustyantsev I.G., Kosushkin S.A., Borodulina O.R., Vassetzky N.S., Kramerov D.A. (2024). Ere, a Family of Short Interspersed Elements in the Genomes of Odd-Toed Ungulates (Perissodactyla). Animals.

[29] Ustyantsev I.G., Borodulina O.R., Kramerov D.A. (2021). Identification of nucleotide sequences and some proteins involved in polyadenylation of RNA transcribed by Pol III from SINEs. RNA Biol..

[30] Ustyantsev I.G., Borodulina O.R., Kramerov D.A. (2024). Participation of Proteins of the CPSF Complex in Polyadenylation of Transcripts Read by RNA Polymerase III from SINEs. Mol. Biol..

[31] Ustyantsev I.G., Tatosyan K.A., Stasenko D.V., Kochanova N.Y., Borodulina O.R., Kramerov D.A. (2020). [Polyadenylation of Sine Transcripts Generated by RNA Polymerase III Dramatically Prolongs Their Lifetime in Cells]. Mol. Biol..

[32] Dewannieux M., Heidmann T. (2005). Role of poly(A) tail length in Alu retrotransposition. Genomics.

[33] Roy-Engel A.M., Salem A.H., Oyeniran O.O., Deininger L., Hedges D.J., Kilroy G.E., Batzer M.A., Deininger P.L. (2002). Active Alu element “A-tails”: Size does matter. Genome Res..

[34] Roy-Engel A.M. (2012). LINEs, SINEs and other retroelements: Do birds of a feather flock together?. Front. Biosci. (Landmark Ed.).

[35] Wagstaff B.J., Hedges D.J., Derbes R.S., Campos Sanchez R., Chiaromonte F., Makova K.D., Roy-Engel A.M. (2012). Rescuing Alu: Recovery of new inserts shows LINE-1 preserves Alu activity through A-tail expansion. PLoS Genet..

[36] Gogolevskaya I.K., Kramerov D.A. (2002). Evolutionary history of 4.5SI RNA and indication that it is functional. J. Mol. Evol..

[37] Karijolich J., Zhao Y., Alla R., Glaunsinger B. (2017). Genome-wide mapping of infection-induced SINE RNAs reveals a role in selective mRNA export. Nucleic Acids Res..

[38] Orioli A., Pascali C., Quartararo J., Diebel K.W., Praz V., Romascano D., Percudani R., van Dyk L.F., Hernandez N., Teichmann M. (2011). Widespread occurrence of non-canonical transcription termination by human RNA polymerase III. Nucleic Acids Res..

[39] Linker S.B., Randolph-Moore L., Kottilil K., Qiu F., Jaeger B.N., Barron J., Gage F.H. (2020). Identification of bona fide B2 SINE retrotransposon transcription through single-nucleus RNA-seq of the mouse hippocampus. Genome Res..

[40] Oomen M.E., Rodriguez-Terrones D., Kurome M., Zakhartchenko V., Mottes L., Simmet K., Noll C., Nakatani T., Mourra-Diaz C.M., Aksoy I. (2025). An atlas of transcription initiation reveals regulatory principles of gene and transposable element expression in early mammalian development. Cell.

[41] Proudfoot N.J. (2011). Ending the message: Poly(A) signals then and now. Genes Dev..

[42] Sun Y., Hamilton K., Tong L. (2020). Recent molecular insights into canonical pre-mRNA 3′-end processing. Transcription.

[43] Zhu Y., Wang X., Forouzmand E., Jeong J., Qiao F., Sowd G.A., Engelman A.N., Xie X., Hertel K.J., Shi Y. (2018). Molecular Mechanisms for CFIm-Mediated Regulation of mRNA Alternative Polyadenylation. Mol. Cell.

[44] Tian B., Hu J., Zhang H., Lutz C.S. (2005). A large-scale analysis of mRNA polyadenylation of human and mouse genes. Nucleic Acids Res..

[45] Borodulina O.R., Ustyantsev I.G., Kramerov D.A. (2023). SINEs as Potential Expression Cassettes: Impact of Deletions and Insertions on Polyadenylation and Lifetime of B2 and Ves SINE Transcripts Generated by RNA Polymerase III. Int. J. Mol. Sci..

[46] Pulaski B.A., Ostrand-Rosenberg S. (2001). Mouse 4T1 breast tumor model. Curr. Protoc. Immunol..

[47] Senin V.M., Buntsevich A.M., Afanasyeva A.V., Kiseleva N.S. (1983). A new line of murine carcinosarcoma. Exp. Oncol. USSR.

[48] Ebralidze A., Tulchinsky E., Grigorian M., Afanasyeva A., Senin V., Revazova E., Lukanidin E. (1989). Isolation and characterization of a gene specifically expressed in different metastatic cells and whose deduced gene product has a high degree of homology to a Ca2+-binding protein family. Genes Dev..

[49] Chomczynski P., Sacchi N. (2006). The single-step method of RNA isolation by acid guanidinium thiocyanate-phenol-chloroform extraction: Twenty-something years on. Nat. Protoc..

[50] Pearson W.R., Lipman D.J. (1988). Improved tools for biological sequence comparison. Proc. Natl. Acad. Sci. USA.

[51] Li H. (2013). Aligning sequence reads, clone sequences and assembly contigs with BWA-MEM. arXiv.

[52] Quinlan A.R., Hall I.M. (2010). BEDTools: A flexible suite of utilities for comparing genomic features. Bioinformatics.

[53] Yamada K.D., Tomii K., Katoh K. (2016). Application of the MAFFT sequence alignment program to large data-reexamination of the usefulness of chained guide trees. Bioinformatics.

