# Supplementary material for: Analysis of RNA Transcribed by RNA Polymerase III from B2 SINEs in Mouse Cells
Source: Noncoding RNA. 2025 May 14;11(3):39. doi: 10.3390/ncrna11030039 (PMC12101331; doi:10.3390/ncrna11030039)
Supplement: Supplementary file 1 [file ncrna-11-00039-s001.zip › ncrna-3586305-supplementary/Figure S1.pdf]

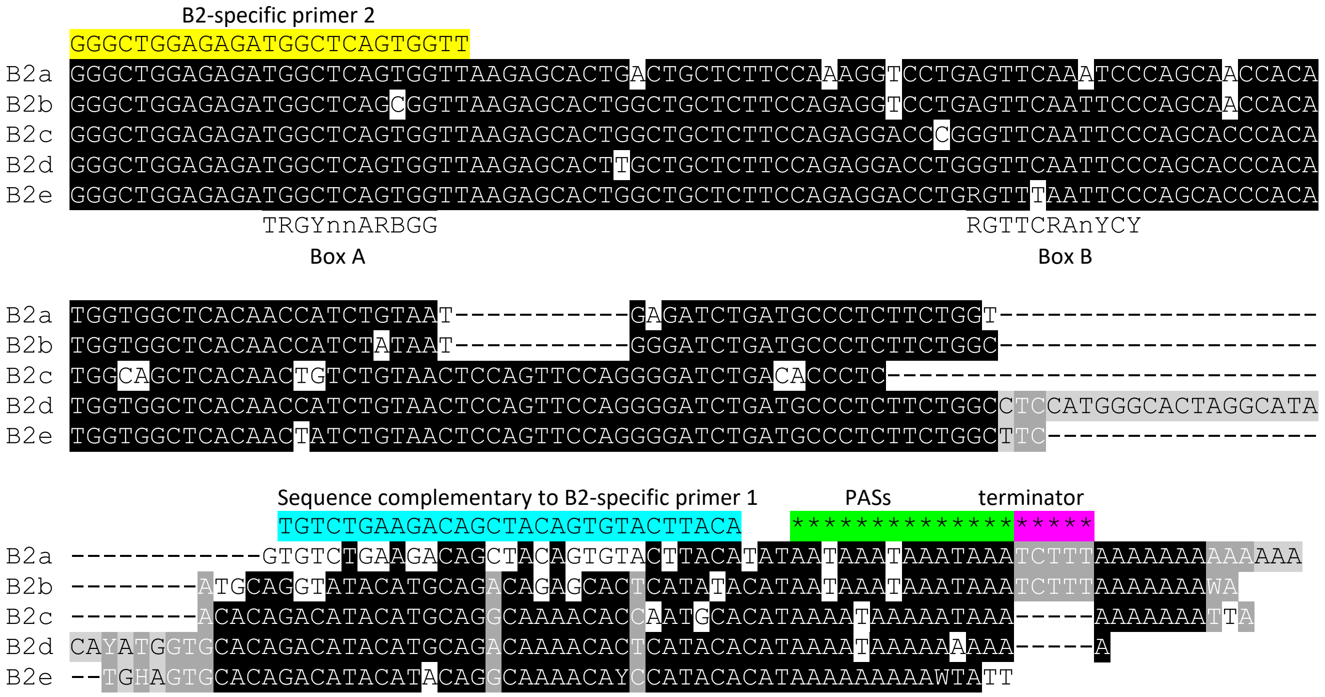

Figure S1. The alignment of consensus nucleotide sequences of five B2 SINE subfamilies from the mouse genome [19]. Consensus sequences of boxes A and B of pol III promoter are shown below the alignment. Positions of polyadenylation signals (PASs) and the pol III transcription terminator are indicated by asterisks. B2-specific primer 2 (yellow) and the sequence complementary to B2-specific primer 1 (blue) are shown above the alignment. B2-specific primer 1 and B2-specific primer 2 were used in methods 1 and 2, respectively (see Figures 2 and 4).
