# Supplementary material for: Analysis of RNA Transcribed by RNA Polymerase III from B2 SINEs in Mouse Cells
Source: Noncoding RNA. 2025 May 14;11(3):39. doi: 10.3390/ncrna11030039 (PMC12101331; doi:10.3390/ncrna11030039)
Supplement: Supplementary file 1 [file ncrna-11-00039-s001.zip › ncrna-3586305-supplementary/Figure S2.pdf]

```
 1: AGTGTTCTCATATAAATAAAATAAATAAATCTT---------------------------------------------------------------------AAAAAAAAAAAAAAAAAAA----AATCGGAAGAGCACACG
 2: AGTGTTCTCATATAAATAAAATAAATAAATCTTT------------------------------------------------------------------------AAAAAAAAAAAAAAA----GATCGGAAGAGCACACG
 3: AGTGTTCTCATATAAATAAAATAAATAAATCTTT----------------------------------------------------------------------AAAAAAAAAAAAAAAAA----GATCGGAAGAGCACACG
 4: AGTGTTCTCATATAAATAAAATAAATAAATCTTT----------------------------------------------------------------------AAAAAAAAAAAAAAAAA----GATCGGAAGAGCACACG
 5: AGTGTTCTCATATAAATAAAATAAATAAATCTTT----------------------------------------------------------------------AAAAAAAAAAAAAAAAA----GATCGGAAGAGCACACG
 6: AGTGTTCTCATATAAATAAAATAAATAAATCTTT------------------------------------------------------------------------AAAAAAAAAAAAAAA----GATCGGAAGAGCACACG
 7: AGTGTTCTCATATAAATAAAATAAATAAATCTTT------------------------------------------------------------------------AAAAAAAAAAAAAAA----GATCGGAAGAGCACACG
 8: AGTGTTCTCATATAAATAAAATAAATAAATCTTT------------------------------------------------------------------------AAAAAAAAAAAAAAA----GATCGGAAGAGCACACG
 9: AGTGTTCTCATATAAATAAAATAAATAAATCTTT------------------------------------------------------------------------AAAAAAAAAAAAAAA----GATCGGAAGAGCACACG
10: AGTGTTCTCATATAAATAAAATAAATAAATCTTT------------------------------------------------------------------------AAAAAAAAAAAAAAA----GATCGGAAGAGCACACG
11: AGTGTTCTCATATAAATAAAATAAATAAATCTTT------------------------------------------------------------------------AAAAAAAAAAAAAAA----GATCGGAAGAGCACACG
12: AGTGTTCTCATATAAATAAAATAAATAAATCTTT------------------------------------------------------------------------AAAAAAAAAAAAAAA----GATCGGAAGAGCACACG
13: AGTGTTCTCATATAAATAAAATAAATAAATCTTT------------------------------------------------------------------------AAAAAAAAAAAAAAA----GATCGGAAGAGCACACG
14: AGTGTTCTCATATAAATAAAATAAATAAATCTTT------------------------------------------------------------------------AAAAAAAAAAAAAAA----GATCGGAAGAGCACACG
15: AGTGTTCTCATATAAATAAAATAAATAAATCTTT------------------------------------------------------------------------AAAAAAAAAAAAAAA----GATCGGAAGAGCACACG
16: AGTGTTCTCATATAAATAAAATAAATAAATCTTT-------------------------------------------------------------------------AAAAAAAAAAAAAA----GATCGGAAGAGCACACG
17: AGTGTTCTCATATAAATAAAATAAATAAATCTTT----------------------------------------------------------------------AAAAAAAAAAAAAAAAA----GATCGGAAGAGCACACG
18: AGTGTTCTCATATAAATAAAATAAATAAATCTTT-------------------------------------------------------------------------AAAAAAAAAAAAAA----GATCGGAAGAGCACACG
19: AGTGTTCTCATATAAATAAAATAAATAAATCTTT-----------------------------------------------------------------------AAAAAAAAAAAAAAAA----GATCGGAAGAGCACACG
20: AGTGTTCTCATATAAATAAAATAAATAAATCTTT-----------------------------------------------------------------------AAAAAAAAAAAAAAAA----GATCGGAAGAGCACACG
21: AGTGTTCTCATATAAATAAAATAAATAAATCTTT-----------------------------------------------------------------------AAAAAAAAAAAAAAAA----GATCGGAAGAGCACACG
22: AGTGTTCTCATATAAATAAAATAAATAAATCTTT---------------------------------------------------------------------AAAAAAAAAAAAAAAAAA----GATCGGAAGAGCACACG
23: AGTGTTCTCATATAAATAAAATAAATAAATCTTT------------------------------------------------------------------------AAAAAAAAAAAAAAA----GATCGGAAGAGCACACG
24: AGTGTTCTCATATAAATAAAATAAATAAATCTTT-----------------------------------------------------------------------AAAAAAAAAAAAAAAA----GATCGGAAGAGCACACG
25: AGTGTTCTCATATAAATAAAATAAATAAATCTT------------------------------------------------------------------------AAAAAAAAAAAAAAAA----GATCGGAAGAGCACACG
26: AGTGTTCTCATATAAATAAAATAAATAAATCTT-------------------------------------------------------------------------AAAAAAAAAAAAAAA----GATCGGAAGAGCACACG
27: AGTGTTCTCATATAAATAAAATAAATAAATCTTT-------------------------------------------------------------------------AAAAAAAAAAAAAA----GATCGGAAGAGCACACG
28: AGTGTTCTCATATAAATAAAATAAATAAATCTTT---------------------------------------------------------------------AAAAAAAAAAAAAAAAAA----GATCGGAAGAGCACACG
29: AGTGTTCTCATATAAATAAAATAAATAAATCTTT---------------------------------------------------------------------AAAAAAAAAAAAAAAAAA----GATCGGAAGAGCACACG
30: AGTGTTCTCATATAAATAAAATAAATAAATCTTT---------------------------------------------------------------------AAAAAAAAAAAAAAAAAA----GATCGGAAGAGCACACG
31: AGTGTTCTCATATAAATAAAATAAATAAATCTTT-------------------------------------------------------------------AAAAAAAAAAAAAAAAAAAA----GATCGGAAGCGCACACG
32: AGTGTTCTCATATAAATAAAATAAATAAATCTTT-------------------------------------------------------------------AAAAAAAAAAAAAAAAAAAA----GATCGGAAGAGCACACG
33: AGTGTTCTCATATAAATAAAATAAATAAATCTTT-----------------------------------------------------------------AAAAAAAAAAAAAAAAAAAAAA----GATCGGAAGAGCACACG
34: AGTGTTCTCATATAAATAAAATAAATAAATCTTT--------------------------------------------------------------------------AAAAAAAAAAAAA----GATCGGAAGAGCACACG
35: AGTGTTCTCATATAAATAAAATAAATAAATCTTT--------------------------------------------------------------------AAAAAAAAAAAAAAAAAAA----GATCGGAAGAGCACACG
36: AGTGTTCTCATATAAATAAAATAAATAAATCTTT---------------------------------------------------------------------------AAAAAAAAAAAA----GATCGGAAGAGCACACG
37: AGTGTTCTCATATAAATAAAATAAATAAATCTT----------------------------------------------------------------------------AAAAAAAAAAAA----GATCGGAAGAGCACACG
38: AGTGTTCTCATATAAATAAAATAAATAAATCTTT-------------------------------------------------------------------------AAAAAAAAAAAAAA----GATCGGAAGAGCACACG
39: AGTGTTCTCATATAAATAAAATAAATAAATCTTT--------------------------------------------------------------------AAAAAAAAAAAAAAAAAAA----GATCGGAAGAGCACACG
40: AGTGTTCTCATATAAATAAAATAAATAAATCTTT----------------------------------------------------------------------AAAAAAAAAAAAAAAAA----GATCGGAAGAGCACACG
41: AGTGTTCTCATATAAATAAAATAAATAAATCTTT-----------------------------------------------------------------------AAAAAAAAAAAAAAAA----GATCGGAAGAGGACACG
42: AGTGTTCTCATATAAATAAAATAAATAAATCTTT-----------------------------------------------------------------------AAAAAAAAAAAAAAAA----GATCGGAGGAGCACACG
43: AGTGTTCTCATATAAATAAAATAAATAAATCTT-----------------------------------------------------------------------AAAAAAAAAAAAAAAAA----GATCGAAAGAGCACACG
44: AGTGTTCTCATATAAATAAAATAAATAAATCTTT-----------------------------------------------------------------------AAAAAAAAAAAAAAAA----GATCGGAAGAGCACACG
45: AGTGTTCTCATATAAATAAAATAAATAAATCTTT-------------------------------------------------------------------AAAAAAAAAAAAAAAAAAAA----GATCGGAAGAGCACACC
46: AGTGTTCTCATATAAATAAAATAAATAAATCTTT-------------------------------------------------------------------AAAAAAAAAAAAAAAAAAAA----GATCGGAAGAGCACACG
47: AGTGTTCTCATATAAATAAAATAAATAAATCTTT-------------------------------------------------------------------AAAAAAAAAAAAAAAAAAAA----GATCGGAAGAGCACACG
48: AGTGTTCTCATATAAATAAAATAAATAAATCTT-------------------------------------------------------------------------AAAAAAAAAAAAAAA----GATCGGAAGAGCACACG
49: AGTGTTCTCATATAAATAAAATAAATAAATCTTT----------------------------------------------------------------------AAAAAAAAAAAAAAAAA----GATCGGAAGAGCACACG
50: AGTGTTCTCATATAAATAAAATAAATAAATCTTT-----------------------------------------------------------------------AAAAAAAAAAAAAAAA----GATCGGAAGAGCCCACG
51: AGTGTTCTCATATAAATAAAATAAATAAATCTTT----------------------------------------------------------------------AAAAAAAAAAAAAAAAA----GATCGGAAGAGCACACG
52: AGTGTTCTCATATAAATAAAATAAATAAATCTTT----------------------------------------------------------------------AAAAAAAAAAAAAAAAA----GATCGGAAGAGCACACG
53: AGTGTTCTCATATAAATAAAATAAATAAATCTTT---------------------------------------------------------------------AAAAAAAAAAAAAAAAAA----GACCGGA-GAGCACACG
54: AGTGTTCTCATATAAATAAAATAAATAAATCTTT--------------------------------------------------------------------AAAAAAAAAAAAAAAAAAA----GATCGGAAA-GCACACG
55: AGTGTTCTCATATAAATAAAATAAATAAATCTTT--------------------------------------------------------------------AAAAAAAAAAAAAAAAAAA----GATCGGAAGAGCACACG
56: AGTGTTCTCATATAAATAAAATAAATAAATCTTT---------------------------------------------------------------------AAAAAAAAAAAAAAAAAA----GATC-GGAAGGCACACG
57: AGTGTTCTCATATAAATAAAATAAATAAATCTTT----------------------------------------------------------------------AAAAAAAAAAAAAAAAA----AGATCGGAAAGCACACG
```

```
 58: AGTGTTCTCATATAAATAAAATAAATAAATCTTT----------------------------------------------------------------------AAAAAAAAAAAAAAAAA----GATCGGAAGAGCACACT
 59: AGTGTTCTCATATAAATAAAATAAATAAATCTTT----------------------------------------------------------------------AAAAAAAAAAAAAAAAA----GATCGGGAGGGCACACC
 60: AGTGTTCTCATATAAATAAAATAAATAAATCTTT--------------------------------------------------------------------AAAAAAAAAAAAAAAAAAA----GATCGGAAGAGCACACG
 61: AGTGTTCTCATATAAATAAAATAAATAAATCTTT--------------------------------------------------------------------AAAAAAAAAAAAAAAAAAA----GATCGGAAGAGCACACG
 62: AGTGTTCTCATATAAATAAAATAAATAAATCTTT--------------------------------------------------------------------AAAAAAAAAAAAAAAAAAA----GATCGGAAGAGCACACG
 63: AGTGTTCTCATATAAATAAAATAAATAAATCTTT------------------------------------------------------------------AAAAAAAAAAAAAAAAAAAAA----GATCGGAAGAGCACACG
 64: AGTGTTCTCATATAAATAAAATAAATAAATCTTT------------------------------------------------------------------AAAAAAAAAAAAAAAAAAAAA----GACCGGAAGAGCACACG
 65: AGTGTTCTCATATAAATAAAATAAATAAATCTT------------------------------------------------------------------AAAAAAAAAAAAAAAAAAAAAA----GACCGAAAGACCACACG
 66: AGTGTTCTCATATAAATAAAATAAATAAATCTTT-------------------------------------------------------------------------AAAAAAAAAAAAAA----GATCGGAAGAGCACACG
 67: AGTGTTCTCATATAAATAAAATAAATAAATCTTT--------------------------------------------------------------------AAAAAAAAAAAAAAAAAAA----GATCGGAAGAGCACACT
 68: AGTGTTCTCATATAAATAAAATAAATAAATCTTT--------------------------------------------------------------------AAAAAAAAAAAAAAAAAAA----GATCGGAAGAGCACACG
 69: AGTGTTCTCATATAAATAAAATAAATAAATCTTT---------------------------------------------------------------------AAAAAAAAAAAAAAAAAA----GATCGGA--AGCACACG
 70: AGTGTTCTCATATAAATAAAATAAATAAATCTTT---------------------------------------------------------------------AAAAAAAAAAAAAAAAAA----GATCGGAAGCGCACACG
 71: AGTGTTCTCATATAAATAAAATAAATAAATCTTT---------------------------------------------------------------------AAAAAAAAAAAAAAAAAA----GATCGGAAGAGCCCCCG
 72: AGTGTTCTCATATAAATAAAATAAATAAATCTTT-----------------------------------------------------------------------AAAAAAAAAAAAAAAA----GATCGGAAGAGCACACG
 73: AGTGTTCTCATATAAATAAAATAAATAAATCTTT---------------------------------------------------------------------AAAAAAAAAAAAAAAAAA----GATCGGAAGAGCACACG
 74: AGTGTTCTCATATAAATAAAATAAATAAATCTTT---------------------------------------------------------------------AAAAAAAAAAAAAAAAAA----GATCGGAAGAGCACACG
 75: AGTGTTCTCATATAAATAAAATAAATAAATCTTT---------------------------------------------------------------------AAAAAAAAAAAAAAAAAA----GATCGGAAGAGCACACT
 76: AGTGTTCTCATATAAATAAAATAAATAAATCTTT------------------------------------------------------------------AAAAAAAAAAAAAAAAAAAAA----GATCGGAAGAGCACACG
 77: AGTGTTCTCATATAAATAAAATAAATAAATCTTT-------------------------------------------------------------------AAAAAAAAAAAAAAAAAAAA----GACCGGAAGAGCCCCCT
 78: AGTGTTCTCATATAAATAAAATAAATAAATCTTT-------------------------------------------------------------------AAAAAAAAAAAAAAAAAAAA----GACCGGAAGAAACCACG
 79: AGTGTTCTCATATAAATAAAATAAATAAATCTTT----------------------------------------------------------------------AAAAAAAAAAAAAAAAA----GACCGGAAGAGCACACC
 80: AGTGTTCTCATATAAATAAAATAAATAAATCTTT----------------------------------------------------------------------AAAAAAAAAAAAAAAAA----AATCGGAAGAGCACACC
 81: AGTGTTCTCATATAAATAAAATAAATAAATCTT-----------------------------------------------------------------------AAAAAAAAAAAAAAAAA----GATCGGAAGAGCACACG
 82: AGTGTTCTCATATAAATAAAATAAATAAATCTTT-----------------------------------------------------------------------AAAAAAAAAAAAAAAA----GAACGGAAGAGCCAACG
 83: AGTGTTCTCATATAAATAAAATAAATAAATCTTT------------------------------------------------------------------------AAAAAAAAAAAAAAA----GATCGGAAGAGCACACG
 84: AGTGTTCTCATATAAATAAAATAAATAAATCTTT------------------------------------------------------------------------AAAAAAAAAAAAAAA----GACCGGATGACCACACG
 85: AGTGTTCTCATATAAATAAAATAAATAAATCTT-----------------------------------------------------------------------AAAAAAAAAAAAAAAAA----GATCGGAAGCGCACACG
 86: AGTGTTCTCATATAAATAAAATAAATAAATCTTT-----------------------------------------------------------------------AAAAAAAAAAAAAAAA----GATCGGAAGAGCACACG
 87: AGTGTTCTCATATAAATAAAATAAATAAATCTTT-----------------------------------------------------------------------AAAAAAAAAAAAAAAA----GAAC-GGAAAACAAACG
 88: AGTGTTCTCATATAAATAAAATAAATAAATCTTT------------------------------------------------------------------------AAAAAAAAAAAAAAA----GACCGGAAGAGCACACC
 89: AGTGTTCTCATATAAATAAAATAAATAAATCTTT------------------------------------------------------------------------AAAAAAAAAAAAAAA----GAACGGATGAGAACACG
 90: AGTGTTCTCATATAAATAAAATAAATAAATCTTT-----------------------------------------------------------------------AAAAAAAAAAAAAAAA----GATCGGAAGA-CACACG
 91: AGTGTTCTCATATAAATAAAATAAATAAATCTTT--------------------------------------------------------------------AAAAAAAAAAAAAAAAAAA----AATCGGAAGAGACCACG
 92: AGTGTTCTCATATAAATAAAATAAATAAATCTTT----------------------------------------------------------------------AAAAAAAAAAAAAAAAA----GACCGGAGGGCCACACG
 93: AGTGTTCTCATATAAATAAAATAAATAAATCTTT---------------------------------------------------------------AAAAAAAAAAAAAAAAAAAAAAAA----AACCGGAAAACACCCCT
 94: AGTGTTCTCATATAAATAAAATAAATAAATCTTT------------------------------------------------------------------AAAAAAAAAAAAAAAAAAAAA----AACCGGAAGAGCCACCC
 95: AGTGTTCTCATATAAATAAAATAAATAAATCTTT------------------------------------------------------------------AAAAAAAAAAAAAAAAAAAAG----GACGGGAGAGCCCCCCT
 96: AGTGTTCTCATATAAATAAAATAAATAAATCTTT-----------------------------------------------------------------------AAAAAAAAAAAAAAAA----GATTGGGAGGACCCCCC
 97: AGTGTTCTCATATAAATAAAATAAATAAATCTTT-----------------------------------------------------------------------AAAAAAAAAAAAAAAA----GATCGGAAGAGCACACG
 98: AGTGTTCTCATATAAATAAAATAAATAAATCTTT----------------------------------------------------------------------AAAAAAAAAAAAAAAAA----AGATGGAAGAGCACCCC
 99: AGTGTTCTCATATAAATAAAATAAATAAATCTTT----------------------------------------------------------------------AAAAAAAAAAAAAAAAA----GATCGGAAGAGCACA-G
100: AGTGTTCTCATATAAATAAAATAAATAAATCTTT---------------------------------------------------------------------TAAAAAAAAAAAAAAAAA----AAACGGAAGAGCACACG
101: AGTGTTCTCATATAAATAAAATAAATAAATCTT-------------------------------------------------------------------AAAAAAAAAAAAAAAAAAAAA----GACCGGAAGACCACACG
102: AGTGTTCTCATATAAATAAAATAAATAAATCTTT-------------------------------------------------------------------AAAAAAAAAAAAAAAAAAAA----AACCGAAAAACCCCACA
103: AGTGTTCTCATATAAATAAAATAAATAAATCTTT-------------------------------------------------------------------AAAAAAAAAAAAAAAAAAAA----GATCGGAAACCCACACA
104: AGTGTTCTCATATAAATAAAATAAATAAATCTTT-------------------------------------------------------------------AAAAAAAAAAAAAAAAAAAA----GATCGGACAAGCACACG
105: AGTGTTCTCATATAAATAAAATAAATAAATCTTT--------------------------------------------------------------------AAAAAAAAAAAAAAAAAAA----GAACGGAAGAGCACACC
106: AGTGTTCTCATATAAATAAAATAAATAAATCTTT---------------------------------------------------------------------AAAAAAAAAAAAAAAAAA----GATCGGAAGCGCACACG
107: AGTGTTCTCATATAAATAAAATAAATAAATCTT-----------------------------------------------------------------------AAAAAAAAAAAAAAAAA----GATCGGAAGAGCACCCC
108: AGTGTTCTCATATAAATAAAATAAATAAATCTTT--------------------------------------------------------------------AAAAAAAAAAAAAAAAAAA----CATCGGAAGA-CACCCG
109: AGTGTTCTCATATAAATAAAATAAATAAATCTT----------------------------------------------------------------------AAAAAAAAAAAAAAAAAA----AACGGGAAGACCAAACG
110: AGTGTTCTCATATAAATAAAATAAATAAATCTTT------------------------------------------------------------------------AAAAAAAAAAAAAAA----AATCGGAAGAGAAC-CG
111: AGTGTTCTCATATAAATAAAATAAATAAATCTTT-----------------------------------------------------------------------AAAAAAAAAAAAAAAA----GAACGGAAGAGCACACG
112: AGTGTTCTCATATAAATAAAATAAATAAATCTTT-----------------------------------------------------------------------AAAAAAAAAAAAAAAG----ATTGGGATCAAGAACCG
113: AGTGTTCTCATATAAATAAAATAAATAAATCTTT-----------------------------------------------------------------------AAAAAAAAAAAAAAAA----GATCGGAAGAGAACACG
114: AGTGTTCTCATATAAATAAAATAAATAAATCTT--------------------------------------------------AAAAAAAAAAAAAAAAAAAAAAAAAAAAAAAAAAAAAA----AACCGCAAAAAACCAAC
```

```
115: AGTGTTCTCATATAAATAAAATAAATAAATCTTT---------------------------------------------------AAAAAAAAAAAAAAAAAAAAAAAAAAAAAAAAAAAA----AAACCGAAAAAAACACA
116: AGTGTTCTCATATAAATAAAATAAATAAATCTTT--------------------------------------------------AAAAAAAAAAAAAAAAAAAAAAAAAAAAAAAAAAAAA----AACGGAAAAAAAACAAC
117: AGTGTTCTCATATAAATAAAATAAATAAATCTTT-----------------------------------------AAAAAAAAAAAAAAAAAAAAAAAAAAAAAAAAAAAAAAAAAAAAAA------CAAAAAAAAACAAA-
118: AGTGTTCTCATATAAATAAAATAAATAAATCTTT-----------------------------------------AAAAAAAAAAAAAAAAAAAAAAAAAAAAAAAAAAAAAAAAAAAAAA------CAAAAAAAAAAA---
119: AGTGTTCTCATATAAATAAAATAAATAAATCTTT-------------------------AAAAAAAAAAAAAAAAAAAAAAAAAAAAAAAAAAAAAAAAAAAAAAAAAAAAAAAAAAAAAA---------------------
120: AGTGTTCTCATATAAATAAAATAAATAAATCTT-------------------------------------------------AAAAAAAAAAAAAAAAAAAAAAAAAAAAAAAAAAAAAAA----AAACGAAAAAACCCAAC
121: AGTGTTCTCATATAAATAAAATAAATAAATCTTT----------------------------------------------AAAAAAAAAAAAAAAAAAAAAAAAAAAAAAAAAAAAAAAAA-------CGAAAAACCCCCAC
122: AGTGTTCTCATATAAATAAAATAAATAAATCTTT--------------------------------------------------------AAAAAAAAAAAAAAAAAAAAAAAAAAAAAAA----AACCGAAAAACCAAACA
123: AGTGTTCTCATATAAATAAAATAAATAAATCTTT---------------------------------------------------------AAAAAAAAAAAAAAAAAAAAAAAAAAAAAA----AAACGGAAGAGCACACG
124: AGTGTTCTCATATAAATAAAATAAATAAATCTTT------------------------------------------------------AAAAAAAAAAAAAAAAAAAAAAAAAAAAAAAAA----AACCGGAAAAACACACC
125: AGTGTTCTCATATAAATAAAATAAATAAATCTTT------------------------------------------------------AAAAAAAAAAAAAAAAAAAAAAAAAAAAAAAAA----AATCGGAAGACCACACC
126: AGTGTTCTCATATAAATAAAATAAATAAATCTTT---------------------------------------------------AAAAAAAAAAAAAAAAAAAAAAAAAAAAAAAAAAAA----AAACGGAAAAACACAC-
127: AGTGTTCTCATATAAATAAAATAAATAAATCTTT-----------------------------------------------------------AAAAAAAAAAAAAAAAAAAAAAAAAAAA----AATCGGAAGAGCACCCC
128: AGTGTTCTCATATAAATAAAATAAATAAATCTTT---------------------------------------------------------------AAAAAAAAAAAAAAAAAAAAAAAA----GAACGGAAGAGCACACC
129: AGTGTTCTCATATAAATAAAATAAATAAATCTTT----------------------------------------------------------AAAAAAAAAAAAAAAAAAAAAAAAAAAAA----GATCGGAAGAGCACACG
130: AGTGTTCTCATATAAATAAAATAAATAAATCTTT------------------------------------------------------------AAAAAAAAAAAAAAAAAAAAAAAAAAA----AACAGGAGGGGAACCAA
131: AGTGTTCTCATATAAATAAAATAAATAAATCTTT----------------------------------------------------------AAAAAAAAAAAAAAAAAAAAAAAAAAAAA----ACGCGGAGACACCCACC
132: AGTGTTCTCATATAAATAAAATAAATAAATCTTT----------------------------------------------------------AAAAAAAAAAAAAAAAAAAAAAAAAAAAA----AATCGGAAACCCACACA
133: AGTGTTCTCATATAAATAAAATAAATAAATCT-------------------------------------------------------------AAAAAAAAAAAAAAAAAAAAAAAAAAAA----AAACGGAAGGGCCCCCC
134: AGTGTTCTCATATAAATAAAATAAATAAATCTTT---------------------------------------------------------------------AAAAAAAAAAAAAAAAAA----GATCGGAAGAGC-CCCT
135: AGTGTTCTCATATAAATAAAATAAATAAATCTTT----------------------------------------------------------------------AAAAAAAAAAAAAAAAA----GATCGGAAGAGCCCACG
136: AGTGTTCTCATATAAATAAAATAAATAAATCTT---------------------------------------------------------------AAAAAAAAAAAAAAAAAAAAAAAAT----GACGGAAGAAGAACTCC
137: AGTGTTCTCATATAAATAAAATAAATAAATCTTT----------------------------------------------------------------------AAAAAAAAAAAAAAAAA----GATCGGCAGAGCACACG
138: AGTGTTCTCATATAAATAAAATAAATAAATCTTT----------------------------------------------------------------------AAAAAAAAAAAAAAAAA----AATCGAAAACGACCACG
139: AGTGTTCTCATATAAATAAAATAAATAAATCTTT------------------------------------------------------------AAAAAAAAAAAAAAAAAAAAAAAAAAA----ACCCGGAATTGCCCCCC
140: AGTGTTCTCATATAAATAAAATAAATAAATCTTT-----------------------------------------------------------------AAAAAAAAAAAAAAAAAAAAAA----GATCGGACACGCCCCCG
141: AGTGTTCTCATATAAATAAAATAAATAAATCTT-------------------------------------------------------------------------AAAAAAAAAAAAAAA----AGATCGGGGAGCACACG
142: AGTGTTCTCATATAAATAAAATAAATAAATCTT------------------------------------------------------------------------AAAAAAAAAAAAAAAA----GATCGGAAGCGCACACG
143: AGTGTTCTCATATAAATAAAATAAATAAATCTTT-------------------------------------------------------------------AAAAAAAAAAAAAAAAAAAA----AGAGGGGAGAGTCCACC
144: AGTGTTCTCATATAAATAAAATAAATAAATCTTTG---------------------------------------------------------------AAAAAAAAAAAAAAAAAAAAAAA----AAACGGGAAAGGCCACA
145: AGTGTTCTCATATAAATAAAATAAATAAATCTTTG-----------------------------------------------------------------------AAAAAAAAAAAAAAA----GATCGGAAGAGCACACG
146: AGTGTTCTCATATAAATAAAATAAATAAATCTTTG----------------------------------------------------------------------AAAAAAAAAAAAAAAA----GATCGAAAGAGCACCCC
147: AGTGTTCTCATATAAATAAAATAAATAAATCTTTG----------------------------------------------------------------------AAAAAAAAAAAAAAAA----AAAAGGAAGGGAAAACG
148: AGTGTTCTCATATAAATAAAATAAATAAATCTTTG----------------------------------------------------------------------AAAAAAAAAAAAAAAA----GATCGGAAGAGCACACG
149: AGTGTTCTCATATAAATAAAATAAATAAATCTTTG----------------------------------------------------------------------AAAAAAAAAAAAAAAA----GATCGGAAGAGCACACG
150: AGTGTTCTCATATAAATAAAATAAATAAATCTTTG---------------------------------------------------------------------AAAAAAAAAAAAAAAAA----GATCGGAAGAGCAAACG
151: AGTGTTCTCATATAAATAAAATAAATAAATCTTTG------------------------------------------------------------------------AAAAAAAAAAAAAA----GATCGGAAGAGCACACG
152: AGTGTTCTCATATAAATAAAATAAATAAATCTTTG-----------------------------------------------------------------------AAAAAAAAAAAAAAA----GATCGGAAGAGCACACG
153: AGTGTTCTCATATAAATAAAATAAATAAATCTTTG----------------------------------------------------------------------AAAAAAAAAAAAAAAA----GATCGGAAGAGCACACG
154: AGTGTTCTCATATAAATAAAATAAATAAATCTTTG--------------------------------------------------------------------AAAAAAAAAAAAAAAAAA----GATCGGAAGCGCACACG
155: AGTGTTCTCATATAAATAAAATAAATAAATCTTTG-----------------------------------------------------------------------AAAAAAAAAAAAAAA----AAACGGAAGAGCAGACG
156: AGTGTTCTCATATAAATAAAATAAATAAATCTTTGG-----------------------------------------------------------------------AAAAAAAAAAAAAA----GTTCGTCCTGCCTCTCG
157: AGTGTTCTCATATAAATAAAATAAATAAATCTTTGT------------------------------------------------------------------AAAAAAAAAAAAAAAAAAA----GATCGGAGGAGCACACG
158: AGTGTTCTCATATAAATAAAATAAATAAATCTTTGT--------------------------------------------------------------------AAAAAAAAAAAAAAAAA----GATCGGAAGAGCACACG
159: AGTGTTCTCATATAAATAAAATAAATAAATCTTTGT-------------------------------------------------------------------AAAAAAAAAAAAAAAAAA----GATCGGAAGAGCACACG
160: AGTGTTCTCATATAAATAAAATAAATAAATCTTTGT--------------------------------------------------------------------AAAAAAAAAAAAAAAAA----AACCGGAAAAGCAAACG
161: AGTGTTCTCATATAAATAAAATAAATAAATCTTTGT-------------------------------------------------------------------AAAAAAAAAAAAAAAAAA----GATCGGAA-AGCAACCG
162: AGTGTTCTCATATAAATAAAATAAATAAATCTTTGT--------------------------------------------------------------------AAAAAAAAAAAAAAAAA----GACGGGAAAACCACACG
163: AGTGTTCTCATATAAATAAAATAAATAAATCTTTGT-------------------------------------------------------------------AAAAAAAAAAAAAAAAAA----GAACCGGAGGGCACCCC
164: AGTGTTCTCATATAAATAAAATAAATAAATCTTTGTGT-----------------------------------------------------------------AAAAAAAAAAAAAAAAAA----GATCGGACGAGCACACG
165: AGTGTTCTCATATAAATAAAATAAATAAATCTTTGTGT-------------------------------------------------------------------AAAAAAAAAAAAAAAA----GATCGGAAGAGCACACG
166: AGTGTTCTCATATAAATAAAATAAATAAATCTTTGTGT-------------------------------------------------------------------AAAAAAAAAAAAAAAA----GATCGGAAGAGCACACG
167: AGTGTTCTCATATAAATAAAATAAATAAATCTTTGTGT-------------------------------------------------------------------AAAAAAAAAAAAAAAA----GATCGGAAGAGCACACG
168: AGTGTTCTCATATAAATAAAATAAATAAATCTTTGTGT------------------------------------------------AAAAAAAAAAAAAAAAAAAAAAAAAAAAAAAAAAA----AAACGGAAAAACACACA
169: AGTGTTCTCATATAAATAAAATAAATAAATCTTTGTGT-----------------------------------------------AAAAAAAAAAAAAAAAAAAAAAAAAAAAAAAAAAAA----AACCGGAGACAACCACT
170: AGTGTTCTCATATAAATAAAATAAATAAATCTTTGTGTTT----------------------------------------------------AAAAAAAAAAAAAAAAAAAAAAAAAAAAT----GACCGAATAACTACACT
171: AGTGTTCTCATATAAATAAAATAAATAAATCTTTGTGTGC----------------------------------------AAAAAAAAAAAAAAAAAAAAAAAAAAAAAAAAAAAAAAAAA------CCGAAAAAAACAAAT
```

```
172: AGTGTTCTCATATAAATAAAATAAATAAATCTTTGTGTGCC-------------------------------------------------------------------AAAAAAAAAAAAA----AGATCGAAGAGCACACG
173: AGTGTTCTCATATAAATAAAATAAATAAATCTTTGTGTGCC------------------------------------------------------------------AAAAAAAAAAAAAA----GATCGGAAGAGCACACG
174: AGTGTTCTCATATAAATAAAATAAATAAATCTTTGTGTGTCCTC-----------------------------------------------AAAAAAAAAAAAAAAAAAAAAAAAAAAAAA----AAACGGAAAAGCAAACC
175: AGTGTTCTCATATAAATAAAATAAATAAATCTTTGTGTGCCCTCTGT-----------------------------------------------------------AAAAAAAAAAAAAAA----GATCGGAAGAGCACACG
176: AGTGTTCTCATATAAATAAAATAAATAAATCTTTGTGTGCCCTCTGTG----------------------------------------------AAAAAAAAAAAAAAAAAAAAAAAAAAA----AATCGGAAAAGCAACTC
177: AGTGTTCTCATATAAATAAAATAAATAAATCTTTGTGTGCCCTCTGTGGACTGTT-----------------------------AAAAAAAAAAAAAAAAAAAAAAAAAAAAAAAAAAAAA----AAAGGAAAAAGCAAAAA
178: AGTGTTCTCATATAAATAAAATAAATAAATCTTTGTGTGCCCTCTGTGGACTGTT----------------------------------------------------AAAAAAAAAAAAAA----GATCGGACGAGCACACG
179: AGTGTTCTCATATAAATAAAATAAATAAATCTTTGTGTGCCCTCTGTGGACTGTTCTCCCCTTGAGCTGGGCTTTCTCAGCCAAGCT---------------AAAAAAAAAAAAAAAAAAA----GATCGGAAAAGCAAACG
180: AGTGTTCTCATATAAATAAAATAAATAAATCTTTGTGTGCCCTCTGTGGACTGTTCTCCCCTTGAGCTGGGCTTTCTCAGCCAAGCTCGTTTTT------------AAAAAAAAAAAAAAA----GATCGGAAAA--AAACG
181: AGTGTTCTCATATAAATAAAATAAATAAATCTTTGTGTGCCCTCTGTGGACTGTTCTCCCCTTGAGCTGGGCTTTCTCAGCCAAGCTCGTTTT------------AAAAAAAAAAAAAAAA----GATCGGAAGAGCACACG
182: AGTGTTCTCATATAAATAAAATAAATAAATCTTTGTGTGCCCTCTGTGGACTGTTCTCCCCTTGAGCTGGGCTTTCTCAGCCAAGCTCGTTT--------------AAAAAAAAAAAAAAA----GATCGGAAGAGAACACG
183: AGTGTTCTCATATAAATAAAATAAATAAATCTTTGTGTGCCCTCTGTGGACTGTTCTCCCCTTGAGCTGGGCTTTCTCAGCCAAGCTCGTT--------------AAAAAAAAAAAAAAAA----AATCGGAAGAACAAACG
184: AGTGTTCTCATATAAATAAAATAAATAAATCTTTGTGTGCCCTCTGTGGACTGTTCTCCCCTTGAGCTGGGCTTTCTCAGCCAAGCTCGTTTTT----AAAAAAAAAAAAAAAAAAAAAAA----AATCGGAAAAGCACACG
185: AGTGTTCTCATATAAATAAAATAAATAAATCTTTGTGTGCCCTCTGTGGACTGTTCTCCCCTTGAGCTGGGCTTTCTCAGCCAAGCTCGTTTTTT-AAAAAAAAAAAAAAAAAAAAAAAAA----AAACGAAAAAGAAAACG
186: AGTGTTCTCATATAAATAAAATAAATAAATCTTTGTGTGCCCTCTGTGGACTGTTCTCCCCTTGAGCTGGGCTTTCTCAGCCAAGCTCGTTT------------AAAAAAAAAAAAAAAAA----AACCGAAAAAGAAAACA
187: AGTGTTCTCATATAAATAAAATAAATAAATCTTTGTGTGCCCTCTGTGGACTGTTCTCCCCTTGAGCTGGGCTTTCTCAGCCAAGCTCGTT-AAAAAAAAAAAAAAAAAAAAAAAAAAAAA----AAACGGAAAAGCACACC
188: AGTGTTCTCATATAAATAAAATAAATAAATCTTTGTGTGCCCTCTGTGGACTGTTCTCCCCTTGAGCTGGGCTTTCTCAGCCAAGCTCGTTT-------AAAAAAAAAAAAAAAAAAAAAA----AAATCGAAAAAAACACT
189: AGTGTTCTCATATAAATAAAATAAATAAATCTTTGTGTGCCCTCTGTGGACTGTTCTCCCCTTGAGCTGGGCTTTCTCAGCCAAGCTCGTTTTAAAAAAAAAAAAAAAAAAAAAAAAAAAAAAAAAAATCGAAAAAAAACAA
190: AGTGTTCTCATATAAATAAAATAAATAAATCTTTGTGTGCCCTCTGTGGACTGTTCTCCCCTTGAGCTGGGCTTTCTCAGCCAAGCTCGTTTTAAAAAAAAAAAAAAAAAAAAAAAAAAAA----AAAAAAAAAACAAAAAA
191: AGTGTTCTCATATAAATAAAATAAATAAATCTTTGTGTGCCCTCTGTGGACTGTTCTCCCCTTGAGCTGGGCTTTCTCAGCCAAGCTCGTTTAAAAAAAAAAAAAAAAAAAAAAAAAAAAA----AAAAAAAAAAAAAAGAA
192: AGTGTTCTCATATAAATAAAATAAATAAATCTTTGTGTGCCCTCTGTGGACTGTTCTCCCCTTGAGCTGGGCTTTCTCAGCCAAGCTCGTTTTAAAAAAAAAAAAAAAAAAAAAAAAAAAA----AAAAAAAAAAAAAAAAA
193: AGTGTTCTCATATAAATAAAATAAATAAATCTTTGTGTGCCCTCTGTGGACTGTTCTCCCCTTGAGCTGGGCTTTCTCAGCCAATCTCGTTTAAAAAAAAAAAAAAAAAAAAAAAAAAAAA----AAAAAAAAAAAAAAAAA
194: AGTGTTCTCATATAAATAAAATAAATAAATCTTTGTGTGCCCTCTGTGGACTGTTCTCCCCTTGAGCTGGGCTTTCTCAGCCAAGCTCGTTTAAAAAAAAAAAAAAAAAAAAAAAAAAAAA----AAAAAAAAAAAAAAACA
195: AGTGTTCTCATATAAATAAAATAAATAAATCTTTGTGTGCCCTCTGTGGACTGTTCTCCCCTTGAGCTGGGCTTTCTCAGCCAAGCTCGTTAAAAAAAAAAAAAAAAAAAAAAAAAAAAAA----AAAAAAACAAAAAACAA
196: AGTGTTCTCATATAAATAAAATAAATAAATCTTTGTGTGCCCTCTGTGGACTGTTCTCCCCTTGAGCTGGGCTTTCTCAGCCAAGCTCGTTTTAAAAAAAAAAAAAAAAAAAAAAAAAAAA----AAAAAACAAAAAACAAA
197: AGTGTTCTCATATAAATAAAATAAATAAATCTTTGTGTGCCCTCTGTGGACTGTTCTCCCCTTGAGCTGGGCTTTCTCAGCCAAGCTCGTTTTTAAAAAAAAAAAAAAAAAAAAAAAAAAA----AAAAACAAAAAAAAAAC
198: AGTGTTCTCATATAAATAAAATAAATAAATCTTTGTGTGCCCTCTGTGGACTGTTCTCCCCTTGAGCTGGGCTTTCTCAGCCAAGCTCGTTTTTAAAAAAAAAAAAAAAAAAAAAAAAAAA----AAACGGAAGAACACACA
199: AGTGTTCTCATATAAATAAAATAAATAAATCTTTGTGTGCCCTCTGTGGACTGTTCTCCCCTTGAGCTGGGCTTTCTCAGCCAAGCTCGTTTTTAAAAAAAAAAAAAAAAAAAAAAAAAAA----AATCGAAAGAACACAAA
200: AGTGTTCTCATATAAATAAAATAAATAAATCTTTGTGTGCCCTCTGTGGACTGTTCTCCCCTTGAGCTGGGCTTTCTCAGCCAAGCTCGTTTTAAAAAAAAAAAAAAAAAAAAAAACAAAA------------AAACACACG
Chr: AGTGTTCTCATATAAATAAAATAAATAAATCTTTGTGTGCCCTCTGTGGACTGTTCTCCCCTTGAGCTGGGCTTTCTCAGCCAAGCTCGTTTTTGGCCCCAGGACTCTGCTGCAGGGGCGGACAGGATGGA--------------
        3'-end region of B2       TCTTT                                                       TTTTT      poly(A)                   adapter
                               B2 terminator                                              random terminator
```

Figure S2. The nucleotide sequences of 200 reads were obtained from the sequencing of the seminal cDNA library and mapped to the genomic B2 copy (Chr15:82218345–82218558) in the mouse genome (copy 31 in Table 2). The 3'-terminal region is shown for the reads and the B2 genomic copy is highlighted in yellow. The position of the B2 terminator, a random terminator in the downstream sequence, and poly(A) are marked at the bottom. The shortened terminator sequences in the reads are highlighted in green.
