# Supplementary material for: Analysis of RNA Transcribed by RNA Polymerase III from B2 SINEs in Mouse Cells
Source: Noncoding RNA. 2025 May 14;11(3):39. doi: 10.3390/ncrna11030039 (PMC12101331; doi:10.3390/ncrna11030039)
Supplement: Supplementary file 1 [file ncrna-11-00039-s001.zip › ncrna-3586305-supplementary/Figure S3.pdf]

```
 1: GTACTCAAATACATAAATAAATAAATGTTT----------------AAAAAAAAAAAAAAAAAAAAAAAAAAAAAAAAAAAAAAAAAAAAAAAAAAAAATACAA
 2: GTACTCAAATACATAAATAAATAAATGTTT------------AAAAAAAAAAAAAAAAAAAAAAAAAAAAAAAAAAAAAAAAAAAAAAAAAAAAAAAAAAACAA
 3: GTACTCAAATACATAAATAAATAAATGTTT--------------AAAAAAAAAAAAAAAAAAAAAAAAAAAAAAAAAAAAAAAAAAAAAAAAAAAAAAAAACAA
 4: GTACTCAAATACATAAATAAATAAATGTTT-AAAAAAAAAAAAAAAAAAAAAAAAAAAAAAAAAAAAAAAAAAAAAAAAAAAAAAAAAAAAAAAAAAAAAAAAAACAA
 5: GTACTCAAATACATAAATAAATAAATGTT---AAAAAAAAAAAAAAAAAAAAAAAAAAAAAAAAAAAAAAAAAAAAAAAAAAAAAAAAAAAAAAAAAAAAAAAAACAA
 6: GTACTCAAATACATAAATAAATAAATGTTT-------AAAAAAAAAAAAAAAAAAAAAAAAAAAAAAAAAAAAAAAAAAAAAAAAAAAAAAAAAAAAAAAAAAA
 7: GTACTCAAATACATAAATAAATAAATGTTT-----------AAAAAAAAAAAAAAAAAAAAAAAAAAAAAAAAAAAAAAAAAAAAAAAAAAAAAAAAAAACCAG
 8: GTACTCAAATACATAAATAAATAAATGTTT------------------------------AAAAAAAAAAAAAAAAAAAAAAAAAAAAAAAAAAAAAAAAAACG
 9: GTACTCAAATACATAAATAAATAAATGTT-------------------------------AAAAAAAAAAAAAAAAAAAAAAAAAAAAAAAAAAAAAAAAACAG
10: GTACTCAAATACATAAATAAATAAATGTTT---------------------------------------------------AAAAAAAAAAAAAAAAAAAACAG
11: GTACTCAAATACATAAATAAATAAATGTTT-----------------------------------AAAAAAAAAAAAAAAAAAAAAAAAAAAAAAAAAAAAACG
12: GTACTCAAATACATAAATAAATAAATGTTT-------------------------------AAAAAAAAAAAAAAAAAAAAAAAAAAAAAAAAAAAAAAAAACG
13: GTACTCAAATACATAAATAAATAAATGTTT-----------------------------------AAAAAAAAAAAAAAAAAAAAAAAAAAAAAAAAAAAAAAG
14: GTACTCAAATACATAAATAAATAAATGTTT-----------------------------------AAAAAAAAAAAAAAAAAAAAAAAAAAAAAAAAAAGATCA
15: GTACTCAAATACATAAATAAATAAATGTTT----------------------------------AAAAAAAAAAAAAAAAAAAAAAAAAAAAAAAAAAAAAATG
16: GTACTCAAATACATAAATAAATAAATGTTT------------------------------------------AAAAAAAAAAAAAAAAAAAAAAAAAAAAAACG
17: GTACTCAAATACATAAATAAATAAATGTT------------------------------------------AAAAAAAAAAAAAAAAAAAAAAAAAAAAGATCG
18: GTACTCAAATACATAAATAAATAAATGTTT---------------------------------------AAAAAAAAAAAAAAAAAAAAAAAAAAAAAAAACAC
19: GTACTCAAATACATAAATAAATAAATGTTT----------------------------------------AAAAAAAAAAAAAAAAAAAAAAAAAAAAAGACCG
20: GTACTCAAATACATAAATAAATAAATGTT-------------------------------------------AAAAAAAAAAAAAAAAAAAAAAAAAAAAAACG
21: GTACTCAAATACATAAATAAATAAATGTTT-----------------------------------------AAAAAAAAAAAAAAAAAAAAAAAAAAAAAAACG
22: GTACTCAAATACATAAATAAATAAATGTTT-----------------------------------------AAAAAAAAAAAAAAAAAAAAAAAAAAAAGACCG
23: GTACTCAAATACATAAATAAATAAATGTTT----------------------------------------AAAAAAAAAAAAAAAAAAAAAAAAAAAAAAACCG
24: GTACTCAAATACATAAATAAATAAATGTTT----------------------------------------AAAAAAAAAAAAAAAAAAAAAAAAAAAAAAATCG
25: GTACTCAAATACATAAATAAATAAATGTTT------------------------------------AAAAAAAAAAAAAAAAAAAAAAAAAAAAAAAAAGAACG
26: GTACTCAAATACATAAATAAATAAATGTTT------------------------------------------AAAAAAAAAAAAAAAAAAAAAAAAAAAAACAG
27: GTACTCAAATACATAAATAAATAAATGTTT---------------------------------------AAAAAAAAAAAAAAAAAAAAAAAAAAAAAAAACAC
28: GTACTCAAATACATAAATAAATAAATGTTT--------------------------------------AAAAAAAAAAAAAAAAAAAAAAAAAAAAAAAAATCG
29: GTACTCAAATACATAAATAAATAAATGTTT-----------------------------------------------------AAAAAAAAAAAAAAAAGATCG
30: GTACTCAAATACATAAATAAATAAATGTT-------------------------------------------------------AAAAAAAAAAAAAAAAAACG
31: GTACTCAAATACATAAATAAATAAATGTTT----------------------------------------------------AAAAAAAAAAAAAAAAAGATCG
32: GTACTCAAATACATAAATAAATAAATGTT----------------------------------------------------AAAAAAAAAAAAAAAAAAGATCG
33: GTACTCAAATACATAAATAAATAAATGTTT----------------------------------------------------AAAAAAAAAAAAAAAAAGATCG
34: GTACTCAAATACATAAATAAATAAATGTTT----------------------------------------------------AAAAAAAAAAAAAAAAAGATCG
35: GTACTCAAATACATAAATAAATAAATGTTT-----------------------------------------------------AAAAAAAAAAAAAAAAGATCG
36: GTACTCAAATACATAAATAAATAAATGTT-----------------------------------------------------AAAAAAAAAAAAAAAAAGATCG
37: GTACTCAAATACATAAATAAATAAATGTTT-------------------------------------------------------AAAAAAAAAAAAAAGATCG
38: GTACTCAAATACATAAATAAATAAATGTTT-------------------------------------------------------AAAAAAAAAAAAAAGATCG
39: GTACTCAAATACATAAATAAATAAATGTTT-----------------------------------------------------AAAAAAAAAAAAAAAAGATCG
40: GTACTCAAATACATAAATAAATAAATGTTT-------------------------------------------------AAAAAAAAAAAAAAAAAAAAGATCG
41: GTACTCAAATACATAAATAAATAAATGTTT-------------------------------------------------AAAAAAAAAAAAAAAAAAAAGATCG
42: GTACTCAAATACATAAATAAATAAATGTTT----------------------------------------------------AAAAAAAAAAAAAAAAAGATCG
43: GTACTCAAATACATAAATAAATAAATGTTT---------------------------------------------------AAAAAAAAAAAAAAAAAAGATCG
44: GTACTCAAATACATAAATAAATAAATGTTT---------------------------------------------------AAAAAAAAAAAAAAAAAAGATCG
45: GTACTCAAATACATAAATAAATAAATGTTT-----------------------------------------------------AAAAAAAAAAAAAAAAGATCG
46: GTACTCAAATACATAAATAAATAAATGTTT-----------------------------------------------------AAAAAAAAAAAAAAAAGATCG
47: GTACTCAAATACATAAATAAATAAATGTTT-----------------------------------------------------AAAAAAAAAAAAAAAAGATCG
48: GTACTCAAATACATAAATAAATAAATGTTT-----------------------------------------------------AAAAAAAAAAAAAAAAGATCG
49: GTACTCAAATACATAAATAAATAAATGTT------------------------------------------------------AAAAAAAAAAAAAAAAGATCG
50: GTACTCAAATACATAAATAAATAAATGTTT--------------------------------------------------AAAAAAAAAAAAAAAAAAAGATCG
51: GTACTCAAATACATAAATAAATAAATGTTT------------------------------------------------------AAAAAAAAAAAAAAAGATCG
52: GTACTCAAATACATAAATAAATAAATGTT-------------------------------------------------------AAAAAAAAAAAAAAAGATCG
53: GTACTCAAATACATAAATAAATAAATGTTT-----------------------------------------------------AAAAAAAAAAAAAAAAGATCG
54: GTACTCAAATACATAAATAAATAAATGTTT---------------------------------------------------AAAAAAAAAAAAAAAAAAGATCG
55: GTACTCAAATACAGAAATAAATAAATGTTT---------------------------------------------------AAAAAAAAAAAAAAAAAAAAACG
56: GTACTCAAATACATAAATAAATAAATGTTT---------------------------------------------------AAAAAAAAAAAAAAAAAAGATCG
57: GTACTCAAATACATAAATAAATAAATGTTT--------------------------------------------------AAAAAAAAAAAAAAAAAAAGATCG
58: GTACTCAAATACATAAATAAATAAATGTTT---------------------------------------------------AAAAAAAAAAAAAAAAAAGATCG
59: GTACTCAAATACATAAATAAATAAATGTT-------------------------------------------------------AAAAAAAAAAAAAAAGATCG
60: GTACTCAAATACATAAATAAATAAATGTTT-----------------------------------------------------AAAAAAAAAAAAAAAAGATCG
61: GTACTCAAATACATAAATAAATAAATGTTT-----------------------------------------------------AAAAAAAAAAAAAAAAGATCG
62: GTACTCAAATACATAAATAAATAAATGTT-----------------------------------------------------AAAAAAAAAAAAAAAAAGATCG
63: GTACTCAAATACATAAATAAATAAATGTTT-----------------------------------------------------AAAAAAAAAAAAAAAAGATCG
64: GTACTCAAATACATAAATAAATAAATGTTT-----------------------------------------------------AAAAAAAAAAAAAAAAGATCG
65: GTACTCAAATACATAAATAAATAAATGTTT----------------------------------------------------AAAAAAAAAAAAAAAAAGATCG
66: GTACTCAAATACATAAATAAATAAATGTT----------------------------------------------------AAAAAAAAAAAAAAAAAAGATCG
67: GTACTCAAATACATAAATAAATAAATGTTT------------------------------------------------------AAAAAAAAAAAAAAAGATCG
68: GTACTCAAATACATAAATAAATAAATGTTT----------------------------------------------AAAAAAAAAAAAAAAAAAAAAAAGATCG
69: GTACTCAAATACATAAATAAATAAATGTTT----------------------------------------------AAAAAAAAAAAAAAAAAAAAAAAGATCG
70: GTACTCAAATACATAAATAAATAAATGTTT-------------------------------------------AAAAAAAAAAAAAAAAAAAAAAAAAAGATCG
71: GTACTCAAATACATAAATAAATAAATGTT--------------------------------------------------------AAAAAAAAAAAAAAGATCG
72: GTACTCAAATACATAAATAAATAAATGTT--------------------------------------------------------AAAAAAAAAAAAAAGATCG
73: GTACTCAAATACATAAATAAATAAATGTTT------------------------------------------------AAAAAAAAAAAAAAAAAAAAAGATCG
74: GTACTCAAATACATAAATAAATAAATGTTT------------------------------------------------AAAAAAAAAAAAAAAAAAAAAGATCG
75: GTACTCAAATACATAAATAAATAAATGTT-------------------------------------------------------AAAAAAAAAAAAAAAGATCG
76: GTACTCAAATACATAAATAAATAAATGTTT---------------------------------------------------AAAAAAAAAAAAAAAAAAGATCG
77: GTACTCAAATACATAAATAAATAAATGTTT-----------------------------------------------------AAAAAAAAAAAAAAAAGATCG
78: GTACTCAAATACATAAATAAATAAATGTT------------------------------------------------------AAAAAAAAAAAAAAAAGATCG
79: GTACTCAAATACATAAATAAATAAATGTTT--------------------------------------------AAAAAAAAAAAAAAAAAAAAAAAAAGATCG
80: GTACTCAAATACATAAATAAATAAATGTT----------------------------------------------AAAAAAAAAAAAAAAAAAAAAAAAAATCG
81: GTACTCAAATACATAAATAAATAAATGTTT----------------------------------------------------AAAAAAAAAAAAAAAAAGAACG
82: GTACTCAAATACATAAATAAATAAATGTTT-----------------------------------------------------AAAAAAAAAAAAAAAAGATCG
```

```
 83: GTACTCAAATACATAAATAAATAAATGTTT-------------------------------------------------------AAAAAAAAAAAAAAGAACG
 84: GTACTCAAATACATAAATAAATAAATGTTT-----------------------------------------------------AAAAAAAAAAAAAAAAAATCG
 85: GTACTCAAATACATAAATAAATAAATGTTT-----------------------------------------------------AAAAAAAAAAAAAAAAGATCG
 86: GTACTCAAATACATAAATAAATAAATGTTT--------------------------------------------------AAAAAAAAAAAAAAAAAAAAACGG
 87: GTACTCAAATACATAAATAAATAAATGTTT-------------------------------------------------AAAAAAAAAAAAAAAAAAAAAAACC
 88: GTACTCAAATACATAAATAAATAAATGTTT--------------------------------------------------AAAAAAAAAAAAAAAAAAAGATCG
 89: GTACTCAAATACATAAATAAATAAATGTTT---------------------------------------------------AAAAAAAAAAAAAAAAAAAATCG
 90: GTACTCAAATACATAAATAAATAAATGTTT---------------------------------------------------AAAAAAAAAAAAAAAAAAGATCG
 91: GTACTCAAATACATAAATAAATAAATGTT--------------------------------------------------AAAAAAAAAAAAAAAAAAAAGATCG
 92: GTACTCAAATACATAAATAAATAAATGTTT--------------------------------------------------AAAAAAAAAAAAAAAAAAAGATCG
 93: GTACTCAAATACATAAATAAATAAATGTTT-------------------------------------------------AAAAAAAAAAAAAAAAAAAAAAACG
 94: GTACTCAAATACATAAATAAATAAATGTT-----------------------------------------------------AAAAAAAAAAAAAAAAAGATCG
 95: GTACTCAAATACATAAATAAATAAATGTTT----------------------------------------------------AAAAAAAAAAAAAAAAAGATCG
 96: GTACTCAAATACATAAATAAATAAATGTTT----------------------------------------------------AAAAAAAAAAAAAAAAAGATCG
 97: GTACTCAAATACATAAATAAATAAATGTTT------------------------------------------------AAAAAAAAAAAAAAAAAAAAAGACCG
 98: GTACTCAAATACATAAATAAATAAATGTTT----------------------------AAAAAAAAAAAAAAAAAAAAAAAAAAAAAAAAAAAAAAAAAAACAC
 99: GTACTCAAATACATAAATAAATAAATGTTT------------------------------------AAAAAAAAAAAAAAAAAAAAAAAAAAAAAAAAAAAACC
100: GTACTCAAATACATAAATAAATAAATGTTT------------------------------------AAAAAAAAAAAAAAAAAAAAAAAAAAAAAAAAAGAACG
101: GTACTCAAATACATAAATAAATAAATGTTT-------------------AAAAAAAAAAAAAAAAAAAAAAAAAAAAAAAAAAAAAAAAAAAAAAAAAACCTCA
102: GTACTCAAATACATAAATAAATAAATGTTT----------------------------------------AAAATAAAAAAAAAAAAAAAAAAAAAAAAAAACG
103: GTACTCAAATACATAAATAAATAAATGTTT------------------------------------------AAAAAAAAAAAAAAAAAAAAAAAAAAAAAAAG
104: GTACTCAAATACATAAATAAATAAATGTTT-----------------------------------------AAAAAAAAAAAAAAAAAAAAAAAAAAAACATCG
105: GTACTCAAATACATAAATAAATAAATGTTT--------------------------------------------------AAAAAAAAAAAAAAAAAAAAACAG
106: GTACTCAAATACATAAATAAATAAATGTTT-----------------------------------------------------AAAAAAAAAAAAAAAAGATAG
107: GTACTCAAATACATAAATAAATAAATGTTT-----------------------------------------------------AAAAAAAAAAAAAAAAAACGG
108: GTACTCAAATACATAAATAAATAAATGTTT-----------------------------------------------------AAAAAAAAAAAAAAAAAAACG
109: GTACTCAAATACATAAATAAATAAATGTT-----------------------------------------------AAAAAAAAAAAAAAAAAAAAAAAGACAG
110: GTACTCAAATACATAAATAAATAAATGT------------------------------------------------------AAAAAAAAAAAAAAAAAGACCG
111: GTACTCAAATACATAAATAAATAAATGTTT------------------------------------------------------AAAAAAAAAAAAAAAAATCG
112: GTACTCAAATACATAAATAAATAAATGTT-------------------------------------------------------AAAAAAAAAAAAAAAGTCCG
113: GTACTCAAATACATAAATAAATAAATGTTT---------------------------------------------AAAAAAAAAAAAAAAAAAAAAAAAGATCG
114: GTACTCAAATACATAAATAAATAAATGTTT---------------------------------------------------AAAAAAAAAAAAAAAAAAAAACG
115: GTACTCAAATACATAAATAAATAAATGTTT----------------------------------------------AAAAAAAAAAAAAAAAAAAAAAAGACCT
116: GTACTCAAATACATAAATAAATAAATGTTT----------------------------------AAAAAAAAAAAAAAAAAAAAAAAAAAAAAAAAACAGAAAA
117: GTACTCAAATACATAAATAAATAAATGTTT----------------------------------------------------AAAAAAAAAAAAAAAAAGATCG
118: GTACTCAAATACATAAATAAATAAATGTTT----------------------------------------------------AAAAAAAAAAAAAAAAAAAACG
119: GTACTCAAATACATAAATAAATAAATGT---------------------------------------------------------AAAAAAAAAAAAAAAAAAG
120: GTACTCAAATACATAAATAAATAAATGTTT---------------------------------------------------AAAAAAAAAAAAAAAAAAGATCG
121: GTACTCAAATACATAAATAAATAAATGTTT----------------------------------------------------AAAAAAAAAAAAAAAAAGATCG
122: GTACTCAAATACATAAATAAATAAATGTTT--------------------------------------------AAAAAAAAAAAAAAAAAAAAAAAAAAACCG
123: GTACTCAAATACATAAATAAATAAATGTT-----------------------------------------------------AAAAAAAAAAAAAAAAAGTTTG
124: GTACTCAAATACATAAATAAATAAATGTTT------------------------------------------------------AAAAAAAAAAAAAAAAAACG
125: GTACTCAAATACATAAATAAATAAATGTTTG-----------------------------------------------------AAAAAAAAAAAAAAAGATCG
126: GTACTCAAATACATAAATAAATAAATGTTTG----------------------------------------AAAAAAAAAAAAAAAAAAAAAAAAAAAAAACCG
127: GTACTCAAATACATAAATAAATAAATGTTTG---------------------------------------------------AAAAAAAAAAAAAAAAAGATCG
128: GTACTCAAATACATAAATAAATAAATGTTTG--------------------------------------------------AAAAAAAAAAAAAAAAAAGATCG
129: GTACTCAAATACATAAATAAATAAATGTTTGCTT-------------------------------------------------AAAAAAAAAAAAAAAAGATCG
130: GTACTCAAATACATAAATAAATAAATGTTTGCTTC--------------------------------------------------AAAAAAAAAAAAAAAATAG
131: GTACTCAAATACATAAATAAATAAATGTTTGCTTC------------------------------------------------AAAAAAAAAAAAAAAAGATCG
132: GTACTCAAATACATAAATAAATAAATGTTTGCTTC------------------------------------------------AAAAAAAAAAAAAAAAAATGG
133: GTACTCAAATACATAAATAAATAAATGTTTGCTTC------------------------------------------------AAAAAAAAAAAAAAAAGATCG
134: GTACTCAAATACATAAATAAATAAATGTTTGCTTC----------------------------------------------AAAAAAAAAAAAAAAAAAAAACG
135: GTACTCAAATACATAAATAAATAAATGTTTGCTTC-------------------------------------AAAAAAAAAAAAAAAAAAAAAAAAAAAGAAAA
136: GTACTCAAATACATAAATAAATAAATGTTTGCTTC----------------------------------------AAAAAAAAAAAAAAAAAAAAAAAAAAAAG
137: GTACTCAAATACATAAATAAATAAATGTTTGCTTC---------------------------------------------AAAAAAAAAAAAAAAAAAAGATCG
138: GTACTCAAATACATAAATAAATAAATGTTTGCTTC--------------------------------------------------AAAAAAAAAAAAAAGATCG
139: GTACTCAAATACATAAATAAATAAATGTTTGCTTC------------------------------------------------AAAAAAAAAAAAAAAAGATCG
140: GTACTCAAATACATAAATAAATAAATGTTTGCTTC-------------------------------------------------AAAAAAAAAAAAAAAGACCG
141: GTACTCAAATACATAAATAAATAAATGTTTGCTTC--------------------------------------------AAAAAAAAAAAAAAAAAAAAGACCG
142: GTACTCAAATACATAAATAAATAAATGTTTGCTTCAAGC----------------------------------------AAAAAAAAAAAAAAAAAAAAGATCG
143: GTACTCAAATACATAAATAAATAAATGTTTGCTTCAAGC---------------------------AAAAAAAAAAAAAAAAAAAAAAAAAAAAAAAAAAACAG
144: GTACTCAAATACATAAATAAATAAATGTTTGCTTCAAGC----------------------------------------AAAAAAAAAAAAAAAAAAAAAAACG
145: GTACTCAAATACATAAATAAAAAAATGTTTGCTTCAAGC-----------------------------AAAAAAAAAAAAAAAAAAAAAAAAAAAAAAAAATCG
146: GTACTCAAATACATAAATAAATAAATGTTTGCTTCAAGCAGCTGTTGGAATCAC----------------------------AAAAAAAAAAAAAAAAAGATCG
147: GTACTCAAATACATAAATAAATAAATGTTTGCTTCAAGCAGCTGTTGGAATCACTTT---------------------------AAAAAAAAAAAAAAAGATCG
148: GTACTCAAATACATAAATAAATAAATGTTTGCTTCAAGCAGCTGTTGGAATCACTTT--------------------------AAAAAAAAAAAAAAAAGATCG
149: GTACTCAAATACATAAATAAATAAATGTTTGCTTCAAGCAGCTGTTGGAATCACTTT--------------------------AAAAAAAAAAAAAAAAGATCG
150: GTACTCAAATACATAAATAAATAAATGTTTGCTTCAAGCAGCTGTTGGAATCACTTT-------------------------AAAAAAAAAAAAAAAAAGATCG
151: GTACTCAAATACATAAATAAATAAATGTTTGCTTCAAGCAGCTGTTGGAATCACTTT-------------------------AAAAAAAAAAAAAAAAAGATCG
152: GTACTCAAATACATAAATAAATAAATGTTTGCTTCAAGCAGCTGTTGGAATCACTTT-----------------------AAAAAAAAAAAAAAAAAAAGATCG
153: GTACTCAAATACATAAATAAATAAATGTTTGCTTCAAGCAGCTGTTGGAATCACTTT---------------AAAAAAAAAAAAAAAAAAAAAAAAAAAAATCG
154: GTACTCAAATACATAAATAAATAAATGTTTGCTTCAAGCAGCTGTTGGAATCACTTT---------------------AAAAAAAAAAAAAAAAAAAAAAAATG
155: GTACTCAAATACATAAATAAATAAATGTTTGCTTCAAGCAGCTGTTGGAATCACTTT-----------------AAAAAAAAAAAAAAAAAAAAAAAAAGATCG
156: GTACTCAAATACATAAATAAATAAATGTTTGCTTCAAGCAGCTGTTGGAATCACTTT-------------AAAAAAAAAAAAAAAAAAAAAAAAAAAAAAATCA
157: GTACTCAAATACATAAATAAATAAATGTTTGCTTCAAGCAGCTGTTGGAATCACTTT---------------------AAAAAAAAAAAAAAAAAAAAAGAACG
158: GTACTCAAATACATAAATAAATAAATGTTTGCTTCAAGCAGCTGTTGGAATCACTTT------------------------AAAAAAAAAAAAAAAAAAAAACG
159: GTACTCAAATACATAAATAAATAAATGTTTGCTTCAAGCAGCTGTTGGAATCACT----------------------------AAAAAAAAAAAAAAAAGATCG
160: GTACTCAAATACATAAATAAATAAATGTTTGCTTCAAGCAGATGTTGGAATCACTTT--------------------AAAAAAAAAAAAAAAAAAAAAAGATCG
161: GTACTCAAATACATAAATAAATAAATGTTTGCTTCAAGCAGCTGTTGGAATCACTTT----------------------AAAAAAAAAAAAAAAAAAAAACCTC
162: GTACTCAAATACATAAATAAATAAATGTTTGCTTCAAGCAGCTGTTGGAATCACTTT----------------------AAAAAAAAAAAAAAAAAAAAATCCC
163: GTACTCAAATACATAAATAAATAAATGTTTGCTTCAAGCAGCTGTTGGAATCACTTT-----------AAAAAAAAAAAAAAAAAAAAAAAAAAAAAAAAATCG
164: GTACTCAAATACATAAATAAATAAATGTTTGCTTCAAGCAGCTGTTGGAATCACTTAAAAAAAAAAAAAAAAAAAAAAAAAAAAAAAAAAAAAAAAAAAAACCAAA
165: GTACTCAAATACATAAATAAATAAATGTTTGCTTCAAGCAGCTGTTGGAATCACTTAAAAAAAAAAAAAAAAAAAAAAAAAAAAAAAAAAAAAAAAAAAAAAAAAA
166: GTACTCAAATACATAAATAAATAAATGTTTGCTTCAAGCAGCTGTTGGAATCACTTTAAAAAAAAAAAAAAAAAAAAAAAAAAAAAAAAAAAAAAAAAAAAAA
```

```
167: GTACTCAAATACATAAATAAATAAATGTTTGCTTCAAGCAGCTGTTGGAATCACTTTT---------------------AAAAAAAAAAAAAAAAAAAATAACG
168: GTACTCAAATACATAAATAAATAAATGTTTGCTTCAAGCAGCTGTTGGAATCACTTTT------------------------AAAAAAAAAAAAAAAAAGATCG
169: GTACTCAAATACATAAATAAATAAATGTTTGCTTCAAGCAGCTGTTGGAATCACTTTT--------------------AAAAAAAAAAAAAAAAAAAAAGATCG
170: GTACTCAAATACATAAATAAATAAATGTTTGCTTCAAGCAGCTGTTGGAATCACTTTT----------------AAAAAAAAAAAAAAAAAAAAAAAAAACTTA
171: GTACTCAAATACATAAATAAATAAATGTTTGCTTCAAGCAGCTGTTGGAATCACTTTTAAAAAAAAAAAAAAAAAAAAAAAAAAAAAAAAAAAAAAAAAAAAAC
172: GTACTCAAATACATAAATAAATAAATGTTTGCTTCAAGCAGCTGATGGAATCACTTTTAAAAAAAAAAAAACAAAAAAAAAAAAAAAAAAAAAAAAAAAAAAAA
Chr: GTACTCAAATACATAAATAAATAAATGTTTGCTTCAAGCAGCTGTTGGAATCACTTTTAAAAAAACCATCAATCTAGTATGTATGTATTTATGTGTATG-----
         3′-end of B2          TGTTT                        TTTT                           poly(A)         adapter
                         B2 terminator            random terminator
```

Figure S3. The nucleotide sequences of 172 reads were obtained from the sequencing of the seminal cDNA library and mapped to the genomic B2 copy (chr4:44554747–44554923 in the mouse genome (copy 32 in Table 2). The 3'-terminal region is shown and the B2 genomic copy is highlighted in yellow. The position of the B2 terminator, a random terminator in the downstream sequence, and poly(A) are marked at the bottom. The shortened terminator sequences in the reads are highlighted in green.
