# Supplementary material for: Analysis of RNA Transcribed by RNA Polymerase III from B2 SINEs in Mouse Cells
Source: Noncoding RNA. 2025 May 14;11(3):39. doi: 10.3390/ncrna11030039 (PMC12101331; doi:10.3390/ncrna11030039)
Supplement: Supplementary file 1 [file ncrna-11-00039-s001.zip › ncrna-3586305-supplementary/Figure S4.pdf]

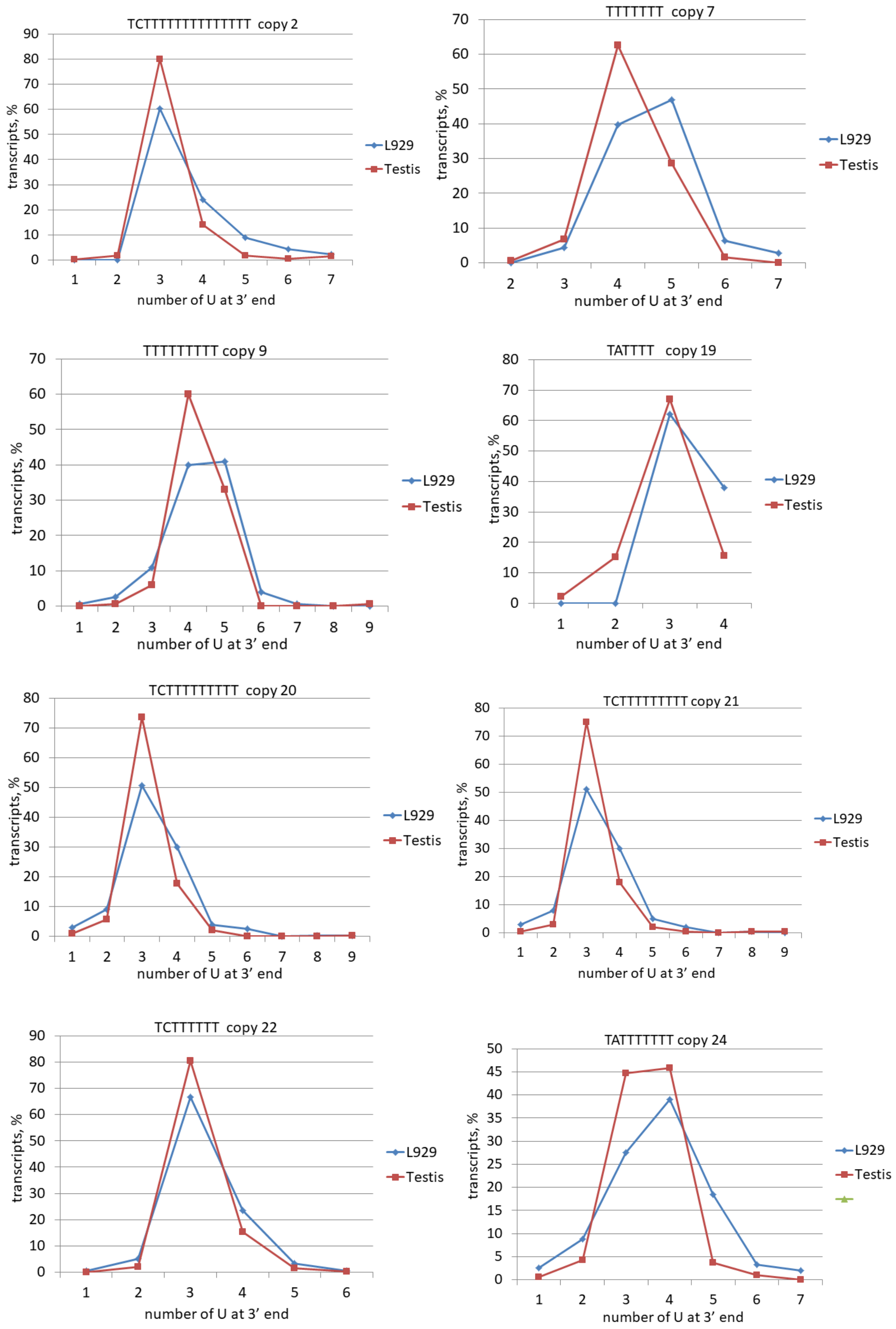

Figure S4. Comparison of transcriptional arrest at terminators of individual B2 copies in L929 cells and testis. B2 copy numbers (see Tables 1 and 2) and their terminator sequences are indicated above the graphs. The number of U residues present at the 3'-ends of transcripts (reads) is indicated on the X-axis. The graphs show the proportion of transcripts of a given B2 copy of stopped at a particular terminator T residue.
