# Supplementary material for: Analysis of RNA Transcribed by RNA Polymerase III from B2 SINEs in Mouse Cells
Source: Noncoding RNA. 2025 May 14;11(3):39. doi: 10.3390/ncrna11030039 (PMC12101331; doi:10.3390/ncrna11030039)
Supplement: Supplementary file 1 [file ncrna-11-00039-s001.zip › ncrna-3586305-supplementary/Figure S5.pdf]

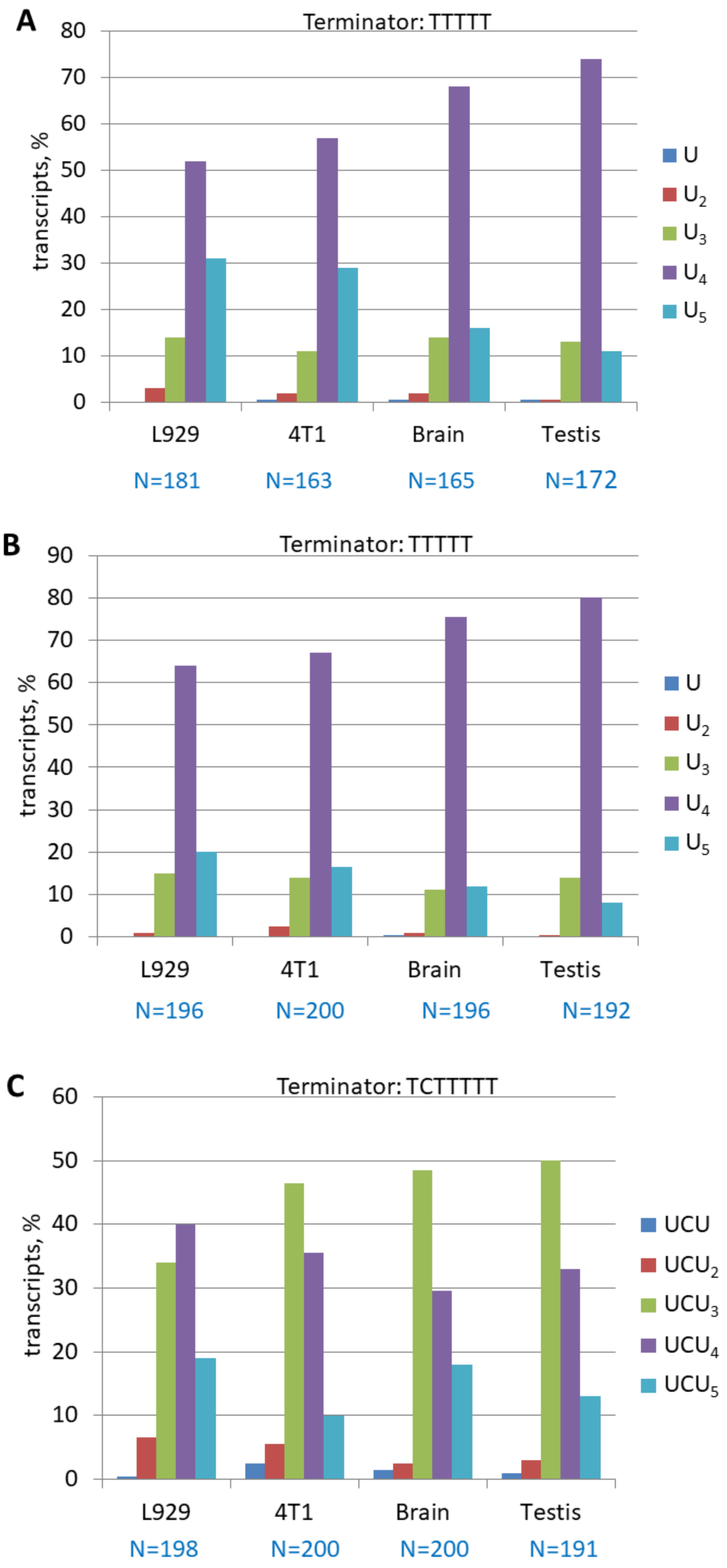
A
Terminator: TTTTT
transcripts, %
0
10
20
30
40
50
60
70
80
U
U2
U3
U4
U5
L929
4T1
Brain
Testis
N=181
N=163
N=165
N=172
B
Terminator: TTTTT
transcripts, %
0
10
20
30
40
50
60
70
80
90
U
U2
U3
U4
U5
L929
4T1
Brain
Testis
N=196
N=200
N=196
N=192
C
Terminator: TCTTTTT
transcripts, %
0
10
20
30
40
50
60
UCU
UCU2
UCU3
UCU4
UCU5
L929
4T1
Brain
Testis
N=198
N=200
N=200
N=191

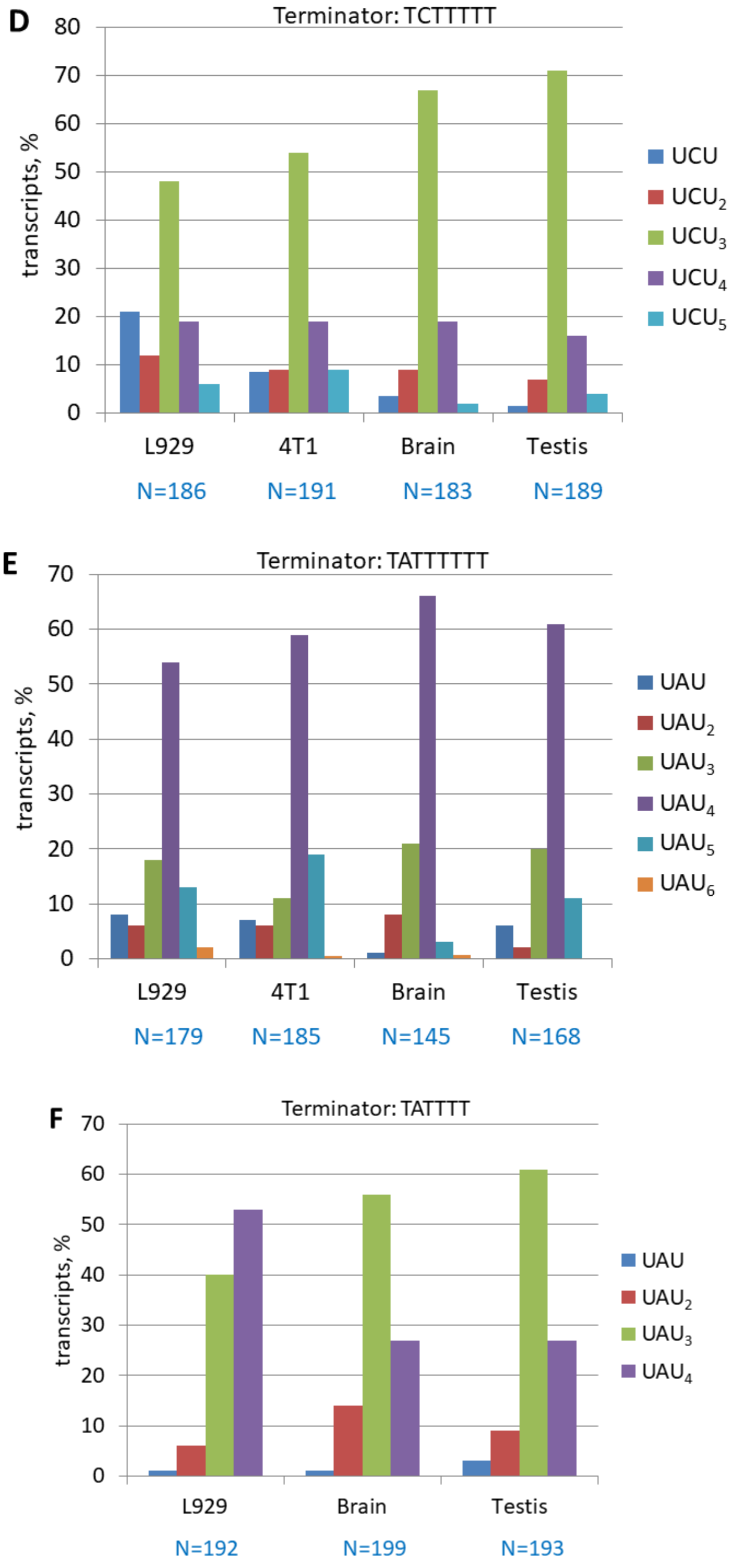

Figure S5. Comparison of transcription arrest at terminators of individual B2 copies in L929, 4T1 cells, brain, and testis. The diagrams illustrate the proportion of transcripts of a given B2 copy that are stopped at a particular terminator position. The coordinates of B2 copies in the mouse genome are chr12:70914253–70914643 (**A**), chr8:122560895–122561046 (**B**), chr12:78556332–78556501 (**C**), chr10:71303131–71303280 (**D**), chr2:153730677–153730853 (**E**), and chr1:133036101–133036282 (**F**). The number of analyzed reads in each library is indicated below as N = number.
