# Supplementary material for: Analysis of RNA Transcribed by RNA Polymerase III from B2 SINEs in Mouse Cells
Source: Noncoding RNA. 2025 May 14;11(3):39. doi: 10.3390/ncrna11030039 (PMC12101331; doi:10.3390/ncrna11030039)
Supplement: Supplementary file 1 [file ncrna-11-00039-s001.zip › ncrna-3586305-supplementary/Figure S6.pdf]

A. B2 copy coordinates: chr4:135922401-135922589

```
              10        20        30        40        50        60        70        80        90
 1. : TGTACTCATACATATAAGTAATATAAATAAACAAATATT-AAAAAAAAAAAAAAG-AAAACGAGCAGGTGCTTGTGTCTGCTTTTAAAAAAAAAAAAAAA
 2. : TGTACTCATACATATAAGTAATATAAATAAACAAATATT-AAAAAAAAAAAAAAG-AAAACGAGCAGGTGCTTGTGTCTGCTTTTAAAAAAAAAAAAAAA
 3. : TGTACTCATACATATAAGTAATATAAATAAACAAATATT-AAAAAAAAAAAAAAG-AAAACGAGCAGGTGCTTGTGTCTGCTTTTAAAAAAAAAAAAAAA
 4. : TGTACTCATACATATAAGTAATATAAATAAACAAATATT-----AAAAAAAAAAG-AAAACGAGCAGGTGCTTGTGTCTGCTTTTTAAAAAAAAAAAAAA
 5. : TGTACTCATACATATAAGTAATATAAATAAACAAATATT-----AAAAAAAAAAG-AAAACGAGCAGGTGCTTGTGTCTGCTTTTTAAAAAAAAAAAAAA
 6. : TGTACTCATACATATAAGTAATATAAATAAACAAATATT-----AAAAAAAAAAG-AAAACGAGCAGGTGCTTGTGTCTGCTTTTAAAAAAAAAAAAAAA
 7. : TGTACTCATACATATAAGTAATATAAATAAACAAATATT-----AAAAAAAAAAG-AAAACGAGCAGGTGCTTGTGTCTGCTTTTAAAAAAAAAAAAAAA
 8. : TGTACTCATACATATAAGTAATATAAATAAACAAATATT-----AAAAAAAAAAG-AAAACGAGCAGGTGCTTGTGTCTGCTTTTAAAAAAAAAAAAAAA
 9. : TGTACTCATACATATAAGTAATATAAATAAACAAATATT-----AAAAAAAAAAGAAAAACGAGCAGGTGCTTGTGTCTGCTTTTAAAAAAAAAAAAAAA
10. : TGTACTCATACATATAAGTAATATAAATAAACAAATATT------AAAAAAAAAG-AAAACGAGCAGGTGCTTGTGTCTGCTTTTAAAAAAAAAAAAAAA
11. : TGTACTCATACATATAAGTAATATAAATAAACAAATATT---AAAAAAAAAAAAG-AAAACGAGCAGGTGCTTGTGTCTGCTTTTAAAAAAAAAAAAAAA
12. : TGTACTCATACATATAAGTAATATAAATAAACAAATATT---AAAAAAAAAAAAG-AAAACGAGCAGGTGCTTGTGTCTGCTTTTAAAAAAAAAAAAAAA
13. : TGTACTCATACATATAAGTAATATAAATAAACAAATATT---AAAAAAAAAAAAG-AAAACGAGCAGGTGCTTGTGTCTGCTTTTAAAAAAAAAAAAAAA
14. : TGTACTCATACATATAAGTAATATAAATAAACAAATATT------AAAAAAAAAG-AAAACGAGCAGGTGCTTGTGTCTGCTTTTAAAAAAAAAAAAAAA
15. : TGTACTCATACATATAAGTAATATAAATAAACAAATATT----AAAAAAAAAAAG-AAAACGAGCAGGTGCTTGTGTCTGCTTTTAAAAAAAAAAAAAAA
16. : TGTACTCATACATATAAGTAATATAAATAAACAAATATT----AAAAAAAAAAAG-AAAACGAGCAGGTGCTTGTGTCTGCTTTTAAAAAAAAAAAAAAA
17. : TGTACTCATACATATAAGTAATATAAATAAACAAATATT----AAAAAAAAAAAG-AAAACGAGCAGGTGCTTGTGTCTGCTTTTAAAAAAAAAAAAAAA
18. : TGTACTCATACATATAAGTAATATAAATAAACAAATATT----AAAAAAAAAAAG-AAAACGAGCAGGTGCTTGTGTCTGCTTTTAAAAAAAAAAAAAAA
19. : TGTACTCATACATATAAGTAATATAAATAAACAAATATT---AAAAAAAAAAAAG-AAAACGAGCAGGTGCTTGTGTCTGCTTTTAAAAAAAAAAAAAAA
20. : TGTACTCATACATATAAGTAATATAAATAAACAAATATT---AAAAAAAAAAAAG-AAAACGAGCAGGTGCTTGTGTCTGCTTTTAAAAAAAAAAAAAAA
21. : TGTACTCATACATATAAGTAATATAAATAAACAAATATT-----AAAAAAAAAAG-AAAACGAGCAGGTGCTTGTGTCTGCTTTTAAAAAAAAAAAAAAA
22. : TGTACTCATACATATAAGTAATATAAATAAACAAATATT-----AAAAAAAAAAG-AAAACGAGCAGGTGCTTGTGTCTGCTTTTAAAAAAAAAAAAAAA
23. : TGTACTCATACATATAAGTAATATAAATAAACAAATATT-----AAAAAAAAAAG-AAAACGAGCAGGTGCTTGTGTCTGCTTTAAAAAAAAAAAAAAAA
24. : TGTACTCATACATATAAGTAATATAAATAAACAAATATT---AAAAAAAAAAAAG-AAAACGAGAAGGTGCTTGTGTCTGCTTTTAAAAAAAAAAAAAAA
25. : TGTACTCATACATATAAGTAATATAAATAAACAAATATT----AAAAAAAAAAAG-AAAACGAGCAGGTGCTTGTGTCTGCTTTTTAAAAAAAAAAAAAA
26. : TGTACTCATACATATAAGTAATATAAATAAACAAATATT----AAAAAAAAAAAG-AAAACGAGCAGGTGCTTGTGTCTGCTTTTTAAAAAAAAAAAAAA
27. : TGTACTCATACATATAAGTAATATAAATAAACAAATATT----AAAAAAAAAAAG-AAAACGAGCAGGTGCTTGTGTCTGCTTTTAAAAAAAAAAAAAAA
28. : TGTACTCATACATATAAGTAATATAAATAAACAAATATT----AAAAAAAAAAAG-AAAACGAGCAGGTGCTTGTGTCTGCTTTTTAAAAAAAAAAAAAA
29. : TGTACTCATACATATAAGTAATATAAATAAACAAATATT------AAAAAAAAAG-AAAACGAGCAGGTGCTTGTGTCTGCTTTTAAAAAAAAAAAAAAA
30. : TGTACTCATACATATAAGTAATATAAATAAACAAATATT-----AAAAAAAAAAG-AAAACGAGCAGGTGCTTGTGTCTGCTTTTAAAAAAAAAAAAAAA
31. : TGTACTCATACATATAAGTAATATAAATAAACAAATATT-----AAAAAAAAAAG-AAAACGAGCAGGTGCTTGTGTCTGCTTTTAAAAAAAAAAAAAAA
32. : TGTACTCATACATATAAGTAATATAAATAAACAAATATT----AAAAAAAAAAAG-AAAACGAGCAGGTGCTTGTGTCTGCTTTTAAAAAAAAAAAAAAA
33. : TGTACTCATACATATAAGTAATATAAATAAACAAATATT-----AAAAAAAAAAG-AAAACGAGCAGGTGCTTGTGTCTGCTTTTAAAAAAAAAAAAAAA
34. : TGTACTCATACATATAAGTAATATAAATAAACAAATATT---AAAAAAAAAAAAG-AAAACGAGCAGGTGCTTGTGTCTGCTTTTAAAAAAAAAAAAAAA
35. : TGTACTCATACATATAAGTAATATAAATAAACAAATATT--AAAAAAAAAAAAAG-AAAACGAGCAGGTGCTTGTGTCTGCTTTTAAAAAAAAAAAAAAA
36. : TGTACTCATACATATAAGTAATATAAATAAACAAATATT-----AAAAAAAAAAG-AAAACGAGCAGGTGCTTGTGTCTGCTTTTAAAAAAAAAAAAAAA
37. : TGTACTCATACATATAAGTAATATAAATAAACAAATATT-----AAAAAAAAAAG-AAAACGAGCAGGTGCTTGTGTCTGCTTTTTAAAAAAAAAAAAAA
38. : TGTACTCATACATATAAGTAATATAAATAAACAAATATTAAAAAAAAAAAAAAAG-AAAACGAGCAGGTGCTTGTGTCTGCTTTAAAAAAAAAAAAAAAA
39. : TGTACTCATACATATAAGTAATATAAATAAACAAATATTAAAAAAAAAAAAACAG-AAAACGAGCAGGTGCTTGTGTCTGCTAAAAAAAAAAAAAAAACA
40. : TGTACTCATACATATAAGTAATATAAATAAACAAATATT-----AAAAAAAAAAG-AAAACGAGCAGGTGCTTGTGTCTGCTTTTAAAAAAAAAAAAAAA
41. : TGTACTCATACATATAAGTAATATAAATAAACAAATATT------AAAAAAAAAG-AAAACGAGCAGGTGCTTGTGTCTGCTTTTAAAAAAAAAAAAAAA
42. : TGTACTCATACATATAAGTAATATAAATAAACAAATATT------AAAAAAAAAG-AAAACGAGCAGGTGCTTGTGTCTGCTTTAAAAAAAAAAAAAAAA
43. : TGTACTCATACATATAAGTAATACAAATAAACAAATATT------AAAAAAAAAG-AAAACGAGCAGGTGCTTGTGTCTGCTTTAAAAAAAAAAAAAAAA
44. : TGTACTCATACATATAAGTACTATAAATAAACAAATATT----AAAAAAAAAAAG-AAAACGAGCAGGTGCTTGTGTCTGCTTTTAAAAAAAAAAAAAAA
45. : TGTACTCATACATATAAGTAATATAAATAAACAAATATT-----AAAAAAAAAAG-AAAACGAGCAGGTGCTTGTGTCTGCTTTTAAAAAAAAAAAAAAA
46. : TGTACTCATACATATAAGTAATATAAATAAACAAATATT-AAAAAAAAAAAACAG-AAAACGAGCAGGTGCTTGTGTCTGCTTTAAAAAAAAAAAAAAAA
47. : TGTACTCATACATATAAGTAATATAAATAAACAAATATTAAAAAAAAAAAAAAAG-AAAACGAGCAGGGGCTTGTGTCTGCTTTTAAAAAAAAAAAAAAA
48. : TGTACTCATACATATAAGTAATATAAATAAACAAATATT-----AAAAAAAAAAG-AAAACGAGCAGGTGCTTGTGTCTGCTTTTAAAAAAAAAAAAAAA
49. : TGTACTCATACATATAAGTAATATAAATAAACAAATATT---AAAAAAAAAAAAG-AAAACGAGCAGGTGCTTGTGTCTGCTTTAAAAAAAAAAAAAAAA
50. : TGTACTCATACATATAAGTAATATAAATAAACAAATATT-AAAAAAAAAAAAAAG-AAAACGAGCAGGTGCTTGTGTCTGCTTAAAAAAAAAAAAAAAAA
51. : TGTACTCATACATATAAGTAATATAAATAAACAAATATT------AAAAAAAAAG-AAAACGAGCAGGTGCTTGTGTCTGCTTTAAAAAAAAAAAAAAA-
52. : TGTACTCATACATATAAGTAATATAAATAAACAAATATT---AAAAAAAAAAAAG-AAAACGAGCAGGTGCTTGTGTCTGCTTTAAAAAAAAAAAAAAAA
53. : TGTACTCATACATATAAGTAATATAAATAAACAAATATT--AAAAAAAAAAAAAG-AAAACGAGCAGGTGCTTGTGTCTGCTTTTAAAAAAAAAAAAAAA
```

```
54. : TGTACTCATACATATAAGTAATATAAATAAACAAATATT-AAAAAAAAAAAAAAG-AAAACGAGCAGGTCCTTGTGTCTGCTTTTAAAAAAAAAAAAAA
55. : TGTACTCATACATATAAGTAATATAAATAAACAAATATT---AAAAAAAAAAAAGAAAAACGAGCAGGTGATTGTGTCTGCTTTTAAAAAAAAAAAAAA
56. : TGTACTCATACATATAAGTAATATAAATAAACAAATATT--AAAAAAAAAAAAAG-AAAACGAGCAGGTGCTTGTGTCTGCTTTTAAAAAAAAAAAAAA
57. : TGTACTCATACATATAAGTAATATAAATAAACAAATATT----AAAAAAAAAAAG-AAAACGAGCAGGTGCTTGTGTCTGCTTTTAAAAAAAAAAAAAA
58. : TGTACTCATACATATAAGTAATATAAATCAACAAATATT------AAAAAAAAAG-AAAACGAGCAGGGGCTTGTGTCTGCTTTAAAAAAAAAAAAAAA
59. : TGTACTCATACATATAAGTAATATAAATAAACAAATATT----AAAAAAAAAAAG-AAAACGAGCCGGTGCTTGTGTCTGCTTTTAAAAAAAAAAAAAA
60. : TGTACTCATACATATAAGTAATATAAATAAACAAATATT----AAAAAAAAAAAG-AAAACGAGCAGGTGCTTGTGTCTGCTTAAAAAAAAAAAAAAAA
61. : TGTACTCATACATATAAGTAATATAAATAAACAAATATT------AAAAAAAAAG-AAAACGAGCAGGTGCTTGTGTCTGCTTTTAAAAAAAAAAAAAA
62. : TGTACTCATACATATAAGTAATATAAATAAACAACTATT----AAAAAAAAAAAG-AAAACGAGCAGGTGCTTGTGTCTGCTTTTAAAAAAAAAAAAAA
Chr4: TGTACTCATACATATAAGTAATATAAATAAACAAATATT-----AAAAAAAAAAG-AAAACGAGCAGGTGCTTGTGTCTGCTTTTGCTCTTGCCAATAG
          3'-end region of B2            TATT                                       TTTTT    poly(A)
                                      B2 rudimentary                              Downstream
                                      terminator                                  terminator
```

The distance between rudimentary and downstream terminators is 36 bp

B. B2 copy coordinates: chr4:32588562-32588729

```
                    *        20         *        40         *        60         *        80         *       100         *       120         *       140
  1.: TGTACTTACATATAATAAAATAAATAAAGCTT---AAAAAAAAAAGAGTGCAA-AAACCTTATCCTT-AAAGCGTTCACAAGCTGGTTAAGCAGGGTTGTATAATAAATCAATCATCATTTTAAAAAAAAAAAAAAAAGA
  2.: TGTACTTACATATAATAAAATAAATAAAGCTT----AAAAAAAAAGAGTGCAC-AAACCTTATCCTT-AAAGCGTTCACAAGCTGGTTAAGCAGGGTTGTATAATAAATCAATCATCATTTTAAAAAAAAAAAAAAAAAA
  3.: TGTACTTACATATAATAAAATAAATAAAGCTT----AAAAAAAAAAAGTGCAC-AAACCTTATCCTT-AAAGCGTTCACAAGCTGGTTAAGCAGGGTTGTATAATAAATCAATCATCATTTTAAAAAAAAAAAAAAAAAA
  4.: TGTACTTACATATAATAAAATAAATAAAGCTT--AAAAAAAAAAAGAGTGCAC-AAACCTTATCCTT-AAAGCGTTCACAAGCTGGTTAAGCAGGGTTGTATAATAAATCAATCATCATTTTTAAAAAAAAAAAAAAAAA
  5.: TGTACTTACATATAATAAAATAAATAAAGCTT----AAAAAAAAAGAGTGCAC-AAACCTTATCCTT-AAAGCGTTCACAAGCTGGTTAAGCAGGGTTGTATAATAAATCAATCATCATTTTTTAAAAAAAAAAAAAAAA
  6.: TGTACTTACATATAATAAAATAAATAAAGCTT----AAAAAAAAAGAGTGCAC-AAACCTTATCCTT-AAAGCGTTCACAAGCTGGTTAAGCAGGGTTGTATAATAAATCAATCATCATTTTAAAAAAAAAAAAAAAAAA
  7.: TGTACTTACATATAATAAAATAAATAAAGCTT----AAAAAAAAAGAGTGCAC-AAACCTTATCCTT-AAAGCGTTCACAAGCTGGTTAAGCAGGGTTGTATAATAAATCAATCATCATTTTAAAAAAAAAAAAAAAAAA
  8.: TGTACTTACATATAATAAAATAAATAAAGCTT----AAAAAAAAAGAGTGCAC-AAACCTTATCCTT-AAAGCGTTCACAAGCTGGTTAAGCAGGGTTGTATAATAAATCAATCATCATTTTAAAAAAAAAAAAAAAAAA
  9.: TGTACTTACATATAATAAAATAAATAAAGCTT----AAAAAAAAAGAGTGCAC-AAACCTTAACCTT-AAAGCGTTCACAAGCTGGTTAAGCAGGGTTGTATAATAAATCAATCATCATTTTAAAAAAAAAAAAAAAAAA
 10.: TGTACTTACATATAATAAAATAAATAAAGCTT----AAAAAAAAAGAGTGCAC-AAACCTTATCCTT-AAAGCGTTCACAAGCTGGTTAAGCAGGGTTGTATAATAAATCAATCATCATTTTAAAAAAAAAAAAAAAAAA
 11.: TGTACTTACATATAATAAAATAAATAAAGCTT----AAAAAAAAAGAGTGCAC-AAACCTTATCCTT-AAAGCGTTCACAAGCTGGTTAAGCAGGGTTGTATAATAAATCAATCATCATTTAAAAAAAAAAAAAAAAAAA
 12.: TGTACTTACATATAATAAAATAAATAAAGCTT----AAAAAAAAAGAGTACAC-AAACCTTATCCTT-AAAGCGTTCACAAGCTGGTTAAGCAGGGTTGTATAATAAATCAATCATCATTTTAAAAAAAAAAAAAAAAAA
 13.: TGTACTTACATATAATAGAATAAATAAAGCTT----AAAAAAAAAGAGTGCAC-AAACCTTATCCTT-AAAGCGTTCACAAGCTGGTTAAGCAGGGTTGTATAATAAATCAATCATCATTTTAAAAAAAAAAAAAAAAAA
 14.: TGTACTTACATATAATAAAATAAATAAAGCTT----AAAAAAAAAGAGTGCAC-AAACCTTATCCTT-AAAGCGTTCACAAGCTGGTTAAGCAGGGTTGTATAATAAATCAATCATCATTTTAAAAAAAAAAAAAAAAAA
 15.: TGTACTTACATATAATAAAATAAATAAAGCTT----AAAAAAAAAGAGTGCAC-AAACCTTATCCTT-AAAGCGTTCACAAGCTGGTTAAGCAGGGTTGTATAATAAATCAATCATCATTTTTAAAAAAAAAAAAAAAAA
 16.: TGTACTTACATATAATAAAATAAATAATGCTT----AAAAAAAAAAAGTGCAC-AAACCTTATCCTT-AACGCGTTCACAAGCTGGTTAAGCAGGGTTGTATAATAAATCAATCATCATTTTTAAAAAAAAAAAAAAAAA
 17.: TGTACTTACATATAATAAAATAAATAAAGCTT----AAAAAAAAAGAGTGCAC-AAACCTTATCCTT-AAAGCGTTCACAAGCTGGTTAAGCAGGGTTGTATAATAAATCAATCATCATTTTAAAAAAAAAAAAAAAAAA
 18.: TGTACTTACATATAATAAAATAAATAAAGCTT----AAAAAAAAAGAGTGCAC-AAACCTTATCCTT-AAAGCGTTCACAAGCTGGTTAAGCAGGGTTGTATAATAAATCAATCATCATTTTAAAAAAAAAAAAAAAAAA
 19.: TGTACTTACATATAATAAAATAAATAAAGCTT----AAAAAAAAAGAGTGCAC-AAACCTTATCCTT-AAAGCGTTCACAAGCTGGTTAAGCAGGGTTGTATAATAAATCAATCATCATTTAAAAAAAAAAAAAAAAAAA
 20.: TGTACTTACATATAATAAAATAAATAAAGCTT----AAAAAAAAAGAGTGCAC-AAACCTTATCCTT-AAAGCGTTCACAAGCTGGTTAAGCAGGGTTGTATAATAAATCAATCATCATTTTAAAAAAAAAAAAAAAAAA
 21.: TGTACTTACATATAATAAAATAAATAAAGCTT----AAAAAAAAAGAGTGCAC-AAACCTTATCCTT-AAAGCGTTCACAAGCTGGTTAAGCAGGGTTGTATAATAAATCAATCATCATTTTAAAAAAAAAAAAAAAAAA
 22.: TGTACTTACATATAATAAAATAAATAAAGCTT-----AAAAAAAAGAGTGCAC-AAACCTTATCCTT-AAAGCGTTCACAAGCTGGTTAAGCAGGGTTGTATAATAAATCAATCATCATTTTAAAAAAAAAAAAAAAA--
 23.: TGTACTTACATATAATAAAATAAATAAAGCTT-----AAAAAAAAGAGTGCAC-AAACCTTATCCTT-AAAGCGTTCACAAGCTGGTTAAGCAGGGTTGTATAATAAATCAATCATCATTTTAAAAAAAAAAAAAAAAAA
 24.: TGTACTTACATATAATAAAATAAATAAAGCTT-----AAAAAAAAGAGTGCAC-AAACCTTATCCTT-AAAGCGTTCACAAGCTGGTTAAGCAGGGTTGTATAATAAATCAATCATCATTTAAAAAAAAAAAAAAGA---
 25.: TGTACTTACATATAATAAAATAAATAAAGCTT----AAAAAAAAAGAGTGCACAAACCTTATCCTT-AAAGCGTTCACAAGCTGGTTAAGCAGGGTTGTATAATAAATCAATCATCATTTAAAAAAAAAAAAAAAAAA-
 26.: TGTACTTACATATAATAAAATAAATAAAGCTT--AAAAAAAAAAAGAGTGCAC-AAACCTTATCCTT-AAAGCGTTCACAAGCTGGTTAAGCAGGGTTGTATAATAATTCAATCATCATTTTAAAAAAAAAAAAAAAAAA
 27.: TGTACTTACATATAATAAAATAAATAAAGCTT----AAAAAAAAAGAGTGCAC-AAACCTTATCCTT-AAAGCGTTCACAAGCTGGTTAAGCAGGGTTGTATAATAAATCAATCATCATTTAAAAAAAAAAAAAAAAAAA
 28.: TGTACTTACATATAATAAAATAAATAAAGCTT----AAAAAAAAAGAGTACAC-AAACCTTATCCTT-AAAGCGTTCACAAGCTGGTTAAGCAGGGTTGTATAATAAATCAATCATCATTTTAAAAAAAAAAAAAAAAAA
 29.: TGTACTTACATATAATAAAATAAATAAAGCTTAAAAAAAAAAAAAGAGTGCAC-AAACCTTATCCTT-AAAGCGTTCACAAGCTGGTTAAGCAGGGTTGTATAATAAATCAATCATCATTTAAAAAAAAAAAAAAAAAAA
 30.: TGTACTTACATATAATAAAATAAATAAAGCTTAAAAAAAAAAAAAGAGTGCCC-AAACCTTATCCTT-AAAGCGTTCACAAGCTGGTTAAGCAGGGTTGTATAATAAATCAATCATCATTTTAAAAAAAAAAAAAAAAAA
 31.: TGTACTTACATATAATAAAATAAATAAAGCTT----AAAAAAAAAGAGTGCAC-AAACCTTATCCTT-AAAGCGTTCACAAGCTGGTTAAGCAGGGTTGTATAATAAATCAATCATCATTAAAAAAAAAAAAAAAAAAAA
 32.: TGTACTTACATATAATAAAATAAATAAAGCTT----AAAAAAAAAGAGTGCAC-AAACCTTATCCTT-AAAGCGTTCACAAGCTGGTTAAGCAGGGTTGTATAATAAATCAATCATCATTTTAAAAAAAAAAAAAAAAAA
 33.: TGTACTTACATATAATAAAATAAATAAAGCTT----AAAAAAAAAGAGTGCAC-AAACCTTATCCTT-AAAGCGTTCACAAGCTGGTTAAGCAGGGTTGTATAATAAATCAATCATCATTTTAAAAAAAAAAAAAAAAAA
 34.: TGTACTTACATATAATAAAATAAATAAAGCTT----AAAAAAAAAGAGTGCAC-AAACCTTATCCTT-AAAGCGTTCACAAGCTGGTTAAGCAGGGTTGTATAATAAATCAATCATCATTTTAAAAAAAAAAAAAAAAAA
 35.: TGTACTTACATATAATAAAATAAATAAAGCTT----AAAAAAAAAGAGTTCAC-AAACCTTATCCNT-AAAGCGTTCACAAGCTGGTTAAGCAGGGTTGTATAATAAATCAATCATCATTTTAAAAAAAAAAAAAAAAAA
 36.: TGTACTTACATATAATAAAATAATTAAAGCTT----AAAAACAAAGAGTGCAC-AAACCTTATCCNT-AAAGCGTTCACAAGCTGGTTAAGCAGGGTTGTATAATAAATCAATCATCATTTTAAAAAAAAAAAAAAAAAA
```

```
37.: TGTACTTACATATAATAAAATAAATAAAGCTT---AAAAAAAAAAGAGTGCAC-AAACCTTATCCTT-AAAGCGTTCACAAGCTGGTTAAGCAGGGTTGTATAATAAATCAATCATCATTTTAAAAAAAAAAAAAAAAAA
38.: TGTACTTACATATAATAAAATAAATAAAGCTT---AAAAAAAAAAGAGTGCAC-AAACCTTATCCTT-AAAGCGTTCACAAGCTGGTTAAGCAGGGTTGTATAATAAATCAATCATCATTTTAAAAAAAAAAAAAAAAAA
39.: TGTACTTACATATAATAAAATAAATAAAGCTT---AAAAAAAAAAGAGTGCAC-AAACCTTATCCTT-AAAGCGTTCACAAGCTGGTTAAGCAGGGTTGTATAATAAATCAATCATCATTTTAAAAAAAAAAAAAAAAAC
40.: TGTACTTACATATAATAAAATAAATAAAGCTT---AAAAAAAAAAGAGTGCAC-AAACCTTATCCTT-AAAGCGTTCACAAGCTGGTTAAGCAGGGTTGTATAATAAATCAATCATCATTTTAAAAAAAAAAAAAAAAAA
41.: TGTACTTACATATAATAAAATAAATAAAGCTT----AAAAAAAAAGAGTGCAC-AAACCTTATCCTT-AAAGCGTTCACAAGCTGGTTAAGCAGGGTTGTATAATAAATCAATCATCATTTTAAAAAAAAAAAAAAAAAA
42.: TGTACTTACATACAATAAAATAAATAAAGCTTAAAAAAAAAAAAAGAGTGCAC-AAACCTTATCCTT-AAAGCGTTCACAAGCTGGTTAAGCAGGGTTGTATAATAAATCAATCATCATTAAAAAAAAAAAAAAAAAAAA
43.: TGTACTTACATATAATAAAATAAATAAAGCTT-----AAAAAAAAGAGTGCAC-AAACCTTAACCTT-AAAGCGTTCACAAGCTGGTTAAGCAGGGTTGTATAATACATCAATCATCATTTAAAAAAAAAAAAAAAAAAA
44.: TGTACTTACATATAATAAAATAAATAAAGCTT---AAAAAAAAAAGAGTGCAC-AAACCTTATCCTT-AAAGCGTTCACAAGCTGGTTAAGCAGGGTTGTATAATAAATCAATCATCATTTTTTAAAAAAAAAAAAAAAA
45.: TGTACTTACATATAATAAAATAAATAAAGCTT----AAAAAAAAAGAGTGCAC-AAACCTTATCCTT-AAAGCGTTCACAAGCTGGTTAAGCAGGGTTGTATAATAAATCAATCATCATTTTAAAAAAAAAAAAAAAAAA
46.: TGTACTTACATATAATAAAATAAATAAAGCTT-----AAAAAAAAGAGTGCAC-AAACCTTATCCTT-AAAGCGTTCACAAGCTGGTTAAGCAGGGTTGTATAATAAATCAATCATCATTTTAAAAAAAAAAAAAAAAAA
47.: TGTACTTACATATAATAAAATAAATAAAGCTT---AAAAAAAAAAGAGTGCAC-AAACCTTATCCTT-AAAGCGTTCACAAGCTGGTTAAGCAGGGTTGTATAATAAATCAATCATCATTTAAAAAAAAAAAAAAAAAAA
48.: TGTACTTACATATAATAAAATAAATAAAGCTT---AAAAAAAAAAGAGAGCAC-AAACCTTATCCTT-AAAGCGTTCACAAGCTGGTTAAGCAGGGTTGTATAATAAATCAATCCTCATTTTTAAAAAAAAAAAAAAAAA
49.: TGTACTTACATATAATAAAATAAATAAAGCTT----AAAAAAAAAGAGTGCAC-AAACCTTATCCTT-AAAGCGTTCACAAGCTGGTTAAGCAGGGTTGTATAATAAATCAATCATCATTTAAAAAAAAAAAAAAAAAAA
50.: TGTACTTACATATAATAAAATAAATAAAGCTT---AAAAAAAAAAGAGTGCAC-AAACCTTATCCTTAAAAGCGTTCACAAGCTGGTTAAGCAGGGTTGTATAATAAATCAATCATCATTTTTAAAAAAAAAAAAAAAAA
51.: TGTACTTACATATAATAAAATAAATAAAGCTTA-AAAAAAAAAAAGAGTGCAC-AAACCTTATCCTT-AAAGCGTTCACAAGCTGGTTAAGCAGGGTTGTATAATAAATCAATCATCATTTTTAAAAAAAAAAAAAAAAA
52.: TGTACTTACATATAATAAAATAAATAAAGCTTA-AAAAAAAAAAAGAGTGCAC-AAACCTTANCCTT-AAAGCGTTCACAAGCTGGTTAAGCAGGGTTGTATAATAAATCAATCATCATTTTTAAAAAAAAAAAAAAAAA
53.: TGTACTTACATATAATAAAATAAATAAAGCTT---AAAAAAAAAAGAGTGCAC-AAACCTTATCCTT-AAAGCGTTCACAAGCTGGTTAAGCAGGGTTGTATAATAAATCAATCATCATTTAAAAAAAAAAAAAAAAAAA
54.: TGTACTTACATATAATAAAATAAATAAAGCTT---AAAAAAAAAAGAGTGCAC-AAACCTTATCCTT-AAAGCGTTCACAAGCTGGTTAAGCAGGGTTGTATAATAAATCAATCATCATTAAAAAAAAAAAAAAAAAAAA
55.: TGTACTTACATATAATAAAATAAATAAAGCTT---AAAAAAAAAAGAGTGCAC-AAACCTTATCCTT-AAAGCGTTCACAAGCTGGTTAAGCAGGGTTGTATAATAAATCAATCATCATTAAAAAAAAAAAAAAAAAAAA
56.: TGTACTTACATATAATAAAATAAATAAAGCTT---AAAAAAAAAAGAGTGCAC-AAACCTTATCCTT-AAAGCGTTCACAAGCTGGTTAAGCAGGGTTGTATAATAAATCAATCATCATTTAAAAAAAAAAAAAAAAAAA
57.: TGTACTTACATATAATAAAATAAATAAAGCTT----AAAAAAAAAGAGTGCAC-AACCCTTATCCTT-AAAGCGTTCACAAGCTGGTTAAGCAGGGTTGTATAATAAATCAATCATCATTTTAAAAAAAAAAAAAAAAAA
58.: TGTACTTACATATAATAAAATAAATAAAGCTT---AAAAAAAAAAGAGAGCCC-AAACCTTATCCTT-AAAGCGTTCACAAGCTGGTTAAGCAGGGTTGTATAATAAATCAATCATCATTTTAAAAAAAAAAAAAAAAAA
59.: TGTACTTACATATAATAAAATAAATAAAGCTT----AAAAAAAAAGAGTGCTC-AAACCTTATCCTT-AAAGCGTTCACAAGCTGGTTAAGCAGGGTTGTATAATAAATCAATCATCATTTTTAAAAAAAAAAAAAAAAA
60.: TGTACTTACATATAATAAAATAAATAAAGCTA----AAAAAAAAAGAGTGCAC-AAACCTTATCCTT-AAAGCGTTCACAAGCTGGTTAAGCAGGGTTGTATAATAAATCAATCATCATTTTAAAAAAAAAAAAAAAAAA
61.: TGTACTTACATATAATAAAATAAATAAAGCTT---AAAAAAAAAAGAGTGCAC-AAACCTTATCCTT-AAAGCGTTCACAAGCTGGTTAAGCAGGGTTGTATAATAAATCAATCATCATTTTTAAAAAAAAAAAAAAAAA
62.: TGTACTTACATATAATAAAATAAATAAAGCTT----AAAAAAAAAGAGTGCAC-AAACCTTATCCTT-AAAGCGTTCACAAGCTGGTTAAGCAGGGTTGTATAATAAATCAATCATCATTTTAAAAAAAAAAAAAAAAAA
63.: TGTACTTACATATAATAAAATAAATAAAGCTT----AAAAAAAAAGAGTGCAC-AAACCTTATCCTT-AAAGCGTTCACAAGCTGGTTAAGCAGGGTTGTATAATAAATCAATCATCATTTTAAAAAAAAAAAAAAAAAA
64.: TGTACTTACATATAATAAAATAAATAAAGCTTAAAAAAAAAAAAAGAGTGCAC-AAACCTTAACCTT-AAAGCGTTCACAAGCTGGTTAAGCAGGGTTGTATAATAAATCAATCCTCATTTTAAAAAAAAAAAAAAAAAA
65.: TGTACTTACATATAATAAAATAAATAAAGCTT----AAAAAAAACGAGTGCACAAACCTTATCCTT-AGAGCGTTCACAAGCTGGTTAAGCAGGGTTGTATAATAAATCAATCATCATTTTAAAAAAAAAAAAAAAAAA
66.: TGTACTTACATATAATAAAATAAATAAAGCTT---AAAAAAAACAGAGTGCCC-AAACCTTATCCTTAAAAGCGTTCACAAGCTGGTTAAGCAGGGTTGTATAATAAATCAATCATCATTTTTAAAAAAAAAAAAAAAAA
67.: TGTACTTACATATAATAAAATAAATAAAGCTT---AAAAAAAAAAGAGTGCAC-AAACCTTATCCTTAAAAGCGTTCACAAGCTGGTTAAGCAGGGTTGTATAATAAATCAATCATCATTTTTAAAAAAAAAAAAAAAAG
68.: TGTACTTACATATACTAAAATAAATAAAGCTT-----AAAAAAAAGAGTGCAC-AAACCTTATCCTT-AAAGCGTTCACAAGCTGGTTAAGCAGGGTTGTCTAATAAATCAATCATCATTTTAAAAAAAAAAAAAAGAAA
Chr4: TGTACTTACATATAATAAAATAAATAAAGCTT----AAAAAAAAAGAGTGCAC-AAACCTTATCCTT-AAAGCGTTCACAAGCTGGTTAAGCAGGGTTGTATAATAAATCAATCATCATTTTTTCCAGGGCTTCTAACTC
        3’-end region of B2     TT                                                                                                 TTTTTT   poly(A)
                             B2 rudimentary                                                                                        Downstream
                                terminator                                                                                         terminator
```

The distance between rudimentary and downstream terminators is 82 bp

C. B2 copy coordinates: chr6:73015125_73015298.

```
                       *         20         *         40         *         60         *         80         *        100         *        120         *        140
 1. : TGTACTCACATATAAT---AAATAAATAAATAAATAAATAAAAAAATCTTT---AAAAAAATTTCATAGTCTGGTCTGATCTTGATAGACCCAATTGAGAAGAATTCTTGTTTAAAAAAAAAAAAAAAAAAAAAACGAAA
 2. : TGTACTCACATATAAT---AAATAAATAAATAAATAAATAAATAAATCTTT---AAAAAAATTTCATAGTCTGGTCTGATCTTGATAGACCCAATTGAGAAGAATTCTTGTTTAAAAAAAAAAAAAAAAAAAAAAACGAA
 3. : TGTACTCACATATAAT---AAATAAATAAATAAATAAATAAATAAATCTTT---AAAAAAATTTCATAGTCTGGTCTGATCTTGATAGACCCAATTGAGAAGAATTCTTGTTTAAAAAAAAAAAAAAAAAAAAAAATCAG
 4. : TGTACTCACATATAAT---AAATAAATAAATAAATAAATAAATAAATCTTT---AAAAAAATTTCATAGTCTGGTCTGATCTTGATAGACCCAATTGAGAAGAATTCTTGTTTTAAAAAAAAAAAAAAAAAAAAAAAAAA
 5. : TGTACTCACATATAAT---AAATAAATAAATAAATAAATAAATAAATCTTT---AAAAAAATTTCATAGTCTGGTCTGATCTTGATAGACCCAATTGAGAAGAATTCTTGTTTTAAAAAAAAAAAAAAAAAAAAAAAAAA
 6. : TGTACTCACATATAAT---AAATAAATAAATAAATAAATAAATAAATCTTT---AAAAAAATTTCATAGTCTGGTCTGATCTTGATAGACCCAATTGAGAAGAATTCTTGTTTAAAAAAAAAAAAAAAAAAAAAAAAAAA
 7. : TGTACTCACATATAAT---AAATAAATAAATAAATAAATAAATAAATCTTT--AAAAAAAATTTCATAGTCTGGTCTGATCTTGATAGACCCAATTGAGAAGAATTCTTGTTAAAAAAAAAAAAAAAAAAAAAAAAAAAA
 8. : TGTACTCACATATAAT---AAATAAATAAATAAATAAATAAATAAATCTTT--AAAAAAAATTTCATAGTCTGGTCTGATCTTGATAGACCCAATTGAGAAGAATTCTTGTTAAAAAAAAAAAAAAAAAAAAAAAAAAAA
 9. : TGTACTCACATATAAT---AAATAAATAAATAAATAAATAAATAAATCTTT--AAAAAAAATTTCATAGTCTGGTCTGATCTTGATAGACCCAATTGAGAAGAATTCTAAAAAAAAAAAAAAAAAAAAAAAAAAAAAAAA
10. : TGTACTCACATATAAT---AAATAAATAAATAAATAAATAAATAAATCTTT---AAAAAAATTTCATAGTCTGGTCTGATCTTGATAGACCCAATTGAGAAGAATTCTTAAAAAAAAAAAAAAAAAAAAAAAAAAAAAAA
11. : TGTACTCACATATAAT---AAATAAATAAATAAATAAATAAATAAATCTTT---AAAAAAATTTCATAGTCTGGTCTGATCTTGATAGACCCAATTGAGAAGAATTCTTAAAAAAAAAAAAAAAAAAAAAAAAAAAAAAA
12. : TGTACTCACATATAAT---AAATAAATAAATAAATAAATAAATAAATCTTT---AAAAAAATTTCATAGTCTGGTCTGATCTTGATAGACCCAATTGAGAAGAATTCTTGTTTTAAAAAAAAAAAAAAAAAAAAAAAAAA
13. : TGTACTCACATATAAT---AAATAAATGAATAAATAAATAAATAAATCTTT---AAAAAAATTTCATAGTCTGGTCTGATCTTGATAGACCCAATTGAGAAGAATTCTTGTTTAAAAAAAAAAAAAAAAAAAAAAAAAAA
14. : TGTACTCACATATAAT---AAATAAATAAATAAATAAATAAATAAATCTTTAAAAAAAAAATTTCATAGTCTGGTCTGATCTTGATAGACCCAATTGAGACGAATTCTTGTTTAAAAAAAAAAAAAAAAAAAAAAAAAAA
15. : TGTACTCACATATAAT---AAATAAATAAATAAATAAATAAATAAATCTTT---AAAAAAATTTCATAGTCTGGTCTGATCTTGATAGACCCAATTGAGAAGAATTCTTGTTTTAAAAAAAAAAAAAAAAAAAAAAAAAA
```

```
 16. : TGTACTCACATATAAT---AAATAAATAAATAAATAAATAAATAAATCTTT--AAAAAAAAATTTCATAGTCTGGTCTGATCTTGATAGACCCAATTGAGAAGAATTCTTGTTTAAAAAAAAAAAAAAAAAAAAAAAAAAA
 17. : TGTACTCACATATAAT---AAATAAATAAATAAATAAATAAATAAATCTTT---AAAAAAAATTTCATAGTCTGGTCTGATCTTGATAGACCCAATTGAGAAGAATTCTTGTTTAAAAAAAAAAAAAAAAAAAAAAAAAAA
 18. : TGTACTCACATATAAT---AAATAAATAAATAAATAAATAAATAAATCTTT---AAAAAAAATTTCATAGTCTGGTCTGATCTTGATAGACCCAATTGAGAAGAATTCTTGTTTTAAAAAAAAAAAAAAAAAAAAAAAAAA
 19. : TGTACTCACATATAAT---AAATAAATAAATAAATAAATAAATAAATCTTT---AAAAAAAATTTCATAGTCTGGTCTGATCTTGATAGACCCAATTGAGAAGAATTCTTGTTTAAAAAAAAAAAAAAAAAAAAAAAAAAA
 20. : TGTACTCACATATAAT---AAATAAATAAATAAATAAATAAATAAATCTTT---AAAAAAAATTTCATAGTCTGGTCTGATCTTGATAGACCCAATTGAGAAGAATTCTTGTTTAAAAAAAAAAAAAAAAAAAAAAAAAAC
 21. : TGTACTCACATATAAT---AAATAAATAAATAAATAAATAAATAAATCTTT---AAAAAAAATTTCATAGTCTGGTCTGATCTTGATAGACCCAATTGAGAAGAATTCTTGTTTAAAAAAAAAAAAAAAAAAAAAAAAAAA
 22. : TGTACTCACATATAAT---AAATAAATAAATAAATAAATAAATAAATCTTT---AAAAAAAATTTCATAGTCTGGTCTGATCTTGATAGACCCAATTGAGAAGAATTCTTGTTTTAAAAAAAAAAAAAAAAAAAAAAAAAA
 23. : TGTACTCACATATAAT---AAATAAATAAATAAATAAATAAATAAATCTTT---AAAAAAAATTTCATAGTCTGGTCTGATCTTGATAGACCCAATTGAGAAGAATTCTTGTTTAAAAAAAAAAAAAAAAAAAAAATCGGAA
 24. : TGTACTCACATATAAT---AAATAAATAAATAAATAAATAAATAAATCTTT---AAAAAAAATTTCATAGTCTGGTCTGATCTTGATAGACCCAATTGAGAAGAATTCTTGTTTAAAAAAAAAAAAAAAAAAAAAATCGGAA
 25. : TGTACTCACATATAAT---AAATAAATAAATAAATAAATAAATAAATCTTT---AAAAAAAATTTCATAGTCTGGTCTGATCTTGATAGACCCAATTGAGAAGAATTCTTGTTTAAAAAAAAAAAAAAAAAAAAAATCGGAA
 26. : TGTACTCACATATAATAAAAAATAAATAAATAAATAAATAAATAAATCTTT---AAAAAAAATTTCATAGTCTGGTCTGATCTTGATAGACCCAATTGAGAAGAATTCTTGTTTAAAAAAAAAAAAAAAAAAAAAATCGGAA
 27. : TGTACTCACATATAATAAAAAATAAATAAATAAATAAATAAATAAATCTTT---AAAAAAAATTTCATAGTCTGGTCTGATCTTGATAGACCCAATTGAGAAGAATTCTTGTTTAAAAAAAAAAAAAAAAAAAAAAACGGAA
 28. : TGTACTCACATATAAT---AAATAAATAAATAAATAAATAAATAAATCTTT---AAAAAAAATTTCATAGTCTGGTCTGATCTTGATAGACCCAATTGAGAAGAATTCTTGTTTTAAAAAAAAAAAAAAAAAAAAAGAAAGG
 29. : TGTACTCACATATAAT---AAATAAATAAATAAATAAATAAATAAATCTTT---AAAAAAAATTTCATAGTCTGGTCTGATCTTGATAGACCCAATTGAGAAGAATTCTTGTTTAAAAAAAAAAAAAAAAAAAAAAAAAGG
 30. : TGTACTCACATATAAT---AAATAAATAAATAAATAAATAAATAAATCTTT---AAAAAAAATTTCATAGTCTGGTCTGATCTTGATAGACCCAATTGAGAAGAATTCTTGTTTTAAAAAAAAAAAAAAAAAAAAAAAAGG
 31. : TGTACTCACATATAAT---AAATAAATAAATAAATAAATAAATAAATCTTT---AAAAAAAATTTCATAGTCTGGTCTGATCTTGATAGACCCAATTGAGAAGAATTCTTGTTTTAAAAAAAAAAAAAAAAAAAAAAAAAA
 32. : TGTACTCACATATAAT---AAATAAATAAATAAATAAATAAATAAATCTTT---AAAAAAAATTTCATAGTCTGGTCTGATCTTGATAGACCCAATTGAGAAGAATTCTTGTTTAAAAAAAAAAAAAAAAAAATCGGAAGAG
 33. : TGTACTCACATATAAT---AAATAAATAAATAAATAAATAAATAAATCTTT---AAAAAAAATTTCATAGTCTGGTCTGATCTTGATAGACCCAATTGAGAAGAATTCTTGTTTAAAAAAAAAAAAAAAAAAATCGGAAAAA
 34. : TGTACTCACATATAAT---AAATAAATAAATAAATAAATAAATAAATCTTT---AAAAAAAATTTCATAGTCTGGTCTGATCTTGATCGACCCAATTGCGAAGAATTCTTGTTTAAAAAAAAAAAAAAAAAGATCGGAAGAG
 35. : TGTACTCACATATAAT---AAATAAATAAATAAATAAATAAATAAATCTTT---AAAAAAAATTTCATAGTCTGGTCTGATCTTGATAGACCCAATTGAGAAGAATTCTTGTTTAAAAAAAAAAAAAAAAGAACGGAAGAGC
 36. : TGTACTCACATATAAT---AAATAAATAAATAAATAAATAAATAAATCTTT---AAAAAAAATTTCATAGTCTGGTCTGATCTTGATAGACCCAATTGAGAAGAATTCTTGTTTTAAAAAAAAAAAAAAAAAAAATCGGAAG
 37. : TGTACTCACATATAAT---AAATAAATAAATAAATAAATAAATAAATCTTT---AAAAAAAATTTCATAGTCTGGTCTGATCTTGATAGACCCAATTGAGAAGAATTCTTGTTTTAAAAAAAAAAAAAAAAAAAATCGGAAG
 38. : GGTACTCACATATAAT---AAATAAATAAATAAATAAATAAATAAATCTTT---AAAAAAAATTTCATAGTCTGGTCTGATCTTGATAGACCCACTTGAGAAGAATTCTTGTTTAAAAAAAAAAAAAAAAAATCTGAAAAGC
 39. : TGTACTCACATATAAT---AAATAAATAAATAAATAAATAAATAAATCTTT---AAAAAAAATTTCATAGTCTGGTCTGATCTTGATAGACCCAATTGAGAAGAATTCTTAAAAAAAAAAAAAAAAAAAAAATCGGAAAAAC
 40. : TGTACTCACATATAAT---AAATAAATAAATAAATAAATAAATAAATCTTT---AAAAAAAATTTCATAGTCTGGTCTGATCTTGATAGACCCAATTGAGAAGAATTCTTGTTTTAAAAAAAAAAAAAAAAAAAAAAAAAA
 41. : TGTACTCACATATAAT---AAATAAATAAATAAATAAATAAATAAATCTTT---AAAAAAAATTTCATAGTCTGGTCTGATCTTGATAGACCCAATTGAGAAGAATTCTTGTTTTAAAAAAAAAAAAAAAAAAAAAAAGGA
 42. : TGTACTCACATATAAT---AAATAAATAAATAAATAAATAAATAAATCTTT---AAAAAAAATTTCATAGTCTGGTCTGATCTTGATAGACCCAATTGAGAAGAATTCTTGTTTTAAAAAAAAAAAAAAAAAAAAAAATAGA
 43. : TGTACTCACATATAAT---AAATAAATAAATAAATAAATAAATAAATCTTT---TAAAAAAATTTCATAGTCTGGTCTGATCTTGATAGACCCAATTGAGAAGAATTCTTGTTTTAAAAAAAAAAAAAAAAAAAAACGGAAA
 44. : TGTACTCACATATAAT---AAATAAATAAATAAATAAATAAATAAATCTTT---AAAAAAAATTTCATAGTCTGGTCTGATCTTGATAGACCCAATTGAGAAGAATTCTTGTTTTAAAAAAAAAAAAAAAAAGATCGGAAGA
 45. : TGTACTCACATATAAT---AAATAAATAAATAAATAAATAAATAAATCTTT---AAAAAAAATTTCATAGTCTGGTCTGATCTTGATAGACCCAATTGAGAAGAATTCTTGTTTAAAAAAAAAAAAAAAAAAAAAAAAATC
 46. : TGTACTCACATATAAT---AAATAAATAAATAAATAAATAAATAAATCTTT--AAAAAAAAATTTCATAGTCTGGTCTGATCTTGATAGACCCAATTGAGAAGAATTCTTGTTTAAAAAAAAAAAAAAAAAAAATCGGAAGA
 47. : TGTACTCACATATAAT---AAATAAATAAATAAATAAATAAATAAATATTT--AAAAAAAAATTTCATAGTCTGGTCTGATCTTGATAGACCCAATTGAGAAGAATTCTTGTTTAAAAAAAAAAAAAAAAAAAAACGAAAAA
 48. : TGTACTCACATATAAT---AAATAAATAAATAAATAAATAAATAAATCTTT--AAAAAAAAATTTCATAGTCTGGTCTGATCTTGATAGACCCAATTGAGAAGAATTCTTGTTTTAAAAAAAAAAAAAAAAAAAACCGAAAAA
 49. : TGTACTCACATATAAT---AAATAAATAAATAAATAAATAAATAAATCTTT--AAAAAAAAATTTCATAGTCTGGTCTGATCTTGATAGACCCAATTGAGAAGAATTCTTGTTTAAAAAAAAAAAAAAAAAAAAAAGATCGG
 50. : TGTACTCACATATAAT---AAATAAATAAATAAATAAAAAAATAAATCTTT---AAAAAAAATTTCATAGTCTGGTCTGATCTTGATAGACCCAATTGAGAAGAATTCTTGTTTTAAAAAAAAAAAAAAAAAAAACGAAAAA
 51. : TGTACTCACATATAAT---AAATAAATAAATAAATAAATAAATAAATCTTT---AAAAAAAATTTCATCGTCTGGTCTGATCTTGATAGACCCAATTGAGAAGAATTCTTGTTTTAAAAAAAAAAAAAAAAAAAAAGACCGT
 52. : TGTACTCACATATAAT---AAATAAATAAATAAATAAATAAATAAATCTTT---AAAAAAAATTTCATAGTCTGGTCTGATCTTGATAGACCCAATTGAGAAGAATTCTTAAAAAAAAAAAAAAAAAAAAAAAACGGAAAACC
 53. : TGTACTCACATATAAT---AAATAAATAAATAAATAAATAAATAAATCTTT-AAAAAAAAAATTTCATAGTCTGGTCTGATCTTGATAGACCCAATTGAGAAGAATTCTTGTTAAAAAAAAAAAAAAAAAAAAAAAAACCAA
 54. : TTTACTCACATATAAT---AAATAAATAAATAAATAAATAAATAAATCTTT--AAAAAAAAATTTCATAGTCTGGTCTGATCTTGATAGACCCAATTGAGAAGAATTCTTGTTTAAAAAAAAAAAAAAAAAAAAAAAAAAA
 55. : TGTACTCACATATAAT--AAAATAAATAAATAAATAAATAAAAAAAATCTTT--AAAAAAAAATTTCATAGTCTGGTCTGATCTTGATAGACCCAATTGAGAAGAATTCTTGTTTAAAAAAAAAAAAAAAAAAAAAATCAAAA
 56. : TGTACTCACATATAAT--AAAATAAATAAATAAATAAATAAATAAATCTTT--AAAAAAAAATTTCATAGTCTGGTCTGATCTTGATAGACCCAATTGAGAAGAATTCTTGTTTAAAAAAAAAAAAAAAAAAAAAATAGGAA
 57. : TGTACTCACATATAAT---AAATAAATAAATAAATAAATAAATAAATCTTT--AAAAAAAAATTTCATAGTCTGGTCTGATCTTGATAGACCCAATTGAGAAGAATTCTTGTAAAAAAAAAAAAAAAAAAAAACGGAAAATA
 58. : TGTACTCACATATAAT---AAATAAATAAATAAATAAATAAATAAATCTTT--AAAAAAAAATTTCATAGTCTGGTCTGATCTTGATAGACCCAATTGAGAAGAATTCTTGTTTAAAAAAAAAAAAAAAAAAACCATAAAAC
 59. : TGTACTCACATATAAT---AAATAAATAAATAAATAAATAAATAAATCTTT--AAAAAAAAATTTCATAGTCTGGTCTGATCTTGATAGACCCAATTGAGAAGAATTCTTGTAAAAAAAAAAAAAAAAAAAAATCGGTAATA
 60. : TGTACTCACATATAAT---AAATAAATAAATAAATAAATAAATAAATCTTA--AAAAAAAAATTTCATAGTCTGGTCTGATCTTGATAGACCCAATTGAGAAGAATTCTTAAAAAAAAAAAAAAAAAAAATCGGAATAACAC
 61. : TGTACTCACATATAAT---AAATAAATAAATAAATAAATAAATAAATCTTT---AAAAAAAATTTCATAGTCTGGTCTTATCTTGATAGACCCAATTGAGAAGAATTCTTGAAAAAAAAAAAAAAAAAAAACGGAATACAAA
 62. : TGTACTCACATATAAT---AAATAAATAAATAAATAAATAAATAAATCTTTAAAAAAAAAATTTCATAGTCTGGTCTGATCTTGATAGACCCAATTGAGAAGAATTCTAAAAAAAAAAAAAAAAAAATCGAAAAAGCACAC
 63. : TGTACTCACATATAAT---AAATAAATAAATAAATAAATAAATAAATCTTTAAAAAAAAAATTTCATAGTCTGGTCTGATCTTGATAGACCCAATTGAGAAGAATTCTAAAAAAAAAAAAAAAAAAATAGGAAAAACACAT
 64. : TGTACTCACATATAAT---AAATAAATAAATAAATAAATAAATAAATCTTT-AAAAAAAAAATTTCATAGTCTGGTCTGATCTTGATAGACCCAATTGAGAAGAATTCAAAAAAAAAAAAAAAAAAAAACGGAAGAGAAAAC
 65. : TGTACTCACATATAAT---AAATAAATAAATAAATAAATAAATAAATCTTT-AAAAAAAAAATTTCATAGTCTGGTCTGATCTTGATAGACCCAATTGAGAAGAATTCTTAAAAAAAAAAAAAAAAAAAAAAAAAAAAAACA
 66. : TGTACTCACATATAAT---AAATAAATAAATAAATAAATAAATAAATCTTT-AAAAAAAAAATTTCATAGTCTGGTCTGATCTTGATAGACCCAATTGAGAAGAATTCTTAAAAAAAAAAAAAAAAAAAAAAAAAAAAAACTT
 67. : TGTACTCACATATAAT---AAATAAATAAATAAATAAATAAATAAATCTTT----AAAAAAATTTCATAGTCTGGTCTGATCTTGATAGACCCAATTGAGAAGAATTCTAAAAAAAAAAAAAAAAAAAAAAAAACCGAAAAA
 68. : TGTACTCACATATAAT---AAATAAATAAATAAATAAATAAATAAATCTTT----AAAAAAATTTCATAGTCTGGTCTGATCTTGATAGACCCAATTGAGAAGAATTCTAAAAAAAAAAAAAAAAAAAAAAAAAATCGAAAAA
 69. : TGTACTCACATATAAT---AAATAAATAAATAAATAAATAAATAAATCTTT---AAAAAACTTTCATAGTCTGGTCT-ATCTTGATAGACCCAATTGAGAAGAATTCTTAAAAAAAAAAAAAAAAAAAAAAGATCGGAAA
 70. : TGTACTCACATATAAT---AAATAAATAAATAAATAAATAAATAAATCTTT---AAAAAAAATTTCATAGTCTGGTCT-ATCTTGATAGACCCAATTGAGAAGAATTCTTAAAAAAAAAAAAAAAAAAAAAAAAAAGAAAA
 71. : TGTACTCACATATAAT---AAATAAATAAATAAATAAATAAATAAATCTTT-AAAAAAAAAATTTCATAGTCT-CTCTGATCTTGATAGACCCAATTGAGAAGAATTCTTGTTTAAAAAAAAAAAAAAAAAAAAAAAAAACC
chr 6: TGTACTCACATATAAT---AAATAAATAAATAAATAAATAAATAAATCTTT---AAAAAAAATTTCATAGTCTGGTCTGATCTTGATAGACCCAATTGAGAAGAATTCTTGTTTTCTTTGAGACAGAATTCCACTATGTAG
```

```
        3'-end region of B2          TCTTT              TSD                                    TTCTTGTTTT   poly(A)
                                      Weak                                                     Downstream
                                    terminator                                                 terminator
                                              The distance between two terminators is 50 bp.
```

D. B2 copy coordinates: chr7:99464046_99464222

```
                       *        20         *        40         *        60         *        80         *       100         *       120         *       140
  1. : TGTACTTACATATAATAAATAAATAAGTCTT---AAAAAAAAAAAAAAAGTCAGCACAAGCTGCGTCCACTGACAACCTCAGCTGGACTTGAGAATGCACGCTCTGCGCTAGATTTTTAAAAAAAAAAAAAAAAAAAAAAAA
  2. : TGTACTTACATATAATAAATAAATAAGTCTTAAAAAAAAAAAAAAAAAAAACAGCACAAGCTGCGTCCACTGACAACCTCAGCTGGACTTGAGAATGCACGCTCTGCGCTAGATTTTTAAAAAAAAAAAAAAAAAAAAAAAA
  3. : TGTACTTACATATAATAAATAAATAAGTCTTAAAAAAAAAAAAAAAAAAGTCAGCACAAGCTGCGTCCACTGACAACCTCAGCTGGACTTGAGAATGCACGCTCTGCGCTAGATTTTTAAAAAAAAAAAAAAAAAAAATCGG
  4. : TGTACTTACATATAATAAATAAATAAGTCTT-AAAAAAAAAAAAAAAAAGTCAGCACAAGCTGCGTCCACTGACAACCTCAGCTGGACTTGAGAATGCACGCTCTGCGCTAGATTTTAAAAAAAAAAAAAAAAAAAAATCGG
  5. : TGTACTTACATATAATAAATAAATAAGTCTT---AAAAAAAAAAAAAAAGTCAGCACAAGCTGCGTCCACTGACAACCTCAGCTGGACTTGAGAATGCACGCTCTGCGCTAGATTTTAAAAAAAAAAAAAAAAGATCGGAAG
  6. : TGTACTTACATATAATAAATAAATAAGTCTT---AAAAAAAAAAAAAAAGTCAGCACAAGCTGCGTCCCCTGACAACCTCAGCTGGACTTGAGAATGCACGCTCTGCGCTAGATTTTTAAAAAAAAAAAAAAAAAAAAAATA
  7. : TGTACTTACATATAATAAATAAATAAGTCTT----AAAAAAAAAAAAAAGTCAGCACAAGCTGCGTCCACTGACAACCTCAGCTGGACTTGAGAATGCACGCTCTGCGCTAGATTTTTAAAAAAAAATAAAAAAAAAAAAAA
  8. : TGTACTTACATATAATAAATAAATAAGTCTT--AAAAAAAAAAAAAAAAGTCAGCACAAGCTGCGTCCACTGACAACCTCAGCTGGACTTGAGAATGCCCGCTCTGCGCTAGATTTTAAAAAAAAAAAAAAAAAACCAAAAA
  9. : TGTACTTACATATAATAAATCAATAAGTCTT--AAAAAAAAAAAAAAAAGTCAGCACAAGCTGCGCCCACTGACAACCTCAGCTGGACTTTAGAATCCACGCTCTGCGCTAGATTTTAAAAAAAAAAAAAAAAATAGGAAAA
 10. : TGTACTTACATATAATAAATACATAAGTCTT-AAAAAAAAAAAAAAAAAGTCANCACAAGCTGCGTCCACTGACAACCTCAGCTGGACTTGA-AATGCCCGCTCTGCGCTAGATTTTAAAAAAAAAAAAAAAAAAAAAAAAA
 11. : TGTACTTACATATAATAAATAAATAAGTCTT----AAAAAAAAAAAAAAGTAAGCACAAGCTGCGACCACTGACAACCTCAGCTGGACTTGAGAATGCACGCTCTGCGCTAGATTTTAAAAAAAAAAAAAAAAAAAAATACG
chr 7: TGTACTTACATATAATAAATAAATAAGTCTT-----AAAAAAAAAAAAAGTCAGCACAAGCTGCGTCCACTGACAACCTCAGCTGGACTTGAGAATGCACGCTCTGCGCTAGATTTTTACCACCAGAACAAAAAACCAGAGT
         3'-end region of B2   TCTT                                                                                    TTTTT        poly(A)
                            B2 rudimentary                                                                          Downstream
                             terminator                                                                             terminator
                                          The distance between rudimentary and downstream terminators is 75 nt
```

E. B2 copy coordinates: chr12:69798001-69798392

```
                       *        20         *        40         *        60         *        80         *       100         *
  1. : TGTACTCATATGCAT--AAAATAAATAAATCTTAAAAAT-AAAAGAACC-AAGTGTCTTTGACTTTACATGTGGGGCCAGCCAACAATTTTAAAAAAAAAAAAAAAAAAAAAAAA
  2. : TGTACTCATATGCAT--AAAATAAATAAATCTTAAAAAT-AAAAGAACC-AAGTGTCTTTGACTTTACATGTGGGGCCAGCCAACAATTTTTAAAAAAAAAAAAAAAAAAAAAAT
  3. : TGTACTCATATGCAT--AAAATAAATAAATCTTAAAAAT-AAAAGAACC-AAGTGTCTTTGACTTTACATGTGGGGCCAGCCAACAATTTTAAAAAAAAAAAAAAAAAAAAAAAT
  4. : TGTACTCATATGCAT--AAAATAAATAAATCTTAAAAAT-AAAAGAACC-AAGTGTCTTTGACTTTACATGTGGGGCCAGCCAACAATTTTTAAAAAAAAAAAAAAAAGATCGGA
  5. : TGTACTCATATGCAT--AAAATAAATAAATCTTAAAAAT-AAAAGAACC-AAGTGTCTTTGACTTTACATGTGGGGCCAGCCAACAATTTTAAAAAAAAAAAAAAAAAGATCGGA
  6. : TGTACTCATATGCAT--AAAATAAATAAATCTTAAAAAT-AAAAGAACC-AAGTGTCTTTGACTTTACATGTGGGGCCAGCCAACAATTTTAAAAAAAAAAAAAAAAAGATCGGA
  7. : TGTACTCATATGCAT--AAAATAAATAAATCTTAAAAAT-AAAAGAACC-AAGTGTCTTTGACTTTACATGTGGGGCCAGCCAACAATTTTTAAAAAAAAAAAAAAAGATCGGAA
  8. : TGTACTCATATGCAT--AAAATAAATAAATCTTAAAAAT-AAAAGAACC-AAGTGTCTTTGACTTTACATGTGGGGCCAGCCAACAATTTTTAAAAAAAAAAAAAAAGATCGGAA
  9. : TGTACTCATATGCAT--AAAATAAATAAATCTTAAAAAT-AAAAGAACC-AAGTGTCTTTGACTTTACATGTGGGGCCAGCCAACAATTTAAAAAAAAAAAAAAAAAGATCGGAA
 10. : TGTACTCATATGCAT--AAAATAAATAAATCTTAAAAAT-AAAAGAACC-AAGTGTCTTTGACTTTACATGTGGGGCCAGCCAACAATTTAAAAAAAAAAAAAAAAAGATCGGAA
 11. : TGTACTCATATGCAT--AAAATAAATAAATCTTAAAAAT-AAAAGAACC-AAGTGTCTTTGACTTTACATGTGGGGCCAGCCAACAATTTTTAAAAAAAAAAAAGATCGGAAGAG
 12. : TGTACTCATATGCAT--AAAATAAATAAATCTTAAAAAT-AAAAGAACC-AAGTGTCTTTGACTTTACATGTGGGGCCAGCCAACAATTTTTAAAAAAAAAAAAGATCGGAAGAG
 13. : -GTACTCATATGCAT--AAAATAAATAAATCTTAAAAAT-AAAAGAACC-AAGTGTCTTTGACTTTACATGTGGGGCCAGCCAACAATTTTAAAAAAAAAAAAAAAAAAGATCGG
 14. : TGTACTCATATGCAT--AAAATAAATAAATCTTAAAAAT-AAAAGAACC-AAGTGTCTTTGACTTTACATGTGGGGCCAGCCAACAATAAAAAAAAAAAAAAAAAGATCGGAAGA
 15. : TGTACTCATATGCAT--AAAATACATAAATCTTAAAAAT-AAAAGAACC-AAGTGTCTTTGACTTTACATGTGGGGCCAGCCAACAATTTTTAACAAAAAAAAAAAAAGATCGGA
 16. : TGTACTCATATGCAT--AAAATAAATAAATCTTAAAAAT-AAAAGAACC-AAGTGTCTTTGACTTTACATGTGGGGCCAGCCAACAATAAAACAAAAAAAAAAAAGATCGGAAAA
 17. : TGTACTCATATGCAT--AAAATAAATAAATCTTAAAAAT-AAAAGAACC-AAGTGTCTTTGACTTTACATGTGGGGCCAGCCAACAATTTTTAAAAAAAAAAAAAGATCGGAAAG
 18. : TGTACTCATATGCAT--AAAATAAATAAATCTTAAAAAT-AAAAGAACC-AAGTGTCTTTGACTTTACATGTGGGGCCAGCCAACAATTTAAAAAAAAAAAAAAAAAAAATCGGAA
 19. : TGTACTCATATGCAT--AAAATAAATAAATCTTAAAAAT-AAAAGAACC-AAGTGTCTTTGACTTTACATGTGGGGCCAGCCAACAATTTTAAAAAAAAAAAAAAAAAAATCGGA
 20. : TGTACTCATATGCAT--AAAATAAATAAATCTTAAAAAT-AAAAGAACCAAAGTGTCTTTGACTTTACATGTGGGGCCAGCCAACAATTTTAAAAAAAAAAAAAAAAGATCGGAA
 21. : TGTACTCATATGCAT--AAAATAAATAAATCTTAAAAAT-AAAAGAACC-AAGTGTCTTTGACTTTACATGTGGGGCCAGCCAACAATTTTAAAAAAAAAAAAAAAATCGGAAAG
 22. : TGTACTCATATGCAT--AAAATAAATAAATCTTAAAAAT-AAAAGAACC-AAGTGTCTTTGACTTTACATGTGGGGCCAGCCAACAATTTTAAAAAAAAAAAAAACGATCGGAAT
 23. : TGTACTCATATGCAT--AAAATAAATAAATCTTAAAAAT-AAAAGAACC-AAGTGTCTTTGACTTTACATGTGGGGCCAGCCAACAATTTTAAAAAAAAAAAAAAAGATCGGAAG
 24. : TGTACTCATATGCAT--AAAATAAATAAATCTTAAAAAT-AAAAGAACC-AAGTGTCTTTGACTTTACATGTGGGGCCAGCCAACAATTTTAAAAAAAAAAAAAAAAGATCGGAA
 25. : TGTACTCATATGCAT--AAAATAAATAAATCTTAAAAAT-AAAAGAACC-AAGTGTCTTTGACTTTACATGTGGGGCCAGCCAACAATTTTAAAAAAAAAAAAAAAAAAAAAGGA
 26. : TGTACTCATATGCAT--AAAATAAATAAATCTTAAAAAT-AAAAGAACC-AAGTGTCTTTGACTTTACATGTGGGGCCAGCCAACAATTTTAAAAAAAAAAAAAAAAAAAAATTG
 27. : TGTACTCATATGCAT--AAAATAAATAAATCTTAAAAAT-AAAAGAACC-AAGTGTCTTTGACTTTACATGTGGGGCCAGCCAACAATTTTAAAAAAAAAAAAAAAAAAAGATCG
 28. : TGTACTCATATGCAT--AAAATAAATAAATCTTAAAAAT-AAAAGAACC-AAGTGTCTTTGACTTTACATGTGGGGCCAGCCAACAATTTTAAAAAAAAAAAAAAAAAAATTCGA
 29. : TGTACTCATATGCAT--AAAATAAATAAATCTTAAAAAT-AAAAGAACC-AAGTGTCTTTGACTTTACATGTGGGGCCAGCCAACAATTTAAAAAAAAAAAAAAAAAAAAAATCGG
```

```
30. : TGTACTCATATGCAT--AAAATAAATAAATCTTAAAAAT-AAAAGAACC-AAGTGTCTTTGACTTTACATGTGGGGCCAGCCAACAATTTAAAAAAAAAAAAAAAAAGATCGAAA
31. : TGTACTCATATGCAT--AAAATAAATAAATCTTAAAAAT-AAAAGAACC-AAGTGTCTTTGACTTTACATGTGGGGCCAGCCAACAATAAAAAAAAAAAAAAAAAAATCGAAAAA
32. : TGTACTCATATGCAT--AAAATAAATAAATCTTAAAAAT-AAAAGAACC-AAGTGTCTTTGACTTTACATGTGGGGCCAGCCAACAATTTAAAAAAAAAAAAAAAAAAATCGGAA
33. : TGTACTCATATGCAT--AAAATAAATAAATCTTAAAAAT-AAAAGAACC-AAGTGTCTTTGACTTTACCTGTGGGGCCAGCCAACAATTTTTAAAAAAAAAAAAAAAAAAATCGG
34. : TGTACTCATATGCAT--AAAATAAATAAATCTTAAAAAT-AAAAGAACC-AAGTGTCTTTGACTTTACATGTGGGGCCAGCCAACAATTTTAAAAAAAAAAAAAAAAAAGATTGG
35. : TGTACTCATATGCAT--AAAATAAATAAATCTTAAAAAT-AAAAGAACC-AAGTGTCTTTGACTTTACATGTGGGGCCAGCCAACAATTTTAAAAAAAAAAAAAAAAAAAACTGG
36. : TGTACTCATATGCAT--AAAATAAATAAATCTTAAAAAT-AAAAGAACC-AAGTGTCTTTGACTTTACATGTGGGGCCAGCCAACAATTTTAAAAAAAAAAAAAAAAAAAAAAAA
37. : TGTACTCATATGCAT--AAAATAAATAAATCTTAAAAAT-AAAAGAACC-AAGTGTCTTTGACTTTACATGTGGGGCCAGCCAACAATTTTAAAAAAAAAAAAAAAAAAAAAAAA
38. : TGTACTCATATGCAT--AAAATAAATAAATCTTAAAAAT-AAAAGAACC-AAGTGTCTTTGACTTTACATGTGGGGCCAGCCAACAATTTTAAAAAAAAAAAAAAAAAAAAAAAA
39. : TGTACTCATATGCAT--AAAATAAATAAATCTTAAAAAT-AAAAGAACC-AAGTGTCTTTGACTTTACATGTGGGGCCAGCCAACAATTTTTAAAAAAAAAAAAAAAAAAAAAAA
40. : TGTACTCATATGCAT--AAAATAAATAAATCTTAAAAAT-AAAAGAACC-AAGTGTCTTTGACTTTACATGTGGGGCCAGCCAACAATTTTTAAAAAAAAAAAAAAAAAAAAAAA
41. : TGTACTCATATGCAT--AAAATAAATAAATCTTAAAAAT-AAAAGAACC-AAGTGTCTTTGACTTTACATGTGGGGCCAGCCAACAATTTTAAAAAAAAAAAAAAAAAAAAAAAA
42. : TGTACTCATATGCAT--AAAATAAATAAATCTTAAAAAT-AAAAGAACC-AAGTGTCTTTGACTTTACATGTGGGGCCAGCCAACAATAAAAAAAAAAAAAAAAAAAAAAAAAAA
44. : TGTACTCATATGCAT--AAAATAAATAAATCTTAAAAAT-AAAAGAACC-AAGTGTCTTTGACTTTACATGTGGGGCCAGCCAACAATTTTAAAAAAAAAAAAAAAAAAAAAAAA
45. : TGTACTCATATGCAT--AAAATAAATAAATCTTAAAAAT-AAAAGAACC-AAGTGTCTTTGACTTTACATGTGGGGCCAGCCAACAATAAAAAAAAAAAAAAAAAAAAAAAAAAA
46. : TGTACTCATATGCAT--AAAATAAATAAATCTTAAAAATAAAAGAACC-AAGTGTCTTTGACTTTACATGTGAGGCCAGCCAACAATTTTTAAAAAAAAAAAAAAAAAAAAAAA
47. : TGTACTCATATGCAT--AAAATAAATAAATCTTAAAAAT-AAAAGAACC-AAGTGTCTTTGACTTTACATGTGGGGCCAGCCAACAATTTTTAAAAAAAAAAAAAAAAAAAAAAA
48. : TGTACTCATATGCAT--AAAATAAATAAATCTTAAAAAT-AAAAGAACC-AAGTGTCTTTGACTTTACATGTGGGGCCAGCCAACAATTAAAAAAAGAAAAAAAAAAAAAAAAAA
49. : TGTACTCATATGCAT--TAAATAAATAAATCTTAAAAAT-AAAAGAACC-AAGTGTCTTTGACTTTACATGTGGGGCCAGCCAACAATTTTAAAAAAAAACAAAAAAAAAAAAAC
50. : TGTACTCATATGCAT--AAAATAAATAAATCTTAAAAAT-AAAAGAACC-AAGTGTCTTTGACTTTACATGTGGGGCCAGCCAACAATTTTAAAAAAAAAAAAAAAAAAAAAAAA
51. : TGTACTCATATGCAT--AAAATAAATAAATCTTAAAAAT-AAAAGAACC-AAGTGTCTTTGACTTTACATGTGGGGCCAGCCAACAATTTTAAAAAAAAAAAAAAAAAAAAAAAA
52. : TGTACTCATATGCAT--AAAATAAATAAATCTTAAAAAT-AAAAGAACC-AAGTGTCTTTGACTTTACATGTGGGGCCAGCCAACAATTTTTAAAAAAAAAAAAAAAAAAAAAAA
53. : TGTACTCATATGCAT--AAAATAAATAAATCTTAAAAAT-AAAAGAACC-AAGTGTCTTTGACTTTACATGTGGGGCCAGCCAACAATTTTAAAAAAAAAAAAAAAAAAAAAAAA
54. : TGTACTCATATGCAT--AAAATAAATAAATCTTAAAAAT-AAAAGAACC-AAGTGTCTTTGACTTTACATGTGGGGCCAGCCAACAATTTTAAAAAAAAAAAAAAAAAAAAAAAA
55. : TGTACTCATATGCAT--AAAATAAATAAATCTTAAAAAT-AAAAGAACC-AAGTGTCTTTGACTTTACATGTGGGGCCAGCCACCAATTTTAAAAAAAAAAAAAAAAAAAAAAAA
56. : TGTACTCATATGCAT--AAAATAAATAAATCTTAAAAAT-AAAAGAACC-AAGTGTCTTTGACTTTACATGTGGGGCCAGCCAACAATTTTAAAAAAAAAAAAAAAAAAAAAAAA
57. : TGTACTCATATGCAT--AAAATAAATAAATCTTAAAAAT-AAAAGAACC-AAGTGTCTTTGACTTTACATGTGGGGCCAGCCAACAATTTTAAAAAAAAAAAAAAAAAAAAAAAA
58. : TGTACTCATATGCAT--AAAATAAATAAATCTTAAAAAT-AAAAGAACC-AAGTGTCTTTGACTTTACATGTGGGGCCAGCCAACAATTTTTATAAAAAAAAAAAAAAAAAAAAA
59. : TGTACTCATATGCAT--AAAATAAATAAATCTTAAAAAT-AAAAGAACC-AAGTGTCTTTGACTTTACATGTGGGGCCAGCCAACAATAAAAAAAAAAAAAAAAAATTTGGAAAA
60. : TGTACACATATGCAT--AAAATAAATAAATCTTAAAAAT-AAAAGAACC-AAGTGTCTTTGACTTTACATGTGGGGCCAGCCAACAATTAAAAAAAAAAAAAAAAGATCGGAAGA
61. : TGTACTCATATGCAT--AAAATAAATAAATCTTAAAAAT-AAAAGAACC-AAGTGTCTTTGACTTTACATGTGGGGCCAGCCAACAATAAAAAAAAAAAAAAAAAAATCGGAAAA
62. : TGTACTCATATGCAT--AAAATAAATAAATCTTAAAAAT-AAAAGAACC-AAGTGTCTTTGACTTTACATGTGGGGCCAGCCAACAATTAAAAAAAAAAAAAAAAAAAAAGATCG
63. : TGTACTCATATGCAT--AAAATAAATAAATCTTAAAAAT-AAAAGAACC-AAGTGTCTTTGACTTTACATGTGGGGCCAGCCAACAATTTTAAAAAAAAAAAAAAAGATCGGAAG
64. : TGTACTCATATGCAT--ACAATAAATAAATCTTAAAAAT-AAAAGAACC-AAGTGTCTTTGACTTTACATGTGGGGCCAGCCAACAATTTTAAAAAACAAAAAAAAAAAATCGGA
65. : TGTACTCATATGCAT--AAAATAAATAAATCTTAAAAAT-AAAAGAACC-AAGTGTCTTTGACTTTACATGTGGGGCCAGCCAACAATTTTAAAAAAAAAAAAAAAATATTGGAA
66. : TGTACTCATATGCAT--AAAATAAATAAATCTTAAAAAT-AAAAGAACC-AAGTGTCTTTGACTTTACATGTGGGGCCAGCCAACAATTAAAAAAAAAAAAAAAAAAAAAAAAAT
67. : TGTTCTCATATGCAT--AAAATAAATAAATCTTAAAAAT-AAAAGAACC-AAGTGTCTTTGACTTTACATGTGGGGCCAGCCAACAATTTTAAAAAAAAAAAAAAAAAAAAAATC
68. : TGTACTCATATGCATAAAAAATAAATAAATCTTAAAAAT-AAAAGAACC-AAGTGTCTTTGACTTTACATGTGGGGCCAGCCAACAATTTAAAAAAAAAAAAAAAAAAAAAAAAA
69. : TGTACTCATATGCAT--AAAATAAATAAATCTTAAAAAT-AAAAGAACCAAAGTGTCTTTGACTTTACATGTGGGGCCAGCCAACAATTTTAAAAAAAAAAAAAAAAATTCGGAA
70. : TGTACTCATCTGCAT--AAAATAAATAAATCTTAAAAAT-AACAGAACC-AAGTGTCTTTGACTTTACATGTGGGGCCAGCCAACAATTTTAAAAAAAAAAAAAAAAGATCGGAA
Chr12: TGTACTCATATGCAT--AAAATAAATAAATCTTAAAAAT-AAAAGAACC-AAGTGTCTTTGACTTTACATGTGGGGCCAGCCAACAATTTTTATTTATTTATTATTTTTTGAGAC
         3'-end region of B2        TCTT                                                    TTTTT    poly(A)
                               B2 rudimentary                                              Downstream
                                terminator                                                 terminator
```

The distance between rudimentary and downstream terminators is 52 bp.

F. B2 copy coordinates: chr13:97532360_97532533

```
                    *        20         *        40         *        60         *        80         *        100
 1. : TGTACTCATATAAATAAAATTAAAT--AAAAATGTT-AAAAAAC---AAAAAC----AAAAAACCTTGTGAACTAAATAAACCTTTTAAAAAAAAAAAAAAAAAAAAA
 2. : TGTACTCATATAAATAAAATTAAAT--AAAAATGTT-AAAAAAC---AAAAAC----AAAAAACCTTGTGAACTAAATAAACCTTTTAAAAAAAAAAAAAAAAAAAAA
 3. : TGTACTCATATAAATAAAATTAAAT--AAAAATGTT-AAAAAAC---AAAAAC----AAAAAACCTTGTGAACTAAATAAACCTTTTAAAAAAAAAAAAAAAAGATCG
 4. : TGTACTCATATAAATAAAATTAAAT--AAAAATGTT-AAAAAAC---AAAAAC----AAAAAACCTTGTGAACTAAATAAACCTTTTAAAAAAAAAAAAAAAAGATCG
 5. : TGTACTCATATAAATAAAATTAAAT--AAAAATGTT-AAAAAAC---AAAAAC----AAAAAACCTTGTGAACTAAATAAACCTTTTAAAAAAAAAAAAAAAAGATCG
 6. : TGTACTCATATAAATAAAATTAAAT--AAAAATGTT-AAAAAAC---AAAAAC----AAAAAACCTTGTGAACTAAATAAACCTTTAAAAAAAAAAAAAAAAAGATCG
```

```
 7. : TGTACTCATATAAATAAAATTAAAT--AAAAATGTT-AAAAAAC---AAAAAC----AAAAAACCTTGTGAACTAAATAAACCTTTAAAAAAAAAAAAAAAAAGATCG
 8. : TGTACTCATATAAATAAAATTAAAT--AAAAATGTT-AAAAAAC---AAAAAC----AAAAAACCTTGTGCACTAAATAAACCTTTTAAAAAAAAAAAAAAAAGATCG
 9. : TGTACTCATATAAATAAAATTAAAT--AAAAATGTT-AAAAAAC---AAAAAC----AAAAAACCTTGTGAACTAAATAAACCTTTTAAAAAAAAAAAAAAAAGATCG
10. : TGTACTCATATAAATAAAATTAAAT--AAAAATGTT-AAAAAAC---AAAAAC----AAAAAACCTTGTGAACTAAATAAACCTTTAAAAAAAAAAAAAAAAGATCGG
11. : TGTACTCATATAAATAAAATTAAAT--AAAAATGTTAAAAAAAC---AAAAAC----AAAAAACCTTGTGAACTAAATAAACCTTTTAAAAAAAAAAAAAAAGATCGG
12. : TGTACTCATATAAATAAAATTAAAT--AAAAATGTT-AAAAAAC---AAAAAC----AAAAAACCTTGTGAACTAAATAAACCTTTTAAAAAAAAAAAAAAAAAAAGA
13. : TGTACTCATATAAATAAAATTAAAT--AAAAATGTT-AAAAAAC---AAAAAC----AAAAAACCTTGTGAACTAAATAAACCTTTAAAAAAAAAAAAAAAAAGATCG
14. : TGTACTCATATAAATAAAATTAAAT--AAAAATGTT-AAAAAAC---AAAAAC----AAAAAACCTTGTGAACTAAATAAACCTTTTTAAAAAAAACAAAAAAAAGAT
15. : TGTACTCATATAAATAAAATTAAAT--AAAAATGTT-AAAAAAC---AAAAAC----AAAAAACCTTGTGAACTAAATAAACCTTTTAAAAAAAAAAAAAAAAAGATC
16. : TGTACTCATATAAATAAAATTAAAT--AAAAATGTT-AAAAAAC---AAAAAC----AAAAAACCTTGTGAACTAAATAAACCTTTAAAAAAAAAAAAAAAAAAAGAT
17. : TGTACTCATATAAATAAAATTAAAT--AAAAATGTT-AAAAAAC---AAAAAC----AAAAAACCTTGTGAACTAAATAAACCTTTAAAAAAAAAAAAAAAAAAAAAT
18. : TGTACTCATATAAATAAAATTAAAT--AAAAATGTT-AAAAAAC---AAAAAC----AAAAAACCTTGTGAACTAAATAAACCTTTAAAAAAAAAAAAAAAAAAAAAC
19. : TGTACTCATATAAATAAAATTAAAT--AAAAATGTT-AAAAAAC---AAAAAC----AAAAAACCTTGTGAACTAAATAAACCTTTTTAAAAAAAAAAAAAAAAAAAAA
20. : TGTACTCATATAAATTAAATTAAAT--AAAAATGTT-AAAAAAC---AAAAAC----AAAAAACCTTGTGAACTAAATAAACCTTTAAAAAAAAAAAAAAAAAGATCG
21. : TGTACTCATATAAATAAAATTAAAT--AAAAATGTT-AAAAAAC---AAAAAC----AAAAAACCTTGTGAACTAAATAAACCTTTAAAAAAAAAAAAAAAAAGATCG
22. : TGTACTCATATAAATAAAATTAAAT--AAAAATGTT-AAAAAAC---AAAAAC-AAAAAAAAACCTTGTGAACTAAATAAACCTTTAAAAAAAAAAAAAAAAAGATCG
23. : TGTACTCATATAAATAAAATTAAAT--AAAAATGTT-AAAAAAC---AAAAAC----AAAAAACCTTGTGAACTAAATAAACCTTTAAAAAAAAAAAAAAAAAGATCG
24. : TGTACTCATATAAATAAAATTAAAT--AAAAATGTT-AAAAAAC---AAAAAC----AAAAAACCTTGTGAACTAAATAAACCTTTTAAAAAAAAAAAAAAAAAAAAA
25. : TGTACTCATATAAATAAAATTAAAT--AAAAATGTT-AAAAAAC---AAAAAC----AAAAAACCTTGTGAACTAAATAAACCTTTAAAAAAAAAAAAAAAAAAAAAA
26. : TGTACTCATATAAATAAAATTAAAT--AAAAATGTT-AAAAAAC---AAAAAC----AAAAAACCTTGTGAACTAAATAAACCTTTAAAAAAAAAAAAAAAAAAAAAA
27. : TGTACTCATATAAATAAAATTAAAT--AAAAATGTT-AAAAAAC---AAAAAC----AAAAAACCTTGTGAACTAAATAAACCTTTAAAAAAAAAAAAAAAAAAAAAA
28. : TGTACTCATATAAATAAAATTAAAT--AAAAATGTT-AAAAAAC---AAAAAC----AAAAAACCTTGTGAACTAAATAAACCTTTAAAAAAAAAAAAAAAAAAAAAA
29. : TGTACTCATATAAATAAAATTAAAT--AAAAATGTT-AAAAAAC---AAAAAC----AAAAAACCTTGTGAACTAAATAAACCTTTAAAAAAAAAAAAAAAAAAAAAA
30. : TGTACTCATATAAATAAAATTAAAT--AAAAATGTT-AAAAAAC---AAAAACAAAAAAAAAACCTTGTGAACTAAATAAACCTTTTAAAAAAAAAAAAAAAAAAAAA
31. : TGTACTCATATAAATAAAATTAAAT--AAAAATGTT-AAAAAAC---AAAAAC----AAAAAACCTTGTGAACTAAATAAACCTTTTTTAAAAAAAAAAAAAAAAAAA
32. : TGTACTCATATAAATAAAATTAAAT--AAAAATGTT-AAAAAAC---AAAAAC----AAAAAACCTTGTGAACTAAATAAACCTTTAAAAAAAAAAAAAAAAAAAAAA
33. : TGTACTCATATAAATAAAATTAAAT--AAAAATGTT-AAAAAAC---AAAAAC----AAAAAACCTTGTGAACTAAATAAACCTTTAAAAAAAAAAAAAAAAAAAAAA
34. : TGTACTCATATAAATAAAATTAAAT--AAAAATGTT-AAAAAAC---AAAAAC----AAAAAACCTTGTGAACTAAATAAACCTTTAAAAAAAAAAAAAAAAAAAGAT
35. : TGTACTCATATAAATAAAATTAAAT--AAAAATGTT-AAAAAAC---AAAAAC----AAAAAACCTTGTGAACTAAATAAACCTTTTTAAAAAAAAAAAAAAAAAAAA
36. : TGTACTCATATAAATAAAATTAAAT--AAAAATGTT-AAAAAAC---AAAAAC----AAAAAACCTTGTGAACTAAATAAACCTTTAAAAAAAAAAAAAAAAAAAAAC
37. : TGTACTCATATAAATAAAATTAAAT--AAAAATGTT-AAAAAAC---AAAAAC----AAAAAACCTTGTGAACTAAATAAACCTTTTAAAAAAAAAAAAAAAAAAAAA
38. : TGTACTCATATAAATAAAATTAAAT--AAAAATGTT-AAAAAAC---AAAAAC----AAAAAACCTTGTGAACTAAATAAACCTTTTTTAAAAAAAAAAAAAAAAAAA
39. : TGTACTCATATAAATAAAATTAAAT--AAAAATGTT-AAAAAAC---AAAAAC----AAAAAACCTTGTGAACTAAATAAACCTTTTAAAAAAAAAAAAAAAAAAAAA
40. : TGTACTCATATAAATAAAATTAAAT--AAAAATGTT-AAAAAAC---AAAAAC----AAAAAACCTTGTGAACTAAATACACCTTTTTAAAAAAAAAAAAAAAAAGAT
41. : TGTACTCATATAAATAAAATTAAAT--AAAAATGTT-AAAAAAC---AAAAAC----AAAAAACCTTGTGAACTAAATAAACCTTTAAAAAAAAAAAAAAAGATCGGA
42. : TGTACTCATATAAATAAAATTAAAT--AAAAATGTT-AAAAAAC---AAAAAC----AAAAAACCTTGAGAACTAAATAAACCTTTTTAAAAAAAAAAAAAAAAAAAT
43. : TGTACTCATATAAATAAAATTAAAT--AAAAATGTT-AAAAAAC---AAAAAC----AAAAAACCTTGTGAACTAAATAAACCTTTTTAAAAAAAAAAAAAAAAAAAA
44. : TGTACTCATATATATAAAATTAAAT--AAAAATGTT-AAAAAAC---AAAAAC----AAAAAACCTTGTGAACTAAATAAACCTTTTAAAAAAAAAAAAAAAAAAATC
45. : TGTACTCATATAAATAAAATTAAAT--AAAAATGTT-AAAAAAC---AAAAAC----AAAAAACCTTGTGAACTAAATAAACCTTTTTAAAAAAAAAAAAAAAAAAAA
46. : TGTACTCATATAAATAAAATTAAAT--AAAAATGTT-AAAAAAC---AAAAAC----AAAAAACCTTGTGAACTAAATAAACCTTTTAAAAAAAAAAAAAAAAAAAAA
47. : TGTACTCATATAAATAAAATTAAAT--AAAAATGTT-AAAAAAC---AAGAAC----AAAAAACCTTGTGAACTAAATAAACCTTTTAAAAAAAAAAAAAAAAAAAAA
48. : TGTACTCATATAAATAAAATTAAAT--AAAAATGTT-AAAAAAC---AAAAAC----AAAAAACCTTGTGAACTAAATAAACCTTTTAAAAAAAAAAAAAAAAAAAAA
49. : TGTACTCATATAAATAAAATTAAAT--AAAAATGTT-AAAAAAC---AAAAAC----AAAAAACCTTGTGAACTAAATAAACCTTTAAAAAAAAAAAAAAAAAATCGG
50. : TGTACTCATATAAATAAAATTAAAT--AAAAATGTT-AAAAAAC---AAAAAC----AAAAAACCTTGTGAACTAAATAAACCTTTAAAAAACAAAAAAAAAGATCGG
52. : TGTACTCATATAAATAAAATTAAAT--AAAAATGTT-AAAAAAC---AAAAAC-----AAAAACCTTGTGAACTAAATAAACCTTTAAAAAAAAAAAAAAAAGATCGG
53. : TGTACTCATATAAATAAAATTAAAT--AAAAATGTT-AAAAAAC---AAAAAC----AAAAAACCTTGTGAACTAAATAAACCTTTTAAAAAAAAAAAAAAAAAAAGC
54. : TGTACTCATATAAATAAAATTAAAT--AAAAATGTT-AAAAAAC---AAAAAC-----AAAAACCTTGTGAACTAAATAAACCTTTAAAAAAAAAAAAAAAAAAAAAA
55. : TGTACTCATATAAATAAAATTAAAT--AAAAATGTT-AAAAAAC---AAAAACAAAAAAAAAACCTTGTGAACTAAATAAACCTTTAAAAAAAAAAAAAAAAAAAAAC
56. : TGTACTCATATAAATAAAATTAAAT--AAAAATGTT-AAAAAAC---AAAAACAAAAAAAAAACCTTGTGAACTAAATAAACCTTTAAAAAAAAAAAAAAAAAAAAAA
57. : TGTACTCATATAAATAAAATTAAAT--AAAAATGTT-AAAAAAC---AAAAAC----AAAAAACCTTGTGAACTAAATAAACCTTTTAAAAAAAAAAAAAAAAAAAAT
58. : TGTACTCATATAAATAAAATTAAAT--AAAAATGTT-AAAAAAC-AAAAAAAC----AAAAAACCTTGTGAACTAAATAAACCTTTTAAAAAAAAAAAAAAAAAAGAT
59. : TGTACTCATATAAATAAAATTAAAT--AAAAATGTTAAAAAAAC-AAAAAAAC----AAAAAACCTTGTGAACTAAATAAACCTTTTTTAAAAAAAAAAAAAAAAAAA
60. : TGTACTCATATAAATAAAATTAAAT--AAAAATGTT-AAAAAAC---AAAAAC----AAAAAACCTTGTGAACTAAATAAACCTTTAAAAAAAAAAAAAAAAAAAGAT
61. : TGTACTCATATAAATAAAATTAAAT--AAAAATGTT-AAAAAAC---AAAAAC----AAAAAACCTTGTGAACTAAATAAACCTTTAAAAAAAAAAAAAAAGATCGGA
62. : TGTACTCATATAAATAAAATTAAAT--AAAAATGTT-AAAAAAC---AAAAAC----AAAAAACCTTGTGAACTAAATAAACCTTTAAAAAAAAAAAAAAACATCGGA
63. : TGTACTCATATAAATAAAATTAAATAAAAAAATGTT-AAAAAAC---AAAAAC----AAAAAACCTTGTGAACTAAATAAACCTTTAACAAAAAAAAAAAAAAATCGG
64. : TGTACTCATATAAATAAAATTAAATAAAAAAATGTT-AAAAAAC---AAAAAC----AAAACACCTTGTGAACTAAATAAACCTTTAACAAAAAAAAAAAAAACCCGA
```

```
 65. : TGTACTCATATAAATAAAATTAAAT--AAAAATGTT-AAAAAAC---AAAAAC----AAAAAACCTTGTGTACTAAATAAACCTTTTAAAAAAAAAAAAAGATCGGAA
 66. : TGTACTCATATAAATAAAATTAAAT--AAAAATGTT-AAAAAACAAAAAAAAC----AAAAAACCTTGTGAACTAAATAAACCTTTAAAAAAAAAAAAAAAAAAAAAC
 67. : TGTACTCATATCAATAAAATTAAAT--AAAAATGTTAAAAAAAC---AAAAAC----AAAAAACCTTGTGAACTAAATAAACCTTTACAAAAAAAAAAAAAAAAAAAA
 68. : TGTACTCATATAAATAAAATTAAAT--AAAAATGTT-AAAAAAC---AAAAAC----ACAAACCCTTGTGCACTAAATAAACCTTTAAAAAAAAAAAAAAAAAAAAAA
Chr13: TGTACTCATATAAATAAAATTAAAT--AAAAATGTT-AAAAAAC---AAAAAC----AAAAAACCTTGTGAACTAAATAAACCTTTTTTCCATTTTTATTGGTTATTT
           3’-end region of B2         TGTT                                              TTTTTT   poly(A)
                                     B2 rudimentary                                    Downstream
                                     terminator                                        terminator

                                 The distance between rudimentary and downstream terminators is 39 bp.
```

G. B2 copy coordinates: chr17:80489756_80489931

```
               *        20         *        40         *        60         *        80         *       100         *
  1. : TGTACTCATATATGATAATAAATAAATCTT----AAAAAAAAAAGATAACTCCCTTTCTCTGACTGAAG-AAAACTTGTTTCCTTTAAAAAAAAAAAAAAAAAAAAGATCGG
  2. : TGTACTCATATATGATAATAAATAAATCTT-----AAAAAAAAAGATAACTCCCTTTCTCTGACTGAAG-AAAACTTGTTTCCTTTAAAAAAAAAAAAAAAAAAAAGATCGG
  3. : TGTACTCATATATGATAATAAATAAATCTT-----AAAAAAAAAGATAACTCCCTTTCTCTGACTGAAG-AAAACTTGTTTCCTTTTAAAAAAAAAAAAAAAAACAGATCGG
  4. : TGTACTCATATATGATAATAAATAAATCTT---AAAAAAAAAAAGATAACTCCCTTTCTCTGACTGAAG-AAAACTTGTTTCCTTTTAAAAAAAAAAAAAAAAAGATCGGAA
  5. : TGTACTCATATATGATAATAAATAAATCTT---AAAAAAAAAAAGATAACTCCCTTTCTCTGACTGAAG-AAAACTTGTTTCCTTTTAAAAAAAAAAAAAAAAAAAAAAAAA
  6. : TGTACTCATATATGATAATAAATAAATCTT---AAAAAAAAAAAGATAACTCCCTTTCTCTGACTGAAG-AAAACTTGTTTCCTTTTAAAAAAAAAAAAAAAAAAAAAAAAT
  7. : TGTACTCATATATGATAATAAATAAATCTT---AAAAAAAAAAAGATAACTCCCTTTCTCTGACTGAAG-AAAACTTGTTTCCTTTAAAAAAAAAAAAAAAAAAAAAAAAAA
  8. : TGTACTCATATATGATAATAAATAAATCTT---AAAAAAAAAAAGATAACTCCCTTTCTCTGACTGAAG-AAAACTTGTTTCCTTTTAAAAAAAAAAAAAAAAAAAAAAAAA
  9. : TGTACTCATATATGATAATAAATAAATCTT---AAAAAAAAAAAGATAACTCCCTTTCTCTGACTGAAG-AAAACTTGTTTCCTTTAAAAAAAAAAAAAAAAAAAAAAAAAA
 10. : TGTACTCATATATGATAATAAATAAATCTT---AAAAAAAAAAAGATAACTCCCTTTCTCTGACTGAAG-AAAACTTGTTTCCTTTAAAAAAAAAAAAAAAAAAAAAAAAAA
 11. : TGTACTCATATATGATAATAAATAAATCTT----AAAAAAAAAAGATAACTCCCTTTCTCTGACTGAAG-AAAACTTGTTTCCTTTTAAAAAAAAAAAAAAAAAAAAAAAAA
 12. : TGTACTCATATATGATAATAAATAAATCTT----AAAAAAAAAAGATAACTCCCTTTCTCTGACTGAAG-AAAACTTGTTTCCTTTTAAAAAAAAAAAAAAAAAAAAAAAAA
 13. : TGTACTCATATATGATAATAAATAAATCTT----AAAAAAAAAAGATAACTCCCTTTCTCTGACTGAAG-AAAACTTGTTTCCTTTTAAAAAAAAAAAAAAAAAAAAAAAAA
 14. : TGTACTCATATATGATAATAAATAAATCTT----AAAAAAAAAAGATAACTCCCTTTCTCTGACTGAAG-AAAACTTGTTTCCTTTTAAAAAAAAAAAAAAAAAAAAAAAAA
 15. : TGAACTCATATATGATAATAAATAAATCTT----AAAAAAAAAAGATAACTCCATTTCTCTGACTGAAG-AAAACTTGTTTCCTTTTAAAAAAAAAAAAAAAAAAAAAAAAA
 16. : TGTACTCATATATGATAATAAATAAATCTT---AAAAAAAAAAAGATAACTCCCTTTCTCTGACTGAAG-AAAACTTGTTTCCTTTTAAAAAAAAAAAAAAAAAAAAAAAAA
 17. : TGTACTCATATATGATAATAAATAAATCTT----AAAAAAAAAAGATAACTCCCTTTCTCTGACTGAAG-AAAACTTGTTTCCTTTTAAAAAAAAAAAAAAAAAAAAAAGAT
 18. : TGTACTCATATATGATAATAAATAAATCTT----AAAAAAAAAAGATAACTCCCTTTCTCTGACTGAAG-AAAACTTGTTTCCTTTTAAAAAAAAAAAAAAAAAAAAAAAAC
 19. : TGTACTCATATATGATAATAAATAAATCTT---AAAAAAAAAAAGATAACTCCCTTTCTCTGACTGAAG-AAAACTTGTTTCCTTTTAAAACAAAAAAAAAAAAAAAAAAAA
 20. : TGTACTCATATATGATAATAAATAAATCTT----AAAAAAAAAAGATAACTCCCTTTCTCTGACTGAAG-AAAACTTGTTTCCTTTTAAAAAAAAAAAAAAAAAAAAAAAAA
 21. : TGTACTCATATATGATAATAAATAAATCTT----AAAAAAAAAATATAACTCCCTTTCTCTGACTGAAG-AAAACTTGTTTCCTTTTAAAAAAAAAAAAAAAAAAAAAAAAA
 22. : TGTACTCGTATATGATAATAAATAAATCTT----AAAAAAAAAAGATAACTCCCTTTCTCTGACTGAAG-AAAACTTGTTTCCTTTAAAAAAAAAAAAAAAAAAAAAAAAAA
 23. : TGTACTCATATATGATAATAAATAAATCTT---AAAAAAAAAAAGATAACTCCCTTTCTCTGACTGAAG-AAAACTTGTTTCCTTTTAAAAAAAAAAAAAAAAAAATCGGAA
 24. : TGTACTCATATATGATAATAAATAAATCTT---AAAAAAAAAAAAATAACTCCCTTTCTCTGACTGAAG-AAAACTTGTTTCCTTTTAAAAAAAAAAAAAAAAAAAAATCGA
 25. : TGTACTCATATATGATAATAAATAAATCTT---AAAAAAAAAAAGATAACTCCCTTTCTCTGACTGAAG-AAAACTTGTTTCCTTTAAAAAAAAAAAAAAAAAAGATCGGAA
 26. : TGTACTCATATAAGATAATAAATAAATCTT----AAAAAAAAAAGATAACTCCCTTTCTCTGACTGAAG-AAAACTTGTTTCCTTTAAAAAAAAAAAAAAAAGATCGGAAGA
 27. : TGTACTCATATATGATAATAAATAAATCTT----AAAAAAAAAAGATAACTCCCTTTCTCTGACTGAAG-AAAACTTGTTTCCTTTTAAAAAAAAAAAAAAAAGATCGGAAG
 28. : TGTACTCATATATGATAATAAATAAATCTT----AAAAAAAAAAGATAACTCCCTTTCTCTGACTGAAG-AAAACTTGTTTCCTTTAAAAAAAAAAAAAAAAAAGATCGGAA
 29. : TGTACTCATATATGATAATAAATAAATCTT----AAAAAAAAAAGATAACTCCCTTTCTCTGACTGAAG-AAAACTTGTTTCCTTTAAAAAAAAAAAAAAAGATCGGAAGAG
 30. : TGTACTCATATATGATAATAAATAAATCTT----AAAAAAAAAAGATAACTCCCTTTCTCTGACTGAAG-AAAACTTGTTTCCTTTAAAAAAAAAAAAAAAGATCGGAAGAG
 31. : TGTACTCATATATGATAATAAATAAATCTT----AAAAAAAAAAGATAACTCCCTTTCTCTGACTGAAG-AAAACTTGTTTCCTTTAAAAAAAAAAAAAAAAAAATCGGAAG
 32. : TGTACTCATATATGATAATAAATAAATCTT----AAAAAAAAAAGATAACTCCCTTTCTCTGACTGAAG-AAAACTTGTTTCCTTAAAAAAAAAAAAAAAGATCGGAAGAGC
 33. : TGTACTCATATATGATAATAAATAAATCTT----AAAAAAAAAAGATAACTCCCTTTCTCTGACTGAAG-AAAACTTGTTTCCTTTAAAAAAAAAAAAAAAAAGATCGGAAA
 34. : TGTACTCATATATGATAATAAATAAATCTT----AAAAAAAAAAGATAACTCCCTTTCTCTGACTGAAA-AAAACTTGTTTCCTTAAAAAAAAAAAAAAAAAGACCGAAGAG
 36. : TGTACTCATATATGATAATAAATAAATCTT-----AAAAAAAAAGATAACTCCCTTTCTCTGACTGAAG-AAAACTTGTTTCCTTTTAAAAAAAAAAAAAAAAAGATCGGAA
 37. : TGTACTCATATATGATAATAAATAAATCTT-----AAAAAAAAAGATAACTCCCTTTCTCTGACTGAAG-AAAACTTGTTTCCTTTTAAAAAAAAAAAAAAAAAGATCGGAA
 38. : TGTACTCATATATGATAATAAATAAATCTT-----AAAAAAAAAGATAACTCCCTTTCTCTGACTGAAG-AAAACTTGTTTCCTTTAAAAAAAAAAAAAAAAAAAGATCGGA
 39. : TGTACTCATATATGATAATAAATAAATCTT----AAAAAAAAAAGATAACTCCCTTTCTCTGACTGAAG-AAAACTTGTTTCCTTTTAAAAAAAAAAAAAAAGATCGGAAGA
 40. : TGTACTCATATATGATAATAAATAAATCTT----AAAAAAAAAAGATAACTCCCTTTCTCTGACTGAAG-AAAACTTGTTTCCTTTTAAAAAAAAAAAAAAACGATCGGACG
 41. : TGTACTCATATATGATAATAAATAAATCTT----AAAAAAAAAAGATAACTCCCTTTCTCTGACTGAAG-AAAACTTGTTTCCTTTAAAAAAAAAAAAAAAAAAAAAAATCG
 42. : TGTACTCATATATGATAATAAATAAATCTT-----AAAAAAAAAGATAACTCCCTTTCTCTGACTGAAG-AAAACTTGTTTCCTTTTAAAAAAAAAAAAAAAAAAAAGATCG
 43. : TGTACTCATATATGATAATAAATAAATCTT----AAAAAAAAAAGATAACTCCCTTTCTCTGACTGAAG-AAAACTTGTTTCCTTTAAAAAAAAAAAAAAAAAAAAACCGAA
 44. : TGTACTCATATATGATAATAAATAAATCTT----AAAAAAAAAAGATAACTCCCTTTCTCTGACTGAAG-AAAACTTGTTTCCTTAAAAAAAAAAAAAAAAGATCGGAAGAG
```

```
 45. : TGTACTCATATATGATAATAAATAAATCTT-AAAAAAAAAAAAAGATAACTCCCTTTCTCTGACTGAAG-AAAACTTGTTTCCTTTAAAAAAAAAAAAAAAAGATCGGAAAA
 46. : TGTACTCATATATGATAATAAATAAATCTT-AAAAAAAAAAAAAGATAACTCCCTTTCTCTGACTGAAG-AAAACTTGTTTCCTTTAAAAAAAAAAAAAAAAAATCGGAAAA
 47. : TGTACTCATATATGATAATAAATAAATCTT-AAAAAAAAAAAAAGATAACTCCCTTTCTCTGCCTGAAG-AAAACTTGTTTCCTTTAAAAAAAAAAAAAAAAAATCGGAAGG
 48. : TGTACTCATATATGATAATAAATAAATCTT-AAAAAAAAAAAAAGATAAATCCCTTTCTCTGACTGAAG-AAAACTTGTTTCCTTTTAAAAAAAAAAAAAAAAAGAACGGAA
 49. : TGTACTCATATATGATAATAAATAAATCTT---AAAAAAAAAAAGATAACTCCCTTTCTCTGACTGAAG-AAAACTTGTTTCCTTTAAAAAAAAAAAAAAAAAGATCGGAAG
 50. : TGTACTCATATATGATAATAAATAAATCTT---AAAAAAAAAAAGATAACTCCCTTTCTCTGACTGAAG-AAAACTTGTTTCCTTTAAAAAAAAAAAAAAAAAAATCGGTAG
 51. : TGTACTCATATATGATAATAAATAAATCTT----AAAAAAAAAAGATAACTCCCTTTCTCTGACTGAAG-AAAACTTGTTTCCTTTTAAAAAAAAAAAAAAAAAAAAACCGA
 52. : TGTACTCATATATGATAATAAATAAATCTT----AAAAAAAAAAGATAACTCCCTTTCTCTGACTGAAG-AAAACTTGTTTCCTTTTAAAAAAAAAAAAAAAAAAGATCGAA
 53. : TGTACTCATATATGATAATAAATAAATCTT----AAAAAAAAAAGATAACTCCCTTTCTCTGACTGAAG-ACAACTTGTTTCCTTTTAAAAAAAAAAAAAAAAAAGATAGGA
 54. : TGTACTCATATATGATAATAAATAAATCTT----AAAAAAAAAAGATAACTCCCTTTCTCTGACTGAAG-AAAACTTGTTTCCTTTTAAAAAAAAAAAAAAAAAAAACCGAA
 55. : TGTACTCATATATGATAATAAATAAATCTT--AAAAAAAAAAAAGATAACTCCCTTTCTCTGACTGAAG-AAAACTTGTTTCCTTTAAAAAAAAAAAAAAAAGATAGAAAGA
 56. : TGTACTCATATATGATAATAAATAAATCTTAAAAAAAAAAAAAAGATAACTCCCTTTCTCTGACTGAAG-AAAACTTGTTTCCTTTTAAAAAAAAAAAAAAAAAAAAAAAAA
 57. : TGTACTCATATATGATAATAAATAAATCTT----AAAAAAAAAAGATAACTCCCTTTCTCTGACTGAAG-AAAACTTGTTTCCTTTTAAAAAAAAAAAAAAAAAAAAAAAAA
 58. : TGTACTCATATATGATAATAAATAAATCTTAAAAAAAAAAAAAAGATAACTCCCTTTCTCTGACTGAAG-AAAACTTGTTTCCTTTTAAAAAAAAAAAAAAAAAAAAATCGG
 59. : TGTACTCATATATGATAATAAATAAATCTT-AAAAAAAAAAAAAGATAACTCCCTTTCTCTGACTGAAG-AAAACTTGTTTCCTTTTAAAAAAAAAAAAAAAAAAAAATCAG
 60. : TGTACTCATATATGATAATAAATAAATCTT---AAAAAAAAAAAGATAACTCCCTTTCTCTGACTGAAG-AAACCTTGTTTCCTAAAAAACAAAAAAAAAAAAAAAAAAAAA
 61. : TGTACTCATATATGATAATAAATAAATCTT--AAAAAAAAAAAAGATAACTCCCTTTCTCTGACTGAAG-AAAACTTGTTTCCTTTTAAAAAACAAAAAAAAAAATCCGAAA
 62. : TGTACTCATATATGATAATAAATAAATCTT---AAAAAAAAAAAGATAACTCCCTTTCTCTGACTGAAG-AAAACTTGTTTCCTTTAAAAAAAAAAAAAAAAATCGGAAAAG
 63. : TGTACTCATATATTATAATAAATAAATCTT---AAAAAAAAAAAGATAACTCCCTTTCTCTGACTGAAG-AAAACTTGTTTCCTTTAAAAAAAAAAAAAAAAAAAAATCGAA
 64. : TGTACTCATATATGATAATAAATAAATCTT--AAAAAAAAAAAAGATAACTCCCTTTCTCTGACTGAAGAAAAACTTGTTTCCTTTTAAAAAAAAAAAAAAAAAAAATCGAA
 65. : TGTACTCATATATGATAATAAATAAATCTT----AAAAAAAAAAGATAACTCCCTTTCTCTGACTGAAG-AAAACTTGTTTCCTTTTAAAAAAAAAACAAAAAAAGATCGGA
 66. : TGTACTCATATATGATAATAAATAAATCTT----AAAAAAAAAAGATAACTCCCTTTCTCTGACTGAAG-AAAACTTGTTTAAAAAAAAAAAAAAAAAGATCGGAAGAGCAC
 67. : TGTACTCATATATGATAATAAATAAATCTT----AAAAAAAAAAAAAAACTCCCTTTCTCTGACCGAAG-AAAACTTGTTTCCTTTAAAAAAAAAACAAAAAAAAAACATCA
 68. : TGTACTCATATATGATAATAAATAAATCTT----AAAAAAAAAAAAAAACTCCCTTTCTCTGACTGAAG-AAAACTTGTTTCCTTTAAAAAAAAAAAAAAAAAACCAAAAAC
 69. : TGTACTCATATATGATAATAAATAAATCTT----AAAAAAAAAAGATAACTCCCTTTCTCTGACTGAAG-AAAACTTGTTTCCTTTTAAAAAAAAAAAAAAAGTAGGTAAGG
 70. : TGTACTCATATATGATAATAAATAAATCTT-----AAAAAAAAAGATAACTCCCTTTCTCTCACTGAAG-AAAACTTGTTTCCTTTAAAAAAAAAAAAAAAAAAAAAAAAAA
 71. : TGTACTCATATATGATAATAAATAAATCTT-----AAAAAAAAAGATAACTCCCTTTCTCTGACTGAAG-AAAACTTGTTTCCTTTAAAAAAAAAAAAAAAAAAATATCAGC
chr17: TGTACTCATATATGATAATAAATAAATCTT----AAAAAAAAAAGATAACTCCCTTTCTCTGACTGAAG-AAAACTTGTTTCCTTTTTCTTCTTTCTTTGTTTCCTTTCTTT
         3'-end region of B2      TCTT                TSD                                  TTTTT      poly(A)
                              B2 rudimentary                                            Downstream
                                 terminator                                             terminator
```

The distance between rudimentary and downstream terminators is 49 bp.

H. B2 copy coordinates: chr18:67278108-67278458

```
               10        20        30        40        50        60        70        80        90
   1.: GTACTTGTATACTTTAAATAAATAAATAAATAAATAATACAAACTTTAAAATTAAAAAGATGTCCCTATCACACTGCCAGATGTGAAACG
   2.: GTACTTGTATACTTTAAATAAATAAATAAATAAATAATACAAACTTTAAAATTAAAAAGATGTCCCTATCACACTGCCAGATGTGAAACG
   3.: GTACTTGTATACTTTAAATAAATAAATAAATAAATAATACAAACTTTAAAATTAAAAAGATGTCCCTATCACACTGCCAGATGTGAAACG
   4.: GTACTTGTATACTTTAAATAAATAAATAAATAAATAATACAAACTTTAAAATTAAAAAGATGTCCCTATCACACTGCCAGATGTGAAACG
   5.: GTACTTGTATACTTTAAATAAATAAATAAATAAATAATACAAACTTTAAAATTAAAAANATGTCCCTATCACACTGCCAGATGTGAAACG
   6.: GTACTTGAATACTTTAAATAAATAAATAAATAAATAATACAAACTTTAAAATTAAAAAGATGTCCCTATCACACTACCAGATGTGAAACG
 chr18: GTACTTGTATACTTTAAATAAATAAATAAATAAATAATACAAACTTTAAAATTAAAAAGATGTCCCTATCACACTGCCAGATGTGAAACG
```

```
          3’-end region of B2                   TTT
                                             B2 rudimentary
                                              terminator
                                                             The distance between rudimentary and


                100       110       120       130       140       150       160       170
     1.: AAAGAAAACAGTTGGGATTACTACAACGTCATGTAAGTGTGAAGACAAGACCACTGTT----AAAAAAAAAAAAAAAAAATCGGAA
     2.: AAAGAAAACAGTTGGGATTACTACAACGTCATGTAAGTGTGAAGACAAGACCACTGTT----AAAAAAAAAAAAAAAAAATCGGAA
     3.: AAAGAAAACAGTTGGGATTACTACAACGTCATGTAAGTGTGAAGACAAGACCACTGTTAAAAAAAAAAAAAAAAAAAGATCGGAA
     4.: AAAGAAAACAGTTGGGATTACTACAACGTCATGTAAGTGTGAAGACAAGACCACTGTTTAAAAAAAAAAAAAAAAAAAAAAAAAA
     5.: AAAGAAAACAGTTGGGATTACTACAACGTCATGTAAGTGTGAAGACAAGACCACTGTTTA-TAAAAAAAAAAAAAAAGATCGGAA
     6.: AAAGAAAACAGTTGGGATTACTACAACGTCATGTAAGTGTGAAGACAAGACCACTGTTTTAAAAAAAAAAAAAAAAAAAAAATAT
  chr18: AAAGAAAACAGTTGGGATTACTACAACGTCATGTAAGTGTGAAGACAAGACCACTGTTTTGTTATGCTGAGGCAGGTTCTGCTAG
                                                          TGTTTT    poly(A)
                                                        Downstream
                                                        Terminator
         downstream terminators is 97 pb
```

I. B2 copy coordinates: chr19:8719168_8719337

```
                 10        20        30        40        50        60        70        80        90
     1.: GTGTACTCACACAAAATAAATAAATCT---AAAAAAAAGTTCTTTGTTTGCTTGTTTGTTTGTTTTT---AAAAAAAAAAAAAAACATCGGAA
     2.: GTGTACTCACACAAAATAAATAAATCT---AAAAAAAAGTTCTTTGTTTGCTTGTTTGTTTGTTTT----AAAAAAAAAAAAAAAGATCGGAA
     3.: GTGTACTCACACAAAATAAATAAATCT---AAAAAAAAGTTCTTTGTTTGCTTGTTTGTTTGTTT-----AAAAAAAAAAAAAAAGATCGGAA
     4.: GTGTACTCACACAAAATAAATAAATCTAAAAAAAAAAAGTTCTTTGTTTGCTTGTTTGTTTGTTTTTGAAAAAAAAAAAAAAAAAAATCGGAA
     5.: GTGTACTCACACAAAATAAATAAATCT----AAAAAAAGTTCTTTGTTTGCTTGTTTGTTTGTTTTTAAAAAAAAAAAAAAAAAAGATCGGAA
     6.: GTGTACTCACACAAAATAAATAAATCT---AAAAAAAAGTTCTTTGTTTGCTTGTTTGTTT---------AAAAAAAAAAAAAAAAATCCGGA
     7.: GTGTACTCACACAAAATAAATAAATCT-AAAAAAAAAAGTTCTTTGTTTGCTTGTTTGTTT-------AAAAAAAAAAAAAAAAAAACCGGAA
     8.: GTGTACTCACACATACTACATAAATCT---AAAAAAAAGTTCTTTGTTTGCTTGTTTGTT--------AAAAAAAAAAAAAAAAAATTCGGAA
     9.: GTGTACTCACACAAAATAAATAAATCT---AAAAAAAAGTTCTTTGTTTGCTTGTTTGTTTGTTTT---AAAAAAAAAAAAAAAAAACAAAAA
  chr19: GTGTACTCACACAAAATAAATAAATCT---AAAAAAAAGTTCTTTGTTTGCTTGTTTGTTTGTTTTTGAGACAGGGTTTCTCTGT--------
          3’-end region of B2     TCT                             TTTGTTTTT        poly(A)
                               B2 rudimentary                    Downstream
                                terminator                        terminator

                        The distance between rudimentary and downstream terminators is 28 bp.
```

Figure S6. B2 copies without their own functional terminators that are transcribed by pol III to downstream terminators and the resulting transcripts can be polyadenylated. Figures A through I demonstrate nine such examples. The alignments demonstrate the sequences of transcripts (reads from the L929 cell library) mapped to these loci in the mouse genome. The bottom line (highlighted in yellow) of each alignment displays the 3'-terminal region of the B2 copy and the flanking genomic sequence. The truncated terminator sequences in the reads, which are indicative of pol III transcription, are highlighted in green. The distances between the rudimentary B2 terminator and the downstream terminator are 36 bp (A), 82 bp (B), 50 bp (C), 75 bp (D), 52 bp (E), 39 bp (F), 49 bp (G), 97 bp (H), and 28 bp (I).
