# Supplementary material for: Analysis of RNA Transcribed by RNA Polymerase III from B2 SINEs in Mouse Cells
Source: Noncoding RNA. 2025 May 14;11(3):39. doi: 10.3390/ncrna11030039 (PMC12101331; doi:10.3390/ncrna11030039)
Supplement: Supplementary file 1 [file ncrna-11-00039-s001.zip › ncrna-3586305-supplementary/Figure S7.pdf]

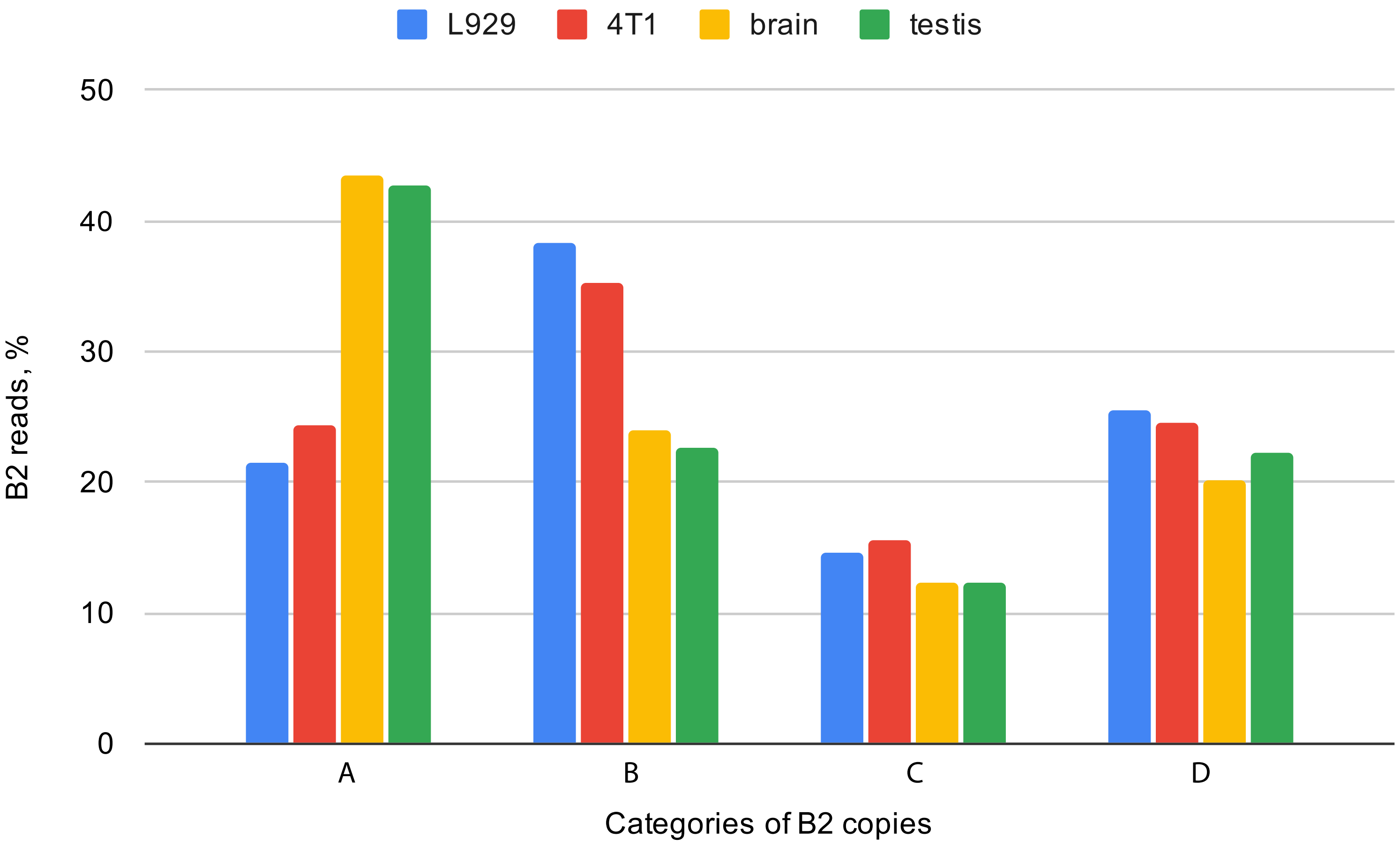

Figure S7. Distribution of reads by B2 copy categories. Reads from libraries prepared using Method 2 were analyzed for L929 and 4T1 cell cultures, as well as for mouse brain and testes. Data from three B2 copy datasets (Tables S3, S4, and S5) were combined.
