# Supplementary material for: Analysis of RNA Transcribed by RNA Polymerase III from B2 SINEs in Mouse Cells
Source: Noncoding RNA. 2025 May 14;11(3):39. doi: 10.3390/ncrna11030039 (PMC12101331; doi:10.3390/ncrna11030039)
Supplement: Supplementary file 1 [file ncrna-11-00039-s001.zip › ncrna-3586305-supplementary/Table S2.pdf]

Table S2. Analysis of the number of reads obtained by NGS of B2 cDNA libraries

I	II	III	IV	V	VI
Cell lines or tissues	Library preparation Method, #	Raw reads	All B2 reads	Uniquely mapped B2 reads**	Reads mapped to B2 copies that are clearly transcribed ***
L929	1	9,291,614	8,831,609	3,310,319	2,675,500
4T1	1	17,926,244	15,228,364	6,123,292	4,777,155
3T3-MHV *	1	16,160,698	16,108,002	5,620,407	4,348,858
L929	2	8,215,174	7,924,411	4,679,482	4,665,525
4T1	2	8,480,371	7,685,404	4,970,301	4,864,644
brain	2	9,571,401	8,355,205	5,229,997	5,086,241
testis	2	10,518,409	9,294,294	6,695,735	6,564,682

*) Data obtained by Karijolich and colleagues [37] were analyzed.

**) Alignments were filtered by a minimum match length of 100 bp and a quality score of ≥25, thus retaining only uniquely mapping reads.

***) Similar to column V, except that B2 copies with 10 or fewer reads per copy were excluded. Both L929 libraries were the smallest (2,675,500 and 4,665,525 reads for Methods 1 and 2, respectively) and were used to normalize read numbers of other libraries.
