# Supplementary material for: Analysis of RNA Transcribed by RNA Polymerase III from B2 SINEs in Mouse Cells
Source: Noncoding RNA. 2025 May 14;11(3):39. doi: 10.3390/ncrna11030039 (PMC12101331; doi:10.3390/ncrna11030039)
Supplement: Supplementary file 1 [file ncrna-11-00039-s001.zip › ncrna-3586305-supplementary/Table S3.pdf]

Table S3. Sample 1 of B2 copies identified by analysis of cDNA libraries obtained by method 2 for L929, 4T1, brain, and testes cells in mouse. The numbers (N) of reads corresponding to each B2 copy in each library are indicated with the maximum number of reads highlighted in blue. The sample contains B2 copies ranking 1–100 in the list arranged by the total number of reads in the four libraries (see Table S1, worksheet 2).

Position, #	B2 copy (coordinates in the mouse genome)	Cells L929 (N of reads)	Cells 4T1 (N of reads)	Brain (N of reads)	Testis (N of reads)	Total for four libraries	Polyadenylation, gene/intergenic*	B2 copy category **
1	chr7:19933825_19933996	203843	607448	68528	4617	884436	intergenic	D
2	chr5:34259453_34259632	271438	6018	3793	1144	282393	intronic (–)	D
3	chr11:107478051_107478227	212311	3738	4256	2448	222753	PA, intergenic	C
4	chr1:52600321_52600494	15273	180912	2670	1198	200053	PA, intergenic	C
5	chr11:97993976_97994153	23409	53069	46055	67195	189728	PA, intronic (–)	A
6	chr10:128332614_128332787	1	6272	7916	172790	186979	PA, intergenic	B
7	chr15:12203365_12203538	116699	13956	448	4235	135338	intergenic	D
8	chr7:132858284_132858460	82371	20867	6119	9030	118387	PA, intronic (+)	C
9	chr17:24469558_24469725	4158	28382	34403	39303	106246	PA, intergenic	A
10	chr2:73048507_73048674	77824	566	3659	18457	100506	intergenic	D
11	chr9:70025950_70026123	44390	46121	855	707	92073	PA, intergenic	A
12	chr19:7053346_7053527	86010	2989	259	295	89553	intergenic	D
13	chr5:65535644_65535823	25469	28180	27480	2807	83936	intronic (–)	D
14	chr8:123625729_123625916	9096	24008	5162	35909	74175	intronic (–)	D
15	chr6:30343890_30344062	66808	5281	278	435	72802	intergenic	D
16	chr2:34801678_34801857	24727	27628	3823	13575	69753	exon (+)	D
17	chr15:98986258_98986418	12999	8510	39191	1834	62534	intronic (–)	D
18	chr1:72564357_72564528	39021	19728	400	59	59208	intronic (–)	D
19	chr8:25211394_25211564	9982	5198	882	41863	57925	PA, intronic (+)	C
20	chr11:4326404_4326579	34948	10161	861	4363	50333	intergenic	D
21	chr5:67434332_67434497	610	46497	1321	1239	49667	intronic (–)	D
22	chr17:80489756_80489931	43564	1690	270	247	45771	PA, intergenic	C
23	chr2:73256952_73257126	4601	33968	2034	2838	43441	intergenic	D
24	chr2:91237872_91238048	13744	4009	23643	1693	43089	PA, exonic (+)	B
25	chr4:8645036_8645211	3411	5349	1594	30893	41247	intergenic	D
26	chr2:83727848_83728026	909	1158	37425	811	40303	intronic (–)	D
27	chr4:155328675_155328852	121	450	38600	253	39424	PA, intronic (–)	A
28	chr16:8769356_8769525	8242	23640	6007	599	38488	intronic (–)	D
29	chr8:114334849_114335024	606	2824	22387	12569	38386	PA, intronic (–)	C
30	chr17:71319328_71319486	2180	33309	2704	186	38379	intronic (–)	D
31	chr4:62479708_62479888	132	27506	2927	6330	36895	intergenic	D
32	chr16:36859385_36859563	620	3559	12310	19955	36444	PA, exonic (+)	B
33	chr8:119978729_119978905	34881	295	736	195	36107	intronic (–)	D
34	chr1:133036101_133036282	6653	0	8667	20212	35532	PA, intergenic	A
35	chr11:20145970_20146148	551	178	1674	32940	35343	PA, intronic (–)	A
36	chr11:75483573_75483748	20810	4959	7014	1803	34586	PA, intergenic	C
37	chr17:32112666_32112839	21141	2234	6688	4393	34456	PA, intergenic	A
38	chr11:119015011_119015207	557	27274	1618	4231	33680	PA, intergenic	A
39	chr1:131985653_131985826	5151	4763	12004	11285	33203	PA, intronic (–)	C
40	chr2:91237486_91237655	4839	6162	17417	4339	32757	PA, intronic (+)	C
41	chr15:98890332_98890521	650	572	900	30598	32720	PA, intronic (+)	B
42	chr10:71303131_71303280	1114	3109	26466	1877	32566	PA, intergenic	A
43	chr5:130255747_130255918	1645	18532	11309	532	32018	PA, intronic (–)	A
44	chr9:110796539_110796715	15346	3009	1897	11765	32017	PA, intergenic	C
45	chr2:33746012_33746187	19992	10709	871	240	31812	PA, intronic (–)	C
46	chr1:156021511_156021688	647	3537	3910	23321	31415	PA, intronic (+)	A
47	chr12:59114758_59114930	1393	2009	940	27044	31386	PA, intronic (+)	C
48	chr8:112020268_112020444	978	5909	12308	11440	30635	exonic (+)	D
49	chr6:99211284_99211458	1551	4200	6444	17244	29439	intronic (+)	D
50	chr10:93558154_93558332	15791	2790	2931	7860	29372	intronic (–)	D
51	chr2:160582773_160582942	412	135	475	28158	29180	PA, Intronic (+)	C

52	chr11:54878426_54878602	21580	1405	2978	2645	28608	PA, intergenic	B
53	chr17:27674611_27674785	190	1202	698	26298	28388	PA, intronic (+)	A
54	chr3:103904188_103904361	11058	1664	1758	12038	26518	intronic (–)	D
55	chr13:64239757_64239938	869	3446	21614	213	26142	PA, intronic (–)	A
56	chr6:145216357_145216524	1573	4307	11040	9141	26061	exonic (+)	D
57	chr2:23040767_23040945	3609	2240	1422	18207	25478	PA, intergenic	B
58	chr3:31056728_31056895	7922	10162	4774	2566	25424	intergenic	D
59	chr4:127076755_127076933	7411	10460	3643	3763	25277	PA, intergenic	B
60	chr2:119609509_119609685	457	7404	2719	14521	25101	exonic (+)	D
61	chr4:129333249_129333424	1502	2185	2052	19319	25058	intronic (+)	D
62	chr15:34351608_34351785	691	24216	43	25	24975	PA, intronic (+)	A
63	chr2:32853715_32853892	4115	7593	10441	2747	24896	PA, intergenic	A
64	chr4:147939511_147939678	22684	252	1308	612	24856	intergenic	D
65	chr18:35053391_35053564	539	180	234	23701	24654	intronic (+)	D
66	chr13:58147755_58147919	3090	6731	12460	2372	24653	PA, intronic (–)	A
67	chr2:91224640_91224817	2255	1846	3691	16099	23891	PA, intronic (+)	B
68	chr2:30059703_30059879	12514	7043	2324	1927	23808	PA, intergenic	C
69	chr19:5789710_5789886	15338	6104	496	1600	23538	PA, intergenic	C
70	chr9:53608950_53609125	11502	6367	4031	1494	23394	intronic (+)	D
71	chr7:120953860_120954039	1615	3151	16608	1678	23052	PA, intergenic	A
72	chr15:102348646_102348820	6335	8068	3748	4797	22948	PA, intronic (–)	A
73	chr12:52595880_52596052	9734	5143	3554	4366	22797	PA, intergenic	A
74	chr11:53484757_53484935	4217	3027	3556	11984	22784	PA, intergenic	B
75	chr15:81742650_81742827	1901	2982	5072	12637	22592	PA, intergenic	B
76	chr7:36273212_36273394	335	359	19307	2583	22584	PA, intergenic	B
77	chr19:4820961_4821138	6939	1398	13284	437	22058	PA, intergenic	B
78	chr11:61582396_61582567	1036	398	7139	13401	21974	intergenic	D
79	chr7:126297804_126297995	6034	3816	10705	1412	21967	PA, intergenic	A
80	chr9:75536252_75536432	2465	3134	10785	5492	21876	PA, intronic (+)	C
81	chr2:156194117_156194273	6922	1454	4341	8883	21600	PA, intergenic	C
82	chr6:47912228_47912402	9	14	3282	18254	21559	intergenic	D
83	chr9:70023888_70024060	13508	2996	1177	3855	21536	intergenic	D
84	chr15:102450676_102450850	19601	1104	123	628	21456	intronic (–)	D
85	chr8:123900000_123900172	3442	128	15007	2780	21357	PA, intronic (–)	A
86	chr12:70914303_70914479	7283	1653	3175	9147	21258	PA, exonic (+)	A
87	chr19:34886572_34886753	929	5498	3508	11240	21175	PA, intergenic	A
88	chr2:93911911_93912087	139	11829	9100	105	21173	PA, intronic (+)	A
89	chr16:20473671_20473830	2223	1108	3164	14620	21115	intergenic	D
90	chr2:181593580_181593756	3236	3593	7198	6712	20739	intronic (–)	D
91	chr12:81830656_81830834	136	19918	152	432	20638	PA, intronic (+)	A
92	chr19:37104852_37105024	353	19433	632	128	20546	PA, Intronic (–)	C
93	chr16:91507245_91507423	780	5725	8030	5939	20474	exonic (+)	D
94	chr11:54879198_54879374	14360	5795	131	119	20405	intergenic	D
95	chr2:127115852_127116031	72	601	18921	309	19903	PA, intronic (–)	A
96	chr10:31526257_31526420	6471	5160	7378	656	19665	intronic (–)	D
97	chr7:79830616_79830792	4961	3286	1674	9439	19360	intronic (–)	D
98	chr3:108256179_108256356	1646	13192	2145	1943	18926	PA, intergenic	B
99	chr7:28339334_28339507	2697	3671	2862	9648	18878	intergenic	D
100	chr10:127115162_127115339	4104	6875	4767	3123	18869	PA, intronic (+)	B

*The localization of B2 copies relative to genes is as follows:
intergenic, 45%;
intronic (–) (opposite transcription of B2 and gene), 29%;
intronic (+) (unidirectional B2 and gene), 18%;
exonic (+), 8%.
**Distribution of B2 copies within the sample is categorized as follows:
category A (B2 with an efficient long terminator, green PA), 26%;
category B (B2 with a minimal TCTTT terminator and another terminator in the far downstream sequence, yellow PA), 14%;
category C (B2 with a rudimentary terminator and a nearby full-length terminator, brawn PA), 18%;
category D (B2 with a rudimentary terminator and a distant (>60 bp) functional terminator, no PA), 42%.
