# Supplementary material for: Analysis of RNA Transcribed by RNA Polymerase III from B2 SINEs in Mouse Cells
Source: Noncoding RNA. 2025 May 14;11(3):39. doi: 10.3390/ncrna11030039 (PMC12101331; doi:10.3390/ncrna11030039)
Supplement: Supplementary file 1 [file ncrna-11-00039-s001.zip › ncrna-3586305-supplementary/Table S4.pdf]

Table S3. Sample 2 of B2 copies identified by analysis of cDNA libraries obtained by method 2 for L929, 4T1, brain, and testes cells in mouse. The numbers (N) of reads corresponding to each B2 copy in each library are indicated with the maximum number of reads highlighted in blue. The sample contains B2 copies ranking 501–600 in the list arranged by the total number of reads in the four libraries (see Table S1, worksheet 2).

Position #	B2 copy (coordinates in the mouse genome)	Cells L929 (N of reads)	Cells 4T1 (N of reads)	Brain (N of reads)	Testis (N of reads)	Total for four libraries	Polyadenylation, gene/intergenic*	B2 copy category **
501	chr11:79004605_79004782	681	692	458	4117	5948	PA, intergenic	A
502	chr17:35884265_35884416	550	1337	3228	829	5944	PA, intronic (–)	A
503	chr19:22996890_22997063	178	774	1611	3378	5941	PA, intronic (+)	A
504	chr5:135992569_135992736	1209	999	3600	127	5935	PA, intergenic	A
505	chr5:90262416_90262581	814	2940	1383	796	5933	intronic (+)	D
506	chr2:92837227_92837397	1996	883	1785	1268	5932	intergenic	D
507	chr14:56860287_56860460	737	456	943	3794	5930	PA, intergenic	C
508	chr7:35268960_35269138	105	82	364	5370	5921	PA, intronic (–)	A
509	chr11:105440478_105440652	95	697	614	4502	5908	intronic (–)	D
510	chr1:180551426_180551612	5418	4	477	7	5906	PA, intergenic	C
511	chr4:123755335_123755511	622	4486	357	414	5879	intergenic	D
512	chr11:120770388_120770557	410	1080	938	3438	5866	PA, intergenic	B
513	chr18:61929511_61929683	4	7	1518	4313	5842	PA, intergenic	B
514	chr15:77909793_77909968	685	733	202	4219	5839	PA, intergenic	A
515	chr10:81017883_81018063	2260	1037	695	1839	5831	intergenic	D
516	chr1:132236281_132236456	68	87	60	5609	5824	PA, intronic (–)	B
517	chr1:157506136_157506305	2800	253	760	2011	5824	PA, intronic (–)	B
518	chr11:75674444_75674621	497	3098	1007	1220	5822	exonic (+)	D
519	chr19:20421488_20421671	1746	2940	213	910	5809	PA, intronic (–)	C
520	chr11:113580275_113580451	218	95	5398	97	5808	PA, intronic (+)	A
521	chr17:5215823_5216000	91	1754	2132	1823	5800	PA, intronic (–)	A
522	chr15:89443713_89443880	65	192	1362	4163	5782	PA, intergenic	B
523	chr1:189222724_189222901	225	1004	3539	1014	5782	PA, intronic (–)	A
524	chr11:23607555_23607724	130	845	3411	1395	5781	PA, intronic (–)	B
525	chr11:86654981_86655150	5777	0	0	0	5777	PA, intronic (–)	C
526	chr13:12236096_12236265	75	408	3228	2061	5772	PA, intronic (–)	A
527	chr11:44464873_44465031	136	2287	1759	1585	5767	PA, intronic (–)	A
528	chr12:111734006_111734179	741	748	2357	1912	5758	exonic (+)	D
529	chr19:29673863_29674033	177	447	485	4648	5757	PA, intergenic	A
530	chr9:70557396_70557550	458	425	3273	1596	5752	intronic (+)	D
531	chr17:47033299_47033466	3760	1281	295	407	5743	PA, intergenic	C
532	chr9:73092559_73092740	1701	810	324	2907	5742	PA, intronic (–)	A
533	chr3:95116525_95116699	4	11	1879	3848	5742	PA, intronic (–)	C
534	chr11:66891958_66892135	223	297	4260	955	5735	PA, intergenic	A
535	chr6:52143289_52143462	2239	1422	225	1836	5722	PA, intergenic	A
536	chr2:181666443_181666616	2284	987	1151	1298	5720	PA, intergenic	C
537	chr17:36935204_36935377	984	1639	1893	1233	5713	PA, intergenic	A
538	chr3:131198379_131198557	1085	1135	3176	309	5705	PA, intronic (–)	A
539	chr3:157694664_157694842	58	316	482	4831	5687	PA, intergenic	A
540	chr14:21679225_21679401	1273	1499	1973	937	5682	intronic (–)	D
541	chr3:31060812_31060982	561	1457	151	3511	5681	PA, intergenic	B
542	chr7:140924359_140924536	59	293	315	5012	5679	PA, Intronic (–)	A
543	chr8:124920216_124920392	67	2610	955	2047	5679	intronic (–)	D
544	chr9:70557203_70557380	458	345	3273	1596	5672	PA, intronic (+)	B
545	chr10:63325242_63325422	204	927	1680	2844	5655	PA, intronic (+)	C
546	chr16:30953775_30953958	1233	1163	1987	1259	5642	PA, intergenic	B
547	chr2:128816775_128816953	3532	153	1299	639	5623	PA, intergenic	B
548	chr10:12964881_12965068	264	1076	1321	2956	5617	PA, intergenic	B
549	chr19:36957006_36957180	389	1485	2371	1369	5614	PA, intronic (+)	C
550	chr2:32855058_32855223	1837	768	2478	517	5600	PA, intergenic	A
551	chr5:122998184_122998338	29	9	4074	1474	5586	exonic (+)	D
552	chr14:99075226_99075395	432	2244	1945	962	5583	PA, intergenic	B

553	chr5:129944440_129944617	906	2865	1248	559	5578	PA, intronic (–)	B
554	chr10:80041936_80042105	2836	931	801	1007	5575	PA, intergenic	B
555	chr12:54724430_54724604	435	3458	1332	348	5373	PA, intergenic	C
556	chr8:120642489_120642664	215	1070	1795	2491	5571	intronic (–)	D
557	chr1:52112853_52113026	1204	1841	870	1655	5570	PA, intronic (–)	A
558	chr10:126954097_126954273	3478	349	216	1506	5549	PA, intergenic	B
559	chr6:148971024_148971196	407	863	360	3914	5544	PA, intergenic	C
560	chr11:62469896_62470070	1416	1542	660	1917	5535	PA, intronic (–)	B
561	chr8:111645356_111645522	399	71	2374	2688	5532	PA, intronic (–)	A
562	chr9:21232953_21233118	46	4815	140	512	5513	PA, intronic (–)	A
563	chr12:91395334_91395515	27	114	5128	239	5508	PA, intronic (+)	B
564	chr8:22405569_22405742	4258	1067	132	26	5468	intronic (+)	D
565	chr2:32081250_32081419	246	301	4132	782	5461	intronic (–)	D
566	chr10:119264248_119264423	368	139	105	4838	5450	intergenic	D
567	chr3:88178455_88178626	1761	1872	1596	214	5443	PA, intergenic	B
568	chr3:51829016_51829193	50	637	358	4393	5438	PA, Intronic (–)	A
569	chr1:180551672_180551858	5418	7	0	5	5430	PA, intergenic	C
570	chr11:43712990_43713159	436	932	3053	998	5419	intronic (+)	D
571	chr7:99508098_99508275	2988	1746	221	461	5416	PA, intergenic	B
572	chr14:21822529_21822702	301	368	3897	837	5403	PA, intergenic	A
573	chr19:36907735_36907906	285	431	3325	1357	5398	PA, intergenic	A
574	chr8:75014797_75014970	299	1588	2653	837	5377	PA, intronic (–)	A
575	chr1:184886494_184886672	121	4691	457	106	5375	PA, intergenic	A
576	chr4:44094290_44094446	137	5067	24	144	5372	PA, Intronic (–)	C
577	chrX:12848810_12848982	1093	1157	366	2744	5360	intergenic	D
578	chr3:51393230_51393403	1435	632	406	2872	5345	PA, intronic (–)	A
579	chr6:125008255_125008430	543	685	3253	859	5340	PA, exonic (–)	B
580	chr19:25022519_25022699	46	5072	137	85	5340	PA, intronic (–)	A
581	chr5:34260462_34260636	3111	781	160	1288	5340	PA, intronic (–)	A
582	chr15:98671487_98671663	26	74	126	5109	5335	PA, intronic (–)	A
583	chr19:30713778_30713956	343	577	2095	2316	5331	PA, intronic (+)	A
584	chr11:118234286_118234468	923	1431	2537	438	5329	PA, intronic (–)	A
585	chr5:123538512_123538689	20	1369	2008	1932	5329	PA, intronic (+)	B
586	chr5:33634991_33635160	7	12	812	4487	5318	exonic (+)	D
587	chr11:94659915_94660078	613	612	3418	663	5306	exonic (+)	D
588	chr6:128310467_128310642	297	392	195	4421	5305	intergenic	D
589	chr10:120463803_120463970	555	2898	1285	555	5293	PA, intronic (–)	C
590	chr2:59610223_59610382	4794	97	307	82	5280	PA, intergenic	C
591	chr4:134204227_134204408	8	61	188	5022	5279	PA, intergenic	B
592	chr6:50561779_50561953	291	165	500	4312	5268	intergenic	D
593	chr4:14947983_14948160	1098	1442	1595	1131	5266	PA, intronic (–)	A
594	chr4:133566808_133566985	0	17	2213	3036	5266	PA, intergenic	A
595	chr14:21763736_21763899	63	599	4339	244	5245	PA, intronic (–)	B
596	chr10:115981588_115981765	288	403	2687	1864	5242	PA, intergenic	B
597	chr15:78509100_78509263	34	17	197	4992	5240	PA, intergenic	A
598	chr8:69191763_69191926	29	134	4164	888	5215	PA, intergenic	A
599	chr3:94928618_94928792	0	5204	0	0	5204	PA, Intronic (–)	A
600	chr7:29283186_29283356	57	660	1145	3334	5196	intergenic	D

*The localization of B2 copies relative to genes is as follows:
intergenic, 45%;
intronic (–) (opposite transcription of B2 and gene), 37%;
intronic (+) (unidirectional B2 and gene), 12%;
exonic (+), 5%;
exonic (-), 1%.
**Distribution of B2 copies within the sample is categorized as follows:
category A (B2 with an efficient long terminator, green PA), 39%;
category B (B2 with a minimal TCTTT terminator and another terminator in the far downstream sequence, yellow PA), 24%;
category C (B2 with a rudimentary terminator and a nearby full-length terminator, brawn PA), 15%;
category D (B2 with a rudimentary terminator and a distant (>60 bp) functional terminator, no PA), 22%.
