# Supplementary material for: Analysis of RNA Transcribed by RNA Polymerase III from B2 SINEs in Mouse Cells
Source: Noncoding RNA. 2025 May 14;11(3):39. doi: 10.3390/ncrna11030039 (PMC12101331; doi:10.3390/ncrna11030039)
Supplement: Supplementary file 1 [file ncrna-11-00039-s001.zip › ncrna-3586305-supplementary/Table S5.pdf]

Table S5. Sample 3 of B2 copies identified by analysis of cDNA libraries obtained by method 2 for L929, 4T1, brain, and testes cells in mouse. The numbers (N) of reads corresponding to each B2 copy in each library are indicated with the maximum number of reads highlighted in blue. The sample contains B2 copies ranking 1051–1150 in the list arranged by the total number of reads in the four libraries (see Table S1, worksheet 2).

Position, #	B2 copy (coordinates in the mouse genome)	Cells L929, (N of reads)	Cells 4T1, (N of reads)	Brain (N of reads)	Testis (N of reads)	Total for four libraries	Polyadenylation, gene/intergenic*	B2 copy category **
1051	chr16:4872379_4872554	381	251	1153	1624	3409	intergenic	D
1052	chr10:61154600_61154778	206	781	379	2033	3399	PA, intergenic	B
1053	chr9:15391152_15391329	90	745	403	2159	3397	PA, intronic (+)	B
1054	chr4:152295077_152295251	2503	149	472	272	3396	intronic (–)	D
1055	chr8:110854902_110855080	423	2721	117	128	3389	PA, intronic (–)	A
1056	chr6:100821858_100822003	104	397	533	2351	3385	PA, no gene	A
1057	chr11:116071228_116071401	214	1429	157	1583	3383	intronic (+)	D
1058	chr5:140333366_140333532	700	1861	671	150	3382	PA, intronic (+)	A
1059	chr8:123902936_123903114	3033	103	188	57	3381	intronic (–)	D
1060	chr1:180820531_180820703	264	734	1107	1273	3378	PA, intergenic	B
1061	chr17:35042843_35043019	619	1172	1263	324	3378	PA, intronic (+)	B
1062	chr14:18183486_18183664	25	443	2260	645	3373	PA, intergenic	B
1063	chr10:127314948_127315124	791	1794	343	444	3372	PA, intergenic	A
1064	chr17:26252487_26252645	200	914	1769	487	3370	PA, intergenic	A
1065	chr2:17935284_17935448	217	970	154	2023	3364	PA, intergenic	C
1066	chr19:5786994_5787151	589	819	1896	59	3363	intergenic	D
1067	chr2:60413890_60414066	102	678	282	2298	3360	PA, intergenic	A
1068	chr6:116000243_116000414	1857	221	449	828	3355	intergenic	D
1069	chr4:135384717_135384883	53	252	414	2636	3355	PA, intronic (–)	A
1070	chr2:26144862_26145036	2236	314	671	133	3354	PA, intergenic	A
1071	chr4:44554747_44554923	43	231	991	2087	3352	PA, intronic (–)	A
1072	chr15:100624217_100624397	2016	185	265	885	3351	PA, intronic (+)	C
1073	chr16:22068685_22068864	915	2158	253	21	3347	intronic (–)	D
1074	chr4:129450915_129451077	358	550	1118	1316	3342	PA, intergenic	A
1075	chr8:13114332_13114508	108	755	1244	1234	3341	PA, intronic (+)	A
1076	chr18:61629958_61630141	488	425	735	1693	3341	PA, intronic (–)	C
1077	chr1:135704444_135704622	82	565	307	2386	3340	PA, intronic (–)	A
1078	chr5:136362293_136362447	201	714	2080	338	3333	intronic (+)	D
1079	chr4:116552756_116552930	445	877	420	1587	3329	PA, exonic (–)	B
1080	chr7:12932228_12932405	17	1648	951	704	3320	PA, intergenic	A
1081	chr19:36508634_36508810	719	1599	496	502	3316	PA, intergenic	A
1082	chr4:129101801_129101978	648	599	1446	620	3313	PA, intergenic	B
1083	chr8:122560895_122561046	501	1018	429	1361	3309	PA, intronic (–)	A
1084	chr13:12604991_12605167	78	124	872	2232	3306	PA, intronic (+)	B
1085	chr11:100572406_100572567	4	38	418	2843	3303	Last exonic (+)	D
1086	chr11:87335220_87335403	612	1195	228	1267	3302	PA, intronic (+)	A
1087	chr9:50928390_50928567	56	568	721	1952	3297	PA, intronic (+)	A
1088	chr4:129451974_129452144	69	998	418	1809	3294	PA, intronic (+)	A
1089	chr2:24939210_24939385	173	351	1825	938	3287	PA, intronic (+)	A
1090	chr12:52618478_52618654	1868	752	279	388	3287	Intronic (+)	D
1091	chr3:94617640_94617813	1718	942	160	465	3285	PA, intronic (+)	A
1092	chr11:121776220_121776393	530	963	312	1476	3281	PA, intergenic	A
1093	chr19:46008900_46009072	259	0	713	2305	3277	intronic (–)	D
1094	chrX:36886007_36886164	131	324	1362	1456	3273	PA, intronic (+)	B
1095	chrX:42131147_42131328	14	29	158	3071	3272	PA, intergenic	A
1096	chr1:163335329_163335506	1311	578	961	421	3271	PA, intergenic	B
1097	chr17:80055460_80055637	194	643	1410	1023	3270	PA, intronic (+)	A
1098	chr10:126905298_126905468	2660	245	133	232	3270	intergenic	D
1099	chr8:104326841_104327012	194	657	676	1741	3268	intronic (–)	D
1100	chr10:63374547_63374711	834	451	498	1484	3267	intronic (–)	D
1101	chr8:23405504_23405680	23	195	1922	1127	3267	PA, no gene	A
1102	chr6:46752724_46752900	624	0	144	2496	3264	intronic (–)	D
1103	chrX:101565617_101565794	166	921	680	1496	3263	PA, intronic (–)	A
1104	chr4:48628152_48628327	51	61	3050	100	3262	PA, intronic (+)	A
1105	chr9:64279193_64279362	1409	608	250	993	3260	intergenic	D

1106	chr19:42051327_42051499	1348	798	156	958	3260	intronic (–)	D
1107	chr11:84154421_84154588	2631	96	327	205	3259	PA, intronic (+)	C
1108	chr13:56360637_56360811	323	1538	268	1130	3259	PA, intergenic	C
1109	chr11:79154117_79154287	793	356	1210	894	3253	PA, intergenic	C
1110	chr2:75778850_75779021	2353	424	60	414	3251	intronic (–)	D
1111	chr3:97970740_97970916	120	213	177	2737	3247	PA, intergenic	C
1112	chr2:181372322_181372496	535	784	886	1035	3240	intronic (–).	D
1113	chr7:80837462_80837637	1861	871	54	453	3239	intergenic	D
1114	chr9:52057480_52057647	29	663	964	1583	3239	PA, intronic (+)	A
1115	chr8:106066261_106066437	47	1451	691	1049	3238	PA, intronic (–)	A
1116	chr1:86093886_86094058	328	905	887	1117	3237	PA, intronic (–)	A
1117	chr5:66198964_66199138	666	2408	57	106	3237	PA, intergenic	A
1118	chr14:57806671_57806842	387	1719	275	856	3237	exonic (+)	D
1119	chr5:142779904_142780078	770	603	1266	589	3228	intronic (–)	D
1120	chr13:99830672_99830886	259	296	1941	730	3226	PA, intergenic	A
1121	chr17:24133031_24133207	168	566	613	1879	3226	PA, intronic (+).	C
1122	chr1:135879373_135879546	280	436	2237	266	3219	PA, intronic (+)	A
1123	chr7:45275811_45275987	399	649	392	1776	3216	PA, intergenic	A
1124	chr2:180243561_180243738	351	101	104	2655	3211	PA, intergenic	B
1125	chr2:29886870_29887042	224	289	604	2093	3210	PA, intergenic	C
1126	chr14:75831115_75831289	470	694	575	1461	3200	PA, intergenic	A
1127	chr17:33695573_33695748	68	382	2100	648	3198	exonic (+)	D
1128	chr3:90286479_90286648	678	281	1344	894	3197	PA, intergenic	C
1129	chr12:78556332_78556501	340	1260	1034	562	3196	PA, intronic (+)	A
1130	chr11:74646519_74646695	189	598	247	2154	3188	intergenic	D
1131	chr2:153730677_153730853	296	593	792	1507	3188	PA, intergenic	A
1132	chr11:46617744_46617920	2	0	330	2855	3187	PA, Intronic (–)	B
1133	chr10:116933736_116933906	638	819	1479	248	3184	PA, Intronic (+).	C
1134	chr3:108694652_108694828	6	4	3164	9	3183	intronic (–)	D
1135	chr1:180971777_180971943	501	1736	637	306	3180	PA, intronic (–)	B
1136	chr5:21794116_21794277	660	1064	263	1192	3179	intronic (–)	D
1137	chr5:137543565_137543752	670	1377	377	744	3168	PA, intergenic	A
1138	chr7:127942827_127942999	2126	426	274	341	3167	intronic (–).	D
1139	chr12:103423213_103423388	62	1433	380	1290	3165	PA, intronic (–)	A
1140	chr7:24775788_24775963	128	530	375	2131	3164	PA, intronic (+).	B
1141	chr7:116309108_116309277	182	2060	364	554	3160	PA, intergenic	B
1142	chr14:30997801_30997977	227	505	1336	1092	3160	PA, intergenic	B
1143	chr1:74581933_74582112	369	1057	1321	406	3153	PA, intronic (+)	A
1144	chr17:62500425_62500598	1251	809	353	736	3149	PA, intergenic	C
1145	chr17:36006416_36006592	31	181	2380	555	3147	intronic (–)	D
1146	chr9:114870057_114870225	989	2008	74	75	3146	intergenic	D
1147	chr4:149582477_149582644	641	518	1737	250	3146	PA, intergenic	A
1148	chr9:70433817_70433993	203	1294	236	1411	3144	PA, intronic (–)	B
1149	chr11:87483929_87484105	971	998	993	182	3144	PA, intronic (–)	A
1150	chr16:33044395_33044575	95	2889	34	125	3143	PA, intronic (–)	C

*The localization of B2 copies relative to genes is as follows:
intergenic, 41%;
intronic (–) (opposite transcription of B2 and gene), 29%;
intronic (+) (unidirectional B2 and gene), 25%;
exonic (+), 5%.
**Distribution of B2 copies within the sample is categorized as follows:
category A (B2 with an efficient long terminator, green PA), 41%;
category B (B2 with a minimal TCTTT terminator and another terminator in the far downstream sequence, yellow PA), 17%;
category C (B2 with a rudimentary terminator and a nearby full-length terminator, brawn PA), 13%;
category D (B2 with a rudimentary terminator and a distant (>60 bp) functional terminator, no PA), 29%.
