# Supplementary material for: Analysis of RNA Transcribed by RNA Polymerase III from B2 SINEs in Mouse Cells
Source: Noncoding RNA. 2025 May 14;11(3):39. doi: 10.3390/ncrna11030039 (PMC12101331; doi:10.3390/ncrna11030039)
Supplement: Supplementary file 1 [file ncrna-11-00039-s001.zip › ncrna-3586305-supplementary/Table S6 and S7.pdf]

Table S6. Oligonucleotides utilized in preparation of B2 cDNA libraries by Method 1.

Name	Usage	Nucleotide sequence*
B2-specific primer 1	Primer extension for synthesis of the first strand of cDNA	5’ TGTAAGTACACTGTAGCTGTCTTCAGACA 3’
B2-specific primer 1-adapter (F)	PCR amplification of cDNA	5’AATGATACGGCGACCACCGAGATCTACACCGACGCTCTTCTGTAAGTACACTGTAGCTGTCTTCAGACA 3’
Oligo(dC)-containing primer -adapter (R)	PCR amplification of cDNA	5’CAAGCAGAAGACGGCATACGAGATTACAAGGTGACTGGAGTTCAGACGTGTGCTCTTCCGATCTCCCCCCCCCCCCCCC 3’
B2-seq oligo 1	Sequencing with MiSeq	5’CGACGCTCTTCTGTAAGTACACTGTAGCTGTCTTCAGACA 3’

*The sequence that is complementary to the B2 consensus sequence (positions 128–156) is underlined. The nucleotides shown in green extend the B2-specific primer compared to the original protocol [37]. The nucleotides shown in red were missing from the F-adapter sequence provided in the original protocol [37].

Table S7. Oligonucleotides utilized in preparation of B2 cDNA libraries by Method 2.

Name	Usage	Nucleotide sequence*
Oligo(dT)-containing primer	Primer extension for synthesis of the first strand of cDNA	5’ GACGTGTGCTCTTCCGATCTTTTTTTTTTTTTTTTT 3’
B2-specific primer 2-adapter (F)	PCR amplification of cDNA	5’AATGATACGGCGACCACCGAGATCTACACCGACGCTCTTCGGGCTGGAGAGATGGCTCAGTGGTT 3’
Primer-adapter (R)	PCR amplification of cDNA	5’CAAGCAGAAGACGGCATACGAGATTACAAGGTGACTGGAGTTCAGACGTGTGCTCTTCCGATCT 3’
B2-seq oligo 2	Sequencing with MiSeq	5’ GGGCTGGAGAGATGGCTCAGTGGTT 3’

* The sequence that is complementary to the B2 consensus sequence (positions 1–25) is underlined, while the sequence that is identical in the “Oligo(dT)-containing primer” and the “primer– adapter(R)” is shown in blue.
